# Synthesis and Biological Activity of Novel Polyazaheterocyclic Derivatives of Quinine

**DOI:** 10.3390/molecules30153301

**Published:** 2025-08-07

**Authors:** Gulim K. Mukusheva, Nurizat N. Toigambekova, Roza B. Seidakhmetova, Roza I. Jalmakhanbetova, Mukhlissa N. Babakhanova, Oralgazy A. Nurkenov, Ekaterina A. Akishina, Evgenij A. Dikusar, Irina A. Kolesnik, Hongwei Zhou, Vladimir I. Potkin

**Affiliations:** 1 Chemistry Faculty, Karaganda Buketov University, Karaganda 100024, Kazakhstan; mukushevagulim5@gmail.com (G.K.M.); nukonti92@mail.ru (N.N.T.); 2 Department of Clinical Pharmacology and Evidence-Based Medicine, Karaganda Medical University, Karaganda 100024, Kazakhstan; rozabat@mail.ru; 3 Faculty of Natural Sciences, L.N. Gumilyov Eurasian National University, Astana 010000, Kazakhstan; rozadichem@mail.ru (R.I.J.); muhlisa170920032003@gmail.com (M.N.B.); 4 Institute of Organic Synthesis and Coal Chemistry of the Republic of Kazakhstan, Karaganda 100008, Kazakhstan; nurkenov_oral@mail.ru; 5 Institute of Physical Organic Chemistry, National Academy of Sciences of Belarus, 220072 Minsk, Belarus; dikusar@ifoch.bas-net.by (E.A.D.); irynakolesnik93@gmail.com (I.A.K.); potkin@ifoch.bas-net.by (V.I.P.); 6 College of Biological, Chemical Sciences and Engineering, Jiaxing University, Jiaxing 314001, China; zhouhw@zju.edu.cn

**Keywords:** quinine, anabasine, cinchophen, methyl iodides, esters, triazole, azide-alkyne cycloaddition, isoxazole, isothiazole

## Abstract

A synthetic methodology of the CuAAC “click” approach was exploited for the construction of 1,2-azolyltriazole quinine derivatives by the reaction of O-propargylquinine with azidomethyl-1,2-azoles in methanol. Quinine–piperidine and quinine–anabasine conjugates were obtained using a chloroacetate linker by reacting quinine chloroacetate with piperidine or anabasine in a diethyl ether medium. Cinchophene ester was obtained by the acylation of quinine with cinchophen acid chloride in methylene chloride. The antibacterial, fungicidal, analgesic and cytotoxic properties of the obtained compounds were examined.

## 1. Introduction

The rational design of new chemical compounds by the covalent fusion of two or more pharmacophoric units with complementary activity and multiple pharmacological targets is an attractive strategy for the search for new promising broad-spectrum drugs. Different or similar mechanisms of action of the components of a hybrid drug can increase its effectiveness and reduce undesirable side effects. Previous studies on the synthesis and biological properties of 1,2-azole and pyridine acyl derivatives of quinine and anabasine have shown that some of them have a high level of antibacterial activity, but its manifestation depends on the type of pathogenic microorganism: the test strain *Staphylococcus aureus* was the most sensitive to almost all compounds. They can also be considered as promising antiviral agents and analgesics [1,2]. Thus, hybrid compounds where the alkaloid is covalently linked to heterocyclic pharmacophoric fragment can be considered as promising objects for studying their pharmacological properties.

The triazolyl group is becoming an increasingly popular linker in medicinal chemistry, and many different examples of the inclusion of this heterocycle in various drugs have appeared in the literature [3,4,5]. Monovalent copper-catalyzed azide–alkyne [3 + 2] cycloaddition (CuAAC) is the most important reaction within the concept of click chemistry and is currently one of the best methods for the efficient modification of complex natural products, allowing the binding of various pharmacophoric fragments to form a multifunctional 1,2,3-triazole heterocycle [6,7]. There is a limited number of papers on the synthesis of quinine derivatives, where the alkaloid fragment is linked via a triazole linker to various aromatic, heterocyclic and aliphatic fragments. Many of them have shown themselves to be promising antitumor, fungicidal, antimalarial and antiprotozoal agents [8,9,10,11,12]. However, there are no data on the synthesis of triazolyl derivatives of quinine with a 1,2-azole fragment, which is of undoubted interest.

This paper presents data on the synthesis and biological activity of polyheterocyclic quinine derivatives, including quinine–piperidine and –anabasine conjugates, quinine ester based on cinchophen (an analgesic, anti-inflammatory drug that increases renal excretion of uric acid) and a series of new triazole derivatives of quinine.

## 2. Results and Discussion

### 2.1. Chemistry

The synthetic protocol utilized for the synthesis of the anabasine–quinine hybrid was tested on the piperidine analogue **3**. The use of a chloroacetate linker was proposed. Thus, quinine chloroacetate **1** was obtained by the acylation of quinine with chloroacetyl chloride in diethyl ether in a 76% yield. Piperidine–quinine adduct **2** was obtained by reacting the obtained ester **1** with a twofold excess of piperidine in a diethyl ether medium with prolonged stirring (10 days). A similar method was used to synthesize anabasine–quinine adduct **3**. After column chromatography, target products **2** and **3** were isolated in 45 and 47% yields, respectively.

**Scheme 1:**
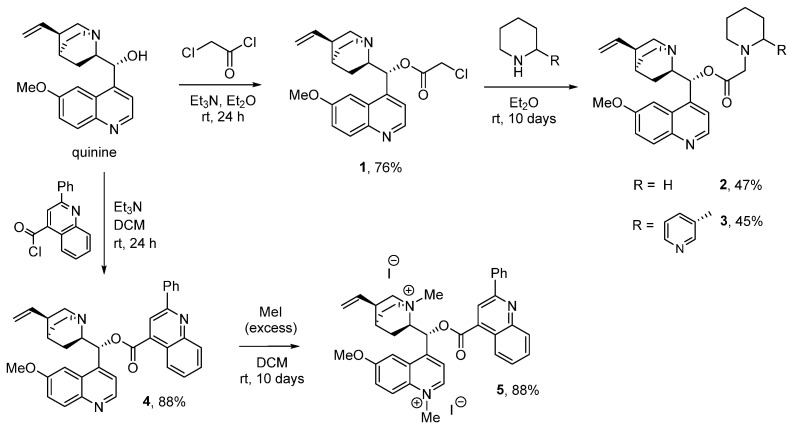
Synthesis of pyridine derivatives of quinine.

Cinchophene ester **4** was obtained by the acylation of quinine with cinchophen acid chloride in methylene chloride in the presence of triethylamine in an 88% yield. When compound **4** was kept in methylene chloride in the presence of excess methyl iodide, iodomethylate **5** was obtained in an 88% yield (Scheme 1).

**Scheme 2:**
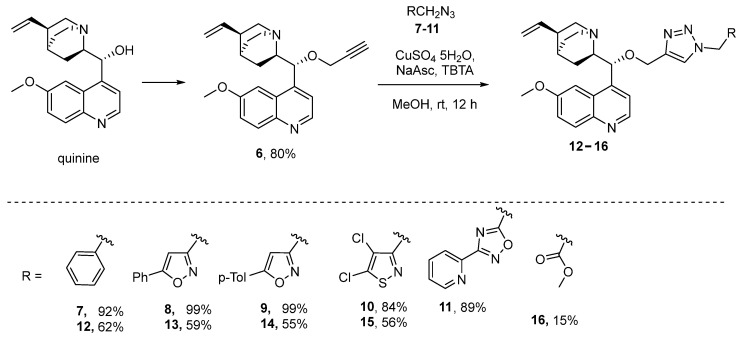
Synthesis of triazole derivatives of quinine.

A synthetic methodology of the CuAAC “click” approach was exploited for the construction of 1,2-azolyltriazole quinine derivatives **12**–**16** (Scheme 2). *O*-Propargylquinine **6** was synthesized by treating a solution of quinine in DMF with propagyl bromide in the presence of NaH at 0 °C in an 80% yield [13]. Azidomethyl-1,2-azoles were synthesized using the previous reported method [14]. The reaction was carried in the presence of copper sulphate, sodium ascorbate and TBTA in methanol at room temperature. The products **12**–**15** were isolated from the reaction mixture by column chromatography (yields 55–62%). It should be noted that when azide **11** was introduced into the CuAAC reaction, the process of 1,2,4-oxadiazole ring cleavage occurred to give compound **16** in a low yield (Scheme 2).

### 2.2. Bioactivity Screening

#### 2.2.1. Antimicrobial Activity

This study employed serial dilutions to determine the minimum inhibitory concentration (MIC) of the samples, which is the lowest concentration of an antimicrobial agent that inhibits the visible growth of microorganisms.

All tested samples were found to be the most sensitive to *Staphylococcus aureus*, but the MIC value for them was higher when compared to a reference antibiotic, ceftriaxone, which suggests the bacteria are more resistant to the novel quinine derivatives. In this case, the MICs were 21.6 and 45 μM. Compound **14** showed the greatest antibacterial activity. For test strains of *Bacillus subtilis*, the MIC of the tested compounds was between 21.6 and 124.7 μM. The antibacterial activity of the cinchophen (**5**) and (p-tolyl)isoxazolyltriazole (**14**) derivatives of quinine was at the same level as that of the comparison drug. None of the tested samples showed greater antibacterial activity against *Escherichia coli* than the reference drug (Table 1).

The minimum inhibitory concentration (Table 1) for compounds **5**, **12** and **14** was in the range of 59.5–100.8 μM, which is 4.4–7.5 times lower than that for the reference compound Nystatin (MIC = 13.4 μM).

Thus, in the series of novel derivatives of quinine, compound **14** showed the best results, demonstrating moderate activity against all test strains of microorganisms.

#### 2.2.2. Analgesic Activity In Vivo

The study of the analgesic activity of the novel quinine derivatives was carried out using the “vinegar writhing” test. Among the studied potential pharmaceutical compounds, the greatest effect was shown by anabasine–quinine hybrid **3**, cinchophen (**4**, **5**) and isothiazolyltriazole (**15**), causing a decrease in the writhing number by 41.2–45.6% (Table 2). Although the analgesic activity of these compounds did not reach the level of diclofenac sodium, it was comparable to it, amounting to about 53.3%.

Quinine derivatives **1**, **2** and **12**–**14** did not show significant analgesic activity in the “acetic writhing” test. The decrease in the amount of vinegar writhing in mice varied from 18.3 to 35.0%, which was much lower than in the case of the reference drug.

#### 2.2.3. Cytotoxicity Assays

Cytotoxicity was evaluated in a survival test of *Artemia salina* (Leach) marine crustacean larvae.

According to a prior study [15], which classified substances as toxic (LC50 value < 1000 μg/mL) and non-toxic (LC50 value > 1000 μg/mL), almost all the tested compounds, except for compounds **2**, **4** and **5**, showed good cytotoxic activity against *Artemia salina* (Table 3).

Triazole derivatives of quinine **12** were found to exhibit the highest cytotoxic activity against *Artemia salina* (Leach) among the tested compounds (LC_50_ was 77.9 μg/mL). The cytotoxic activity of **1**, **3** and **13**–**16** was comparable to that of quinine, and LC_50_ was in the range 82.2–102.1 μg/mL.

Although the brine shrimp bioassay is not specific for antitumor or any particular physiological action, it has the advantages of being rapid, inexpensive and simple and can be used for screening a significant number of species. Active compounds thus obtained could then be subjected to more specific assay systems [15].

## 3. Materials and Methods

The spectral analysis conditions, instrumentation and biotesting methodology are described in Supplementary Materials.

### Experimental Section

*(1R)-(6-Methoxyquinolin-4-yl)(2S,4S,5R)-5-vinylquinuclidin-2-yl) 2-chloroacetate* (**1**). A mixture of 0.64 g (2 mmol) of quinine, 0.35 g (3 mmol) of chloroacetic acid chloride and 0.6 g (3 mmol) of triethylamine in 100 mL of ether was stirred for 1 day. The mixture was washed with water and 5% NaHCO_3_ solution. The organic layer was dried over sodium sulfate. The solvent was removed completely under reduced pressure. The residue was recrystallized from a mixture of benzene and hexane (1:3) and dried in air at room temperature to give compound **1** as colorless crystals; yield 76% (0.61 g); mp 136–137 °C; [α]d^25^ −117°; IR (KBr) ν 3110, 3082, 3068, 3040, 3010, 2993, 2958, 2934, 2920, 2876, 2860, 2835, 1752 (C=Oest), 1634, 1620, 1597, 1508, 1475, 1453, 1434, 1411, 1363, 1354, 1329, 1301, 1259, 1229, 1195, 1165, 1134, 1098, 1085, 1025, 995, 978, 962, 950, 931, 913, 908, 849, 829, 807, 784, 768, 715, 680, 631, 613, 577, 520 cm^−1^; ^1^H NMR (CDCl_3_, 500 MHz) *δ* 1.50–1.58 (2H, m, CH_2_quinuc), 1.70–1.76 (1H, m, CHquinuc), 1.86–1.94 (2H, m, CH_2_quinuc), 2.27 (1H, br. s, CHquinuc), 2.58–2.69 (2H, m, CH_2_quinuc), 3.01–3.11 (2H, m, CH_2_quinuc), 3.39–3.44 (1H, dd, *J* = 16.3, 7.9 Hz, CHquinuc–N), 3.95 (3H, s, OCH_3_), 4.06–4.12 (2H, m, CH_2_Cl), 4.98–4.99 (1H, m, CH_2_=), 5.00–5.03 (1H, m, CH_2_=), 5.79–5.86 (1H, ddd, *J* = 17.6, 10.4, 7.4 Hz, –CH=), 6.51 (1H, d, *J* = 7.3 Hz, CH–O), 7.33–7.41 (3H, m, CHquinol), 8.01 (1H, d, *J* = 9.1 Hz, CHquinol), 8.73 (1H, d, *J* = 4.5 Hz, CHquinol); ^13^C NMR (CDCl_3_, 125 MHz) *δ* 24.54 (CH_2_quinuc), 27.61 (CHquinuc), 27.86 (CH_2_quinuc), 39.74 (CHquinuc), 40.95 (CH_2_Cl), 42.62 (CH_2_quinuc), 55.79 (OCH_3_), 56.71 (CH_2_quinuc), 59.18 (CHquinuc), 76.00 (CH–O), 101.41 (CHquinol), 114.74 (CH_2_=), 119.10 (CHquinol), 122.08 (CHquinol), 132.09 (CHquinol), 141.76 (–CH=), 147.58 (CHquinol), 127.00, 142.63, 145.00, 158.19, 166.63 (5Cquat); MS *m*/*z* (*I*_rel_, %) 401.10 [M + H]^+^ (100); anal. calcd. for C_22_H_25_ClN_2_O_3_ (400.90): C, 65.91; H, 6.29; Cl, 8.84; N, 6.99%; found: C, 66.29; H, 6.39; Cl, 8.45; N, 6.51%.

Procedure for the synthesis of piperidine– and anabasine–quinine adducts **2**, **3**. A solution of 1 g (2.4 mmol) of quinine chloroacetate **1** and 0.42 g (5 mmol) of piperidine or 0.81 g (5 mmol) of anabasine in 50 mL of dry ether was stirred at 20–23 °C for 10 days. The formed precipitate of piperidine or anabasine hydrochloride was separated by filtration on a porous glass filter and washed with a small amount of ether. The combined ether solutions of the product were washed with 5% NaHCO_3_ solution and dried over Na_2_SO_4_. The solvent was removed and the residue was purified by column chromatography on 100 μm silica gel (eluent: CH_2_Cl_2_/MeOH = 10:1).

*(1R)-(6-Methoxyquinolin-4-yl)(2S,4S,5R)-5-vinylquinuclidin-2-yl)methyl 2-(piperidin-1-yl)acetate* (**2**): yellow oil; yield 47% (0.51 g); [α]d^25^ −54°; IR (KBr) ν 3074, 3030, 2995, 2935, 2862, 2804, 1743 (C=Oest), 1621, 1592, 1570, 1509, 1474, 1454, 1433, 1396, 1360, 1303, 1284, 1260, 1242, 1229, 1161, 1130, 1111, 1085, 1070, 953, 914, 854, 832, 810, 795, 770, 717, 680, 670, 640, 615cm^−1^; ^1^H NMR (CDCl_3_, 500 MHz) *δ* 1.36–1.40 (2H, m, CH_2_piper), 1.48–1.57 (6H, m, CH_2_quinuc + _2_CH_2_piper), 1.68–1.74 (1H, m, CHquinuc), 1.84–1.90 (2H, m, CH_2_quinuc), 2.26 (1H, br. s, CHquinuc), 2.43–2.45 (4H, m, CH_2_piper), 2.56–2.65 (2H, m, CH_2_quinuc), 2.99–3.11 (2H, m, CH_2_quinuc), 3.18–3.26 (2H, q, *J* = 16.5 Hz, CH_2_C=O), 3.34–3.39 (1H, dd, *J* = 16.0, 7.8 Hz, CHquinuc–N), 3.94 (3H, s, CH_3_), 4.97–4.98 (1H, m, CH_2_=), 4.99–5.01 (1H, d, *J* = 10.4 Hz, CH_2_=), 5.78–5.85 (1H, ddd, *J* = 17.5, 10.4, 7.4 Hz, –CH=), 6.51–6.53 (1H, d, *J* = 7.4 Hz, CH–O), 7.32–7.37 (2H, m, CHquinol), 7.43–7.44 (1H, d, *J* = 2.6 Hz, CHquinol), 7.98–8.00 (1H, d, *J* = 9.2 Hz, CHquinol), 8.71–8.72 (1H, d, *J* = 4.5 Hz, CHquinol); ^13^C NMR (CDCl_3_, 125 MHz) *δ* 23.92 (CH_2_piper), 24.63 (CH_2_quinuc), 25.91 (2CH_2_piper), 27.69 (CHquinuc), 27.90 (CH_2_quinuc), 39.81 (CHquinuc), 42.56 (CH_2_quinuc), 54.37 (2CH_2_piper), 55.79 (OCH3), 56.71 (CH_2_quinuc), 59.24 (CHquinuc), 60.40 (CH_2_C=O), 73.81 (CH–O), 101.63 (CHquinol), 114.66 (CH_2_=), 119.14 (CHquinol), 121.97 (CHquinol), 131.90 (CHquinol), 141.86 (–CH=), 147.53 (CHquinol), 127.20, 143.66, 144.93, 158.08, 170.14 (5Cquat); MS *m*/*z* (*I*_rel_, %) 450.30 [M + H]^+^ (100); anal. calcd. for C_27_H_35_N_3_O_3_ (449.60): C, 72.13; H, 7.85; N, 9.35%; found: C, 72.36; H, 7.96; N, 9.25%.

*(R)-(6-methoxyquinolin-4-yl)((1S,2R,4S,5R)-5-vinylquinuclidin-2-yl)methyl 2-((S)-2-(pyridin-3-yl)piperidin-1-yl)acetate* (**3**): white crystals; yield 45% (0.57 g); [α]d^25^ −137°; mp 192–194 °C; IR (KBr) ν 2925, 2855, 1740 (C=Oest), 1622, 1592, 1570, 1508, 1473, 1431, 1262, 1240, 1228, 1229, 1163, 1135, 1099, 1026, 992, 914, 856, 806, 717 cm^−1^; ^1^H NMR (CDCl_3_, 500 MHz) *δ* 1.29–1.41 (2H, m, CH_2_piper), 1.42–1.58 (3H, m, CH_2_quinuc + CH_2_piper), 1.60–1.66 (2H, m, CH_2_piper), 1.65–1.73 (2H, m, CHquinuc + CH_2_piper), 1.74–1.84 (2H, m, CH_2_quinuc), 2.19–2.27 (1H, m, CHquinuc), 2.42–2.50 (1H, m, CH_2_piper), 2.50–2.63 (2H, m, CH_2_quinuc), 2.86–2.95 (2H, m, CH_2_quinuc + CH_2_piper), 2.99 (1H, d, *J* = 16.7 Hz, CH_2_C=O), 2.95–3.04 (2H, m, CH_2_quinuc), 3.23 (1H, d, *J* = 16.7 Hz, CH_2_C=O), 3.22–3.30 (1H, m, CHquinuc–N), 3.51 (1H, dd, *J* = 11.1, 2.7 Hz, CHpiper), 3.93 (3H, s, OCH_3_), 4.93–5.01 (2H, m, CH_2_=), 5.77 (1H, ddd, *J* = 17.5, 10.4, 7.4 Hz, –CH=), 6.44 (1H, d, *J* = 6.8 Hz, CH–O), 7.16 (1H, dd, *J* = 7.8, 4.8 Hz, CH_Py_), 7.26 (1H, d, *J* = 4.6 Hz, CH_Py_), 7.33–7.37 (2H, m, CHquinol), 7.64 (1H, dt, *J* = 7.8, 1.8 Hz, CHquinol), 8.00 (1H, dd, *J* = 9.8, 1.9 Hz, CHquinol), 8.45 (1H, dd, *J* = 4.8, 1.7 Hz, CH_Py_), 8.52 (1H, d, *J* = 1.7 Hz, CH_Py_), 8.69 (1H, d, *J* = 4.5 Hz, CHquinol); ^13^C NMR (CDCl_3_, 125 MHz) *δ* 24.23 (CH_2_piper), 24.76 (CH_2_quinuc), 25.98 (CH_2_piper), 27.59 (CHquinuc), 27.80 (CH_2_quinuc), 36.58 (CH_2_piper), 39.76 (CHquinuc), 42.60 (CH_2_quinuc), 53.91 (CH_2_piper), 55.80 55.79 (OCH_3_), 56.45 (CH_2_C=O), 56.71 (CH_2_quinuc), 59.17 (CHquinuc), 63.92 (CHpiper), 73.72 (CH–O), 101.45 (CHquinol), 114.67 (CH_2_=), 118.88 (CHquinol), 122.01 (CHquinol), 123.82 (CH_Py_), 131.94 (CHquinol), 135.17 (CH_Py_), 141.78 (–CH=), 147.52 (CHquinol), 149.10 (CH_Py_), 149.55 (CH_Py_), 127.03, 139.42, 143.66, 144.87, 158.08, 170.19 (6Cquat); anal. calcd. for C_32_H_38_N_4_O_3_ (526.68): C, 72.98; H, 7.27; N, 10.64%; found: C, 72.63; H, 7.51; N, 10.25%.

*(R)-(6-methoxyquinolin-4-yl)((1S,2R,4S,5R)-5-vinylquinuclidin-2-yl)methyl 2-phenylquinoline-4-carboxylate* (**4**). A mixture of 1.1 g (3.4 mmol) of quinine, 1.2 g (3.7 mmol) of 2-phenyl-4-quinolinecarbonyl chloride hydrochloride and 1.1 g (10 mmol) of triethylamine in 100 mL of dichloromethane was stirred for 1 day. The mixture was washed with water and 5% NaHCO_3_ solution and dried over Na_2_SO_4_. The solvent was removed. The residue was recrystallized from a mixture of benzene and hexane (1:3) and dried in air at room temperature to give compound **4** as colorless crystals; yield 88% (1.66 g); mp 76–78 °C; [α]d^25^ −45°; IR (KBr) ν 3063, 3032, 2995, 2932, 2880, 2862, 2830, 1727 (C=Oest), 1621, 1591, 1548, 1508, 1490, 1473, 1450, 1440, 1431, 1344, 1282, 1237, 1229, 1189, 1147, 1132, 1079, 1029, 1002, 960, 913, 850, 832, 795, 769, 612, 692, 652 cm^−1^; ^1^H NMR (CDCl_3_, 500 MHz) *δ* 1.58–1.67 (1H, m, CH_2_quinuc), 1.72 (1H, dd, *J* = 13.3, 7.6 Hz, CHquinuc), 1.77–1.86 (1H, m, CH_2_quinuc), 1.90–1.96 (1H, m, CH_2_quinuc), 2.01–2.10 (1H, m, CH_2_quinuc), 2.32 (1H, br. s, CHquinuc), 2.68 (1H, ddd, *J* = 13.9, 4.2, 2.4 Hz, CH_2_quinuc), 2.72–2.78 (1H, m, CH_2_quinuc), 3.09 (1H, dd, *J* = 13.9, 10.1 Hz, CH_2_quinuc), 3.22–3.33 (1H, m, CH_2_quinuc), 3.60–3.65 (1H, m, CHquinuc–N), 3.96 (3H, s, CH_3_), 5.00–5.06 (2H, m, CH_2_=), 5.87 (1H, ddd, *J* = 17.6, 10.4, 7.4 Hz, –CH=), 6.87 (1H, d, *J* = 7.3 Hz, CH–O), 7.43 (1H, dd, *J* = 9.2, 2.7 Hz, CHquinol), 7.49–7.54 (2H, m, 1CHcinchoph + 1CHquinol), 7.54–7.60 (3H, m, CHcinchoph), 7.62 (1H, d, *J* = 2.7 Hz, CHquinol), 7.74–7.79 (1H, m, CHcinchoph), 8.08 (1H, d, *J* = 9.1 Hz, CHquinol), 8.14–8.18 (2Hcinchoph, m), 8.21–8.25 (1Hcinchoph, m), 8.40–8.42 (1H, m, CHcinchoph), 8.63 (1H, dd, *J* = 8.6, 0.8 Hz, CHcinchoph), 8.78 (1H, d, *J* = 4.5 Hz, CHquinol); ^13^C NMR (CDCl_3_, 125 MHz) *δ* 25.16 (CH_2_quinuc), 27.69 (CHquinuc), 28.10 (CH_2_quinuc), 39.77 (CHquinuc), 42.67 (CH_2_quinuc), 55.82 (OCH_3_), 56.80 (CH_2_quinuc), 59.43 (CHquinuc), 75.90 (CH–O), 101.78 (CHquinol), 114.82 (CH_2_=), 119.44 (CHquinol), 120.11 (1CHcinchoph), 122.02 (CHquinol), 125.28 (1CHcinchoph), 127.57 (2CHcinchoph), 128.18 (1CHcinchoph), 129.20 (2CHcinchoph), 130.03 (1CHcinchoph), 130.28 (1CHcinchoph), 130.57 (1CHcinchoph), 132.23 (CHquinol), 141.78 (–CH=), 147.71 (CHquinol), 124.02, 127.20, 135.41, 138.86, 143.18, 145.16, 149.46, 156.85, 158.26, 165.69 (10Cquat); anal. calcd. for C_36_H_33_N_3_O_3_ (555.25): C, 77.81; H, 5.99; N, 7.56%; found: C, 78.02; H, 6.05; N, 7.29%.

*(1S,2R,4S,5R)-2-((R)-(6-methoxy-1-methylquinolin-1-ium-4-yl)((1-methyl-7-phenylquinolin-1-ium-4-carbonyl)oxy)methyl)-1-methyl-5-vinylquinuclidin-1-ium iodide* (**5**). To a solution of 0.42 g (0.756 mmol) of compound **4** in 30 mL of dichloromethane, 1 mL (16.1 mmol) of iodomethane was added. The mixture was kept in the dark for 10 days. The resulting precipitate of the target product was filtered off, washed with a small amount of dichloromethane and dried in air at room temperature to give dimethyliodide **5** as orange crystals; yield 88% (0.56 g); mp 192–194 °C; [α]d^25^ −6°; IR (KBr) ν 3080, 3055, 3030, 2998, 2926, 2885, 2855, 2830, 1738 (C=Oest), 1615, 1590, 1532, 1495, 1475, 1456, 1445, 1433, 1370, 1345, 1274, 1239, 1227, 1177, 1143, 1125, 1075, 1063, 1034, 1021, 992, 940, 917, 903, 826, 793, 769, 726, 710, 693, 676, 650 cm^−1^; ^1^H NMR (DMSO-d6, 500 MHz) δ 1.81–1.93 (1H, m, CH_2_quinuc), 2.12–2.22 (1H, m, CH_2_quinuc), 2.22–2.25 (1H, m, CHquinuc), 2.25–2.36 (1H, m, CH_2_quinuc), 2.73–2.82 (1H, m, CH_2_quinuc), 2.89–2.98 (1H, m, CHquinuc), 3.59 (3H, NCH_3_), 3.56–3.68 (1H, m, CH_2_quinuc), 3.77–3.86 (1H, m, CH_2_quinuc), 3.88–3.95 (1H, m, CHquinuc), 4.04–4.12 (1H, m, CH_2_quinuc), 4.13–4.20 (1H, m, CH_2_quinuc), 4.24 (3H, s, OCH_3_), 4.67 (3H, s, NCH_3_), 5.05–5.12 (1H, m, CH_2_=), 5.15–5.24 (1H, m, CH_2_=), 5.84 (1H, ddd, *J* = 17.6, 10.4, 7.4 Hz, –CH=), 7.44 (1H, s), 7.60–7.65 (1H, m), 7.67–7.76 (4H, m), 7.88–7.93 (1H, m), 8.08 (1H, dd, *J* = 9.7, 2.5 Hz), 8.24 (1H, d, *J* = 8.3 Hz), 8.37–8.42 (2H, m), 8.60 (1H, *J* = 3.9 Hz), 8.61 (1H, *J* = 7.6 Hz), 8.65 (1H, d, *J* = 8.1 Hz), 8.93–8.96 (1H, m), 9.36 (1H, d, *J* = 6.3 Hz); ^13^C NMR (DMSO-d6, 125 MHz) δ 21.16 (CH_2_quinuc), 25.14 (CH_2_quinuc), 26.58 (CHquinuc), 37.90 (CHquinuc), 46.43 (NCH_3_), 49.80 (NCH_3_), 54.56 (CH_2_quinuc), 57.17 (OCH_3_), 65.14 (CHquinuc), 65.24 (CH_2_quinuc), 70.29 (CH–O), 104.35 (CHquinol), 117.66 (CH_2_=), 120.72 (CHquinol), 120.79 (1CHcinchoph), 122.73 (CHquinol), 125.53 (1CHcinchoph), 127.84 (1CHcinchoph), 127.99 (2CHcinchoph), 129.00 (1CHcinchoph), 129.72 (2CHcinchoph), 130.56 (1CHcinchoph), 130.84 (1CHcinchoph), 131.17 (CHquinol), 138.25 (–CH=), 147.00 (CHquinol), 123.92, 127.64, 134.26, 134.70, 138.37, 149.00, 150.08, 156.57, 160.27, 164.17 (10Cquat); anal. calcd. for C_38_H_39_I_2_N_3_O_3_ (839.56): C, 54.36; H, 4.68; I, 30.23; N, 5.01%; found: C, 54.68; H, 4.77; I, 30.01; N, 4.69%.

Procedure for the synthesis of azidomethyl derivatives **10**, **11**. Sodium azide (6 mmol) was added to a solution of 5 mmol of the corresponding chloromethyl derivative [16,17] in 10 mL of dry DMF and the reaction mixture was stirred at 65 °C for 4 h, then poured into water saturated with NaCl. The product was extracted with dichloromethane and dried over sodium sulfate; the solvent was evaporated under reduced pressure.

*3-(Azidomethyl)-4,5-dichloroisothiazole* (**10**): yield 84% (0.88 g); oil; IR (KBr) ν 2927, 2854; 2178, 2103 (-N_3_); 1506, 1438, 1391, 1377, 1325, 1263, 1199, 1100, 991, 974, 808 cm^−1^; ^1^H NMR (CDCl_3_, 500 MHz) δ 4.43 (2H, s, CH_2_); ^13^C NMR (CDCl_3_, 125 MHz) δ 49.9 (CH_2_), 122.65 (C), 148.99 (C), 161.47 (C); anal. calcd. for C_4_H_2_Cl_2_N_4_S (209.05): C, 22.98; H, 0.96; Cl, 33.92; N, 26.80; S, 15.34%; found: C, 23.12; H, 1.15; Cl, 33.71; N, 26.69; S, 15.18%.

*5-(Azidomethyl)-3-(pyridin-2-yl)-1,2,4-oxadiazole* (**11**): yield 89% (0.90 g); yellow crystals; IR (KBr) ν 3394, 3058, 2955, 2927, 2160, 2111 (-N_3_), 1600, 587, 1573, 1521, 1475, 1435, 1426, 1386, 1352, 1306, 1255, 1208, 1149, 997, 894, 799, 762, 742, 719, 622 cm^−1^; ^1^H NMR (CDCl_3_, 500 MHz) δ 4.65 (2H, s, CH_2_), 7.41 (1H, ddd, *J* = 7.7, 4.8, 1.2 Hz, CH_Py_), 7.83 (1H, td, *J* = 7.8, 1.7 Hz, CH_Py_), 8.10 (1H, dt, *J* = 7.9, 1.0 Hz, CH_Py_), 8.74–8.78 (1H, m, CH_Py_); ^13^C NMR (CDCl_3_, 125 MHz) δ 45.92 (CH_2_), 123.41 (1CH_Py_), 125.93 (1CH_Py_), 137.25 (1CH_Py_), 150.58 (1CH_Py_), 145.75 (C), 168.52 (C), 174.82 (C); anal. calcd. for C_8_H_6_N_6_O (202.18): C, 47.53; H, 2.99; N, 41.57%; found: C, 47.62; H, 3.09; N, 41.46%.

Procedure for the synthesis of triazole derivatives **12**–**16**. To a solution of copper sulfate (0.05 mmol, 12.5 mg) in methanol, TBTA (0.1 mmol, 53 mg) was added and stirred until completely dissolved. Then sodium ascorbate (0.2 mmol, 40 mg) was added and stirred for another 10 min. To the resulting solution, azide (1 mmol) and O-propargylquinine (1 mmol, 362 mg) were added. The reaction mixture was stirred for 12 h, poured into salted water and extracted with methylene chloride. The product was purified by column chromatography (eluent EtOAc:MeOH 8:2 + 1% Et_3_N; Rf = 0.3 for **12**–**15**, Rf = 0.1 for **16**).

*(1S,2R,4S,5R)-2-((R)-((1-benzyl-1H-1,2,3-triazol-4-yl)methoxy)(6-methoxyquinolin-4-yl)methyl)-5-vinylquinuclidine* (**12**): light yellow powder; yield 62% (0.31 g); mp 58–59 °C [colorless oil, Lit.^8^]; [α]d^25^ −70°; IR (KBr) ν 3138, 3071, 3033, 2926, 2861, 1620, 1590, 1507, 1471, 1455, 1431, 1360, 1320, 1253, 1240, 1225, 1133, 1100, 1076, 1047, 1028, 991, 913, 853, 833, 820, 716, 703, 643 cm^−1^; ^1^H NMR (CDCl_3_, 500 MHz) δ 1.38–1.48 (1H, m, CH_2_quinuc), 1.50–1.67 (3H, m, CH_2_quinuc); 1.67–1.75 (1H, m, CHquinuc), 2.16–2.25 (1H, m, CH-CH=CH_2_), 2.48–2.64 (2H, m, CH_2_quinuc-N), 3.01 (1H, dd, *J* = 13.6, 10.2 Hz, CH_2_quinuc-N), 3.06–3.17 (1H, m, CHquinuc-N), 3.17–3.32 (1H, m, CH_2_quinuc-N), 3.88 (3H, s, OMe), 4.46 (1H, d, *J* = 12.2 Hz, O-CH_2_-), 4.54 (1H, d, *J* = 12.2 Hz, O-CH_2_-), 4.83–4.95 (2H, m, CH_2_=CH-), 5.11–5.27 (1H, m, CH-O), 5.45–5.50 (2H, m, -CH_2_-Ph), 5.63–5.76 (1H, m, -CH=CH_2_), 7.15–7.24 (2H, m, 2CH_Ph_), 7.28–7.39 (6H, m, 3CH_Ph_ + 2CHquinol + CHtriazole), 7.42 (1H, d, *J* = 3.7 Hz, CHquinol), 8.01 (1H, d, *J* = 9.5 Hz, CHquinol), 8.71 (1H, d, *J* = 4.5 Hz, CHquinol); ^13^C NMR (CDCl_3_, 125 MHz) δ 22.53–23.81 (CH_2_quinuc), 27.80 (CH_2_quinuc), 27.84 (CHquinuc), 40.05 (CH-CH=CH_2_), 43.06 (CH_2_quinuc-N), 54.19 (CH_2_Ph), 55.81 (OMe), 57.07 (CH_2_quinuc-N), 60.23 (CHquinuc-N), 62.70 (O-CH_2_-), 80.00–81.70 (CH-O), 101.43 (CHquinol), 114.33 (CH=CH_2_), 118.9–119.9 (CHquinol), 121.79 (CHquinol), 122.59 (CHtriazole), 128.07 (2CH_Ph_), 128.86 (1CH_Ph_), 129.20 (2CH_Ph_), 131.92 (CHquinol), 141.99 (CH=CH_2_), 147.70 (CHquinol), 127.52, 134.65, 144.51, 144.78, 145.15, 157.85 (6Cquater); MS *m*/*z* (*I*_rel_, %) 496.30 [M + H]^+^ (100); anal. calcd. for C_30_H_33_N_5_O_2_ (495.63): C, 72.70; H, 6.71; N, 14.13%; found: C, 72.85; H, 6.82; N, 14.01%.

*3-((4-(((R)-(6-methoxyquinolin-4-yl)((1S,2R,4S,5R)-5-vinylquinuclidin-2-yl)methoxy)methyl)-1H-1,2,3-triazol-1-yl)methyl)-5-phenylisoxazole* (**13**): light yellow powder; yield 59% (0.33 g), mp 76–77 °C; [α]d^25^ −75°; IR (KBr) ν 3131, 3070, 2925, 2861, 1619, 1591, 1574, 1507, 1467, 1452, 1431, 1359, 1340, 1320, 1240, 1226, 1130, 1099, 1074, 1047, 1028, 991, 947, 913, 854, 833, 820, 764, 717, 690, 640 cm^−1^; ^1^H NMR (CDCl_3_, 500 MHz) δ 1.33–1.49 (1H, m, CH_2_quinuc), 1.50–1.69 (3H, m, CH_2_quinuc), 1.69–1.76 (1H, m, CHquinuc), 2.16–2.25 (1H, m, CH-CH=CH_2_), 2.50–2.66 (2H, m, CH_2_quinuc-N), 3.01 (1H, dd, *J* = 13.5, 10.4 Hz, CH_2_quinuc-N), 3.06–3.17 (1H, m, CHquinuc-N), 3.22–3.37 (1H, m, CH_2_quinuc-N), 3.90 (3H, s, OMe), 4.49 (1H, d, *J* = 12.2 Hz, O-CH_2_-), 4.57 (1H, d, *J* = 12.2 Hz, O-CH_2_-), 4.82–4.96 (2H, m, CH_2_=CH-), 5.16–5.29 (1H, m, CH-O), 5.61–5.65 (2H, m, -CH_2_-Isx), 5.61–5.75 (1H, m, -CH=CH_2_), 6.48 (1H, s, CHisx), 7.30–7.39 (1H, m, CHquinol), 7.35 (1H, dd, *J* = 9.3, 2.3 Hz, CHquinol), 7.40–7.49 (4H, m, 1Hquinol + 3CH_Ph_), 7.61 (1H, s, CHtriazole), 7.69–7.76 (2H, m, CH_Ph_), 8.00 (1H, d, *J* = 9.6 Hz, CHquinol), 8.73 (1H, d, *J* = 4.5 Hz, CHquinol); ^13^C NMR (CDCl_3_, 125 MHz) δ 22.41–23.32 (CH_2_quinuc), 27.81 (CH_2_quinuc), 27.87 (CHquinuc), 40.03 (CH-CH=CH_2_), 43.18 (CH_2_quinuc-N), 45.69 (CH_2_-Isx), 55.86 (OMe), 57.10 (CH_2_quinuc-N), 60.20 (CHquinuc-N), 62.68 (O-CH_2_-), 80.30–81.60 (CH-O), 98.79 (CHisx), 101.40 (CHquinol), 114.42 (CH=CH_2_), 118.69–119.71 (CHquinol), 121.83 (CHquinol), 122.99 (CHtriazole), 125.98 (2CH_Ph_), 129.23 (2CH_Ph_), 130.90 (1CH_Ph_), 131.99 (CHquinol), 141.94 (CH=CH_2_), 147.74 (CHquinol), 126.77, 127.50, 144.30, 144.81, 145.64, 157.95, 159.20, 171.62 (8Cquater); MS m/z (I_rel_, %) 563.30 [M + H]^+^ (100); anal. calcd. for C_33_H_34_N_6_O_3_ (562.67): C, 70.44; H, 6.09; N, 14.94%; found: C, 70.57; H, 6.15; N, 14.81%.

*3-((1-(((R)-(6-methoxyquinolin-4-yl)((1S,2R,4S,5R)-5-vinylquinuclidin-2-yl)methoxy)methyl)-1H-1,2,3-triazol-4-yl)methyl)-5-(p-tolyl)isoxazole* (**14**): light yellow powder; yield 55% (0.32 g), mp 67–68 °C; [α]d^25^ −51; IR (KBr) ν 3134, 3073, 2924, 2862, 1619, 1590, 1570, 1508, 1470, 1455, 1431, 1360, 1342, 1319, 1240, 1226, 1184, 1170, 1129, 1112, 1101, 1075, 1046, 991, 948, 913, 855, 820, 788, 716, 640 cm^−1^; ^1^H NMR (CDCl_3_, 500 MHz) δ 1.35–1.48 (1H, m, CH_2_quinuc), 1.50–1.69 (3H, m, CH_2_quinuc), 1.69–1.76 м (1H, m, CHquinuc), 2.16–2.25 (1H, m, CH-CH=CH_2_), 2.36 (3H, s, Me), 2.50–2.66 (2H, m, CH_2_quinuc-N), 3.01 (1H, dd, *J* = 13.1, 10.4 Hz, CH_2_quinuc-N), 3.06–3.17 (1H, m, CHquinuc-N), 3.22–3.37 (1H, m, CH_2_quinuc-N), 3.89 (3H, s, OMe), 4.48 (1H, d, *J* = 12.2 Hz, O-CH_2_-), 4.56 (1H, d, *J* = 12.2 Hz, O-CH_2_-), 4.83–4.93 (2H, m, CH_2_=CH-), 5.16–5.29 (1H, m, CH-O), 5.61–5.63 (2H, m, -CH_2_-Isx), 5.64–5.74 (1H, m, -CH=CH_2_), 6.41 (1H, s, CHisx), 7.22 (2H, d, *J* = 8.0 Hz, CH_Tol_), 7.29–7.39 (1H, m, CHquinol), 7.34 (1H, dd, *J* = 9.3, 2.3 Hz, CHquinol), 7.42 (1H, d, *J* = 3.5 Hz, CHquinol), 7.61 (2H, d, *J* = 8.2 Hz, 2CH_Tol_), 7.61 (1H, s, CHtriazole), 8.00 (1H, d, *J* = 9.5 Hz, CHquinol), 8.71 (1H, d, *J* = 4.5 Hz, CHquinol); ^13^C NMR (CDCl_3_, 125 MHz) δ 21.60 (CH_3_), 22.08–23.33 (CH_2_quinuc), 27.74 (CH_2_quinuc), 27.81 (CHquinuc), 39.96 (CH-CH=CH_2_), 43.11 (CH_2_quinuc-N), 45.66 (CH_2_-Isx), 55.82 (OMe), 57.01 (CH_2_quinuc-N), 60.16 (CHquinuc-N), 62.63 (O-CH_2_-), 80.00–81.80 (CH-O), 98.13 (CHisx), 101.36 (CHquinol), 114.38 (CH=CH_2_), 118.54–119.73 (CHquinol), 121.80 (CHquinol), 122.97 (CHtriazole), 125.87 (2CH_Ph_), 129.86 (2CH_Ph_), 131.94 (CHquinol), 141.87 (CH=CH_2_), 147.70 (CHquinol), 124.03, 127.46, 141.26, 144.25, 144.77, 145.54, 157.90, 159.10, 171.75 (9Cquater); MS *m*/*z* (I_rel_, %) 577.30 [M + H]^+^ (100); anal. calcd. for C_33_H_34_N_6_O_3_ (576.70): C, 70.81; H, 6.29; N, 14.57%; found: C, 70.81; H, 6.29; N, 14.57%.

*4,5-dichloro-3-((4-(((R)-(6-methoxyquinolin-4-yl)((1S,2R,4S,5R)-5-vinylquinuclidin-2-yl)methoxy)methyl)-1H-1,2,3-triazol-1-yl)methyl)isothiazole (***15***):* white powder; yield 56% (0.32 g), mp 68–69 °C; [α]d^25^ −71°; IR (KBr) ν 3133, 3071, 3033, 2931, 2862, 1619, 1589, 1573, 1508, 1472, 1454, 1431, 1377, 1360, 1322, 1301, 1240, 1226, 1171, 1131, 1091, 1076, 1045, 1029, 977, 915, 856, 831, 820, 809, 789, 716, 640; ^1^H NMR (CDCl_3_, 500 MHz) δ 1.40–1.50 (1H, m, CH_2_quinuc), 1.50–1.72 (3H, m, CH_2_quinuc), 1.72–1.80 (1H, m, CHquinuc), 2.19–2.29 (1H, m, CH-CH=CH_2_), 2.53–2.69 (2H, m, CH_2_quinuc-N), 3.04 (1H, dd, *J* = 13.4, 10.4 Hz, CH_2_quinuc-N), 3.09–3.18 (1H, m, CHquinuc-N), 3.26–3.41 (1H, m, CH_2_quinuc-N), 3.92 (3H, s, OMe), 4.50 (1H, d, *J* = 12.2 Hz, O-CH_2_-), 4.60 (1H, d, *J* = 12.2 Hz, O-CH_2_-), 4.83–4.96 (2H, m, CH_2_=CH-), 5.22–5.36 (1H, m, CH-O), 5.60–5.65 (2H, m, -CH_2_-Isoth), 5.65–5.74 (1H, m, -CH=CH_2_), 7.31–7.41 (1H, m, CHquinol), 7.35 (1H, dd, *J* = 9.3, 2.3 Hz, CHquinol), 7.46 (1H, d, *J* = 3.6 Hz, CHquinol), 7.64 (1H, s, CHtriazole), 8.00 (1H, d, *J* = 9.4 Hz, CHquinol), 8.71 (1H, d, *J* = 4.4 Hz, CHquinol); ^13^C NMR (CDCl_3_, 125 MHz) δ 22.20–23.08 (CH_2_quinuc), 27.55 (CH_2_quinuc), 27.83 (CHquinuc), 39.82 (CH-CH=CH_2_), 43.09 (CH_2_quinuc-N), 49.59 (CH_2_–Isoth), 55.94 (OMe), 56.81 (CH_2_quinuc-N), 60.09 (CHquinuc-N), 62.63 (O-CH_2_-), 79.83–81.70 (CH-O), 101.38 (CHquinol), 114.60 (CH=CH_2_), 118.42–119.74 (CHquinol), 121.92 (CHquinol), 123.52 (CHtriazole), 131.94 (CHquinol), 141.64 (CH=CH_2_), 147.68 (CHquinol), 122.73, 127.47, 144.14, 144.78, 145.15, 149.73, 158.00, 159.31 (8Cquater); MS m/z (I_rel_, %) 571.20 [M] + (100); anal. calcd. for C_27_H_28_Cl_2_N_6_O_2_S (571.52): C, 56.74; H, 4.94; Cl, 12.41; N, 14.70; S, 5.61%; found: C, 56.85; H, 5.11; Cl, 12.31; N, 14.61; S, 5.52%.

*5-((4-(((R)-(6-methoxyquinolin-4-yl)((1S,2R,4S,5R)-5-vinylquinuclidin-2-yl)methoxy)methyl)-1H-1,2,3-triazol-1-yl)methyl)-3-(pyridin-2-yl)-1,2,4-oxadiazole* (**16**): oil; yield 15% (70 mg); [α]d^25^ −65°; IR (KBr) ν 3140, 3073, 2924, 2858, 1758 (C=Oest), 1619, 1590, 1508, 1472, 1456, 1434, 1363, 1240, 1225, 1048, 1026, 992, 913, 856, 831, 802, 716 cm^−1^; ^1^H NMR (CDCl_3_, 500 MHz) δ 1.44–1.62 (2H, m, CH2quinuc), 1.68–1.86 (3H, m, CH_2_quinuc + CHquinuc), 2.23–2.37 (1H, m, CH-CH=CH_2_), 2.56–2.77 (2H, m, CH_2_quinuc-N), 3.05–3.15 (1H, m, CH_2_quinuc-N), 3.15–3.20 (1H, m, CHquinuc-N), 3.34–3.49 (1H, m, CH_2_quinuc-N), 3.80 (3H, s, CO_2_Me), 3.92 (3H, s, OMe), 4.51 (1H, d, *J* = 12.2 Hz, O-CH_2_-), 4.67 (1H, d, *J* = 12.2 Hz, O-CH_2_-), 4.87–4.97 (2H, m, CH_2_=CH-), 5.18 (2H, m, -CH_2_-CO_2_Me), 5.34–5.56 (1H, m, CH-O), 5.58–5.76 (1H, m, -CH=CH_2_), 7.37 (1H, dd, *J* = 9.3, 2.3 Hz, CHquinol), 7.37–7.45 (1H, m, CHquinol), 7.50 (1H, d, *J* = 3.5 Hz, CHquinol), 7.69 (1H, s, CHtriazole), 8.03 (1H, d, *J* = 9.2 Hz, CHquinol), 8.75 (1H, d, *J* = 4.4 Hz, CHquinol); ^13^C NMR (CDCl_3_, 125 MHz) δ 27.18 (CH_2_quinuc), 27.75 (CHquinuc), 29.83 (CH_2_quinuc), 39.53 (CH-CH=CH_2_), 43.23 (CH_2_quinuc-N), 50.82 (CH_2_–CO_2_Me), 53.23 (CO_2_Me), 56.22 (OMe), 56.59 (CH_2_quinuc-N), 60.03 (CHquinuc-N), 62.47 (O-CH_2_-), 79.78–81.79 (CH-O), 101.32 (CHquinol), 114.98 (CH=CH_2_), 118.6–119.6 (CHquinol), 122.17 (CHquinol), 124.42 (CHtriazole), 131.96 (CHquinol), 141.17 (CH=CH_2_), 147.67 (CHquinol), 127.47, 143.71, 144.80, 144.82, 158.25, 166.90 (6Cquater); MS m/z (I_rel_, %) 478.30 [M + H]^+^ (100); anal. calcd. for C_26_H_31_N_5_O_4_ (477.57): C, 65.39; H, 6.54; N, 14.67%; found: C, 65.70; H, 6.72; N, 14.55%.

## 4. Conclusions

Convenient approaches for the preparation of polyazaheterocyclic quinine derivatives via acylation and azide–alkyne cycloaddition reactions have been developed. The process of 1,2,4-oxadiazole ring cleavage was noted to occur under CuAAC reaction conditions.

According to the bioassay data, almost all of the tested substances did not show high effectiveness. As for antibacterial properties, the (p-tolyl)isoxazolyltriazole derivative of quinine **14** showed the best results, demonstrating moderate activity against all test strains of microorganisms and exerting antibacterial activity comparable to that of the reference drug against *Bacillus subtilis* at a concentration of 21.6 μM. The anabasine–quinine hybrid **3**, cinchophen (**4**, **5**) and isothiazolyltriazole (**15**) derivatives of quinine showed the greatest analgesic effect among the tested compounds, causing a decrease in the amount of vinegar writhing in mice by 41.2–45.6% at a dosage of 25 mg/kg. Most of the tested compounds showed good cytotoxic activity in a survival test of *Artemia salina* marine crustacean larvae (LC_50_ in the range of 77.9–102.1 μg/mL).

## Figures and Tables

**Table 1:** Minimum inhibitory concentration (MIC, μM) of the tested compounds.

Compound	*Staphylococcus* *aureus* *ATCC 6538*	*Bacillus* *subtilis* *ATCC 6633*	*Escherichia* *coli* *ATCC 25922*	*Candida* *albicans* *ATCC 10231*
**1**	31.1	124.7	62.3	–
**2**	27.8	−	111.2	−
**3**	23.7	94.9	23.7	−
**4**	45.0	–	90.0	–
**5**	29.7	29.7	−	59.5
**12**	25.2	50.4	50.4	100.8
**13**	44.4	88.8	88.8	−
**14**	21.6	21.6	43.3	86.7
**15**	43.7	43.7	87.4	*−*
**16**	53.4	104.7	104.7	−
Ceftriaxone	11.3	22.5	11.3	–
Nystatin	–	–	–	13.4

Sign “–” means that the compound was not active in this experiment.

**Table 2:** Analgesic activity of test compounds.

Compound	Dose, mg/kg	Writhing Number	Decrease in the Vinegar Writhing Number (%)
control	-	106.2 ± 11.2	100
sodium diclofenac	8	49.5 ± 10.7	53.3
quinine	25	75.4 ± 9.5	29.0
**1**	25	67.7 ± 6.8	35.0
**2**	25	72.8 ± 9.6	30.0
**3**	25	62.2 ± 11.2	41.4
**4**	25	56.6 ± 9.4	45.6
**5**	25	58.9 ± 10.5	43.4
**12**	25	85.5 ± 10.6	18.3
**13**	25	73.8 ± 11.0	27.8
**14**	25	73.2 ± 12.4	30.1
**15**	25	60.2 ± 9.1	41.2

**Table 3:** Cytotoxic activity of quinine derivatives.

Compound	LC_50_, μg/mL
quinine	97.1
**1**	94.6
**2**	>1000 μg/mL
**3**	89.5
**4**	>1000 μg/mL
**5**	>1000 μg/mL
**12**	77.9
**13**	86.5
**14**	102.1
**15**	91.4
**16**	82.2
Dactinomycin	44.7

## Data Availability

The data presented in this study are available in the article or Supplementary Material.

## Supplementary Materials

The following supporting information can be downloaded at https://www.mdpi.com/article/10.3390/molecules30153301/s1, NMR, MS, IR spectra, instrumentation and biotesting methodology. References [18,19,20,21,22] are cited in the supplementary materials.
