# Supplementary material for: Synthesis and Biological Activity of Novel Polyazaheterocyclic Derivatives of Quinine
Source: Molecules. 2025 Aug 7;30(15):3301. doi: 10.3390/molecules30153301 (PMC12348970; doi:10.3390/molecules30153301)
Supplement: Supplementary file 1 [file molecules-30-03301-s001.zip › molecules-3745735-supplementary.pdf]

Supplementary Materials for the paper

Synthesis and Biological Activity of Novel Polyazaheterocyclic Derivatives of Quinine

Gulim K. Mukusheva[1], Nurizat N.Toigambekova[1], Roza B. Seidakhmetova[2], Roza I. Jalmakhanbetova[3], Mukhlissa N. Babakhanova[3], Oralgazy A. Nurkenov[4], Ekaterina A. Akishina[5,*], Evgenij A. Dikusar[5], Irina A. Kolesnik[5], Hongwei Zhou[6] and Vladimir I. Potkin[5]

1 *Chemistry Faculty, Karaganda Buketov University, 100024 Karaganda, Kazakhstan; mukushevagulim5@gmail.com (G.K.M.); nukonti92@mail.ru (N.N.T.).*

2 *Department of Clinical Pharmacology and Evidence-Based Medicine, Karaganda Medical University, Karaganda 100024, Kazakhstan; rozabat@mail.ru (R.B.S.).*

3 *Faculty of Natural Sciences, L.N. Gumilyov Eurasian National University, Astana 010000, Kazakhstan; rozadichem@mail.ru (R.I.J); muhlisa170920032003@gmail.com (M.N.B.).*

4 *Institute of Organic Synthesis and Coal Chemistry of the Republic of Kazakhstan, 100008 Karaganda, Kazakhstan; nurkenov_oral@mail.ru (O.A.N.).*

5 *Institute of Physical Organic Chemistry, National Academy of Sciences of Belarus, 220072 Minsk, Belarus; che.semenovaea@mail.ru (E.A.A.); dikusar@ifoch.bas-net.by (E.A.D.); irynakolesnik93@gmail.com (I.A.K.); potkin@ifoch.bas-net.by (V.I.P.).*

6 *College of Biological, Chemical Sciences and Engineering, Jiaxing University, Jiaxing 314001, China; zhouhw@zju.edu.cn (H.Z.)*

* *Correspondence: che.semenovaea@mail.ru; Tel.: +375-(17)-3971600*

TABLE OF CONTENTS

1. Materials and Methods……………………………………S2

2. Copy of the IR spectra……………………………………S5

3. Copy of the NMR spectra and mass-spectrometry data……………………………………S27

1. Materials and Methods

General Chemistry Section

IR spectra were registered on a Thermo Nicolet Protege 460 Fourier transform spectrometer in KBr pellets.

^{1}H and ^{13}C NMR spectra were acquired on a Bruker Avance 500 spectrometer (500 and 125 MHz, respectively) in DMSO-*d6* and $CDCl_3$. The residual solvent signals (DMSO-*d6*, δH 2.5, δC 40.1 ppm; $CDCl_3$, δH 7.26, δC 77.2 ppm) were used as the internal standard. The assignment of signals in the ^{13}C NMR spectra was performed using the DEPT technique.

Liquid chromatography–mass spectrometry spectra were recorded on an Agilent 1200 LC-MS system, with an Agilent 6410 Triple Quad Mass Selective Detector with electrospray ionization in the positive ion registration mode (MS2 scanning mode). An Agilent ZORBAX Eclipse XDB-C18 (4.6 × 50 mm, 1.8 μm) column was used. The mobile phase was MeCN–H_2O + 0.05% HCO_2H, with gradient elution from 40 to 90% MeCN in 10 min. A flow rate of 0.5 mL/min was used.

Elemental analysis was performed on a Vario MICRO cube CHNS-analyzer. The halogen content was determined by classical microanalysis, using a modified Pregl's method. Melting points were determined on a Kofler bench.

The optical activity of the compounds was measured on a polarimeter MCP100 (Anton Paar, Graz, Austria).

The reagents and solvents used were of analytical grade, with the content of the main component being more than 99.5%. Triethylamine (99.5%, EKOS-1) did not require additional purification. Dichloromethane (99.8%, EKOS-1) was preliminarily kept for 1 day over $CaCl_2$, to remove 0.5% of the ethanol used for stabilizing dichloromethane. The diethyl ether (99.5%, Kuzbassorghim) was distilled from lithium aluminum hydride.

(–)-Anabasine (colorless viscous liquid, turning yellow in air and in light; bp 276 °C at 760 mmHg, 104–105°C at 2 mmHg;d_{20} 1.0455, n_D 1.5430, $[\alpha]_D^{20}$ –82°) was isolated from anabasine hydrochloride (commercial product of Shymkentbiopharm, Kazakhstan) as an individual isomer.

Cytotoxicity Assays

The cytotoxicity of the samples was evaluated in a survival test of marine crustacean larvae *Artemia salina* (Leach). The experiments were conducted on 2-day-old larvae under cultivation conditions in vitro. Larvae were grown by adding the marine crustacean eggs of *Artemia salina* into artificial sea water and incubating them for 48 h at 37°C. A test portion of each sample was dissolved in ethanol (2 ml). Then, 500 μl (3 parallels), 50 μl (3 parallels) and 5 μl (3 parallels) were taken from this solution.

After ethanol evaporation, 5 ml of artificial sea water was added to each bottle. Thus, if the initial sample weight was 2 mg, the final sample concentrations were 100 μg/mL, 10 μg/mL and 1 μg/mL, respectively, of each concentration in triplicate. Ten marine crustacean larvae of *Artemia salina* (2 days old) were placed in each sample bottle using a Pasteur pipette.

After 24 hours, the surviving and dead larvae were counted. Then, using the obtained data on the upper and lower toxic limits, the half-toxic dose of the sample was calculated. Control—DMSO in equivolume quantities.

The test was performed using ready-made samples as well as a comparison drug, dactinomycin (actinomycin D), which had antitumor (cytotoxic) activity (producer: Sigma Aldrich, St. Louis, MO, USA). Lethal concentrations of these compounds, leading to 50% death of the shrimp (LC_{50}), and 95% confidence

intervals were determined based on 24 h calculations with probit analysis, and LC_{50} values were obtained with a 95% confidence interval [1].

Antimicrobial Activity in vitro

The antimicrobial activity of the samples was studied on the reference test microorganisms recommended by the State Pharmacopoeia of the Republic of Kazakhstan—facultative anaerobic Gram-positive cocci *Staphylococcus aureus ATCC 6538*, aerobic Gram-positive spore-forming rods *Bacillus subtilis ATCC 6633*, Gram-negative facultative anaerobe rods *Escherichia coli ATCC 25922* and yeast fungus *Candida albicans ATCC 10231*—using the method of random dilutions to determine the minimum inhibitory concentration (MIC) [2, 3]. The test strains of microorganisms used in the study were obtained from the American Type Culture Collection [4].

For the serial dilution method, suspensions of test strains at a concentration of 10^6 CFU/mL were used. A suspension of test strains of microorganisms was prepared from daily cultures grown on slant agar at a temperature of 37 °C for 24 h, and for the yeast fungus *Candida albicans* at 30 °C for 48 h. The antimicrobial activity of the samples was studied at dilutions in the range of 1.56–50 µg/mL. An amount of 0.1 mL of microbial suspension at a concentration of 10^6 CFU/mL was added to each test tube, with a working dilution of each test sample. The procedure was repeated for all test cultures. A suspension of microbes with a nutrient medium without a sample was placed in control tubes. The mixture was incubated in a thermostat for 24–48 h, depending on the class of the microorganism. Following this, upon visually determining the presence of turbidity in each of the tubes, we chose the one that contained a clear suspension and the lowest concentration of the antimicrobial agent. This concentration was taken as the minimum bactericidal concentration. All experiments were carried out three times.

The antibacterial drug ceftriaxone and the antifungal drug nystatin were used as reference drugs.

Analgesic activity in vivo

The experimental part was carried out in accordance with the "Rules of the European Convention for the Protection of Vertebrate Animals used for Experimental and Other Scientific Purposes" and in accordance with the requirements for the study of new pharmacological substances [5].

The analgesic effect of the synthesized compounds was eastablished using chemical stimulus on outbred white mice weighing in the range of 20 to 25 g. The experimental animals were kept in standard vivarium conditions on a normal diet. Five groups containing six animals each were formed (control, reference drug "diclofenac sodium", and three novel substances).

The analgesic effect of the samples was evaluated in the chemical irritation test of the peritoneum (test "vinegar cramps"). The abdominal constriction test is a visceral inflammatory pain model (acute peritonitis model). When visceral receptors are irritated with acetic acid, abdominal muscle contraction, hind limb extension and body elongation are observed [6]. A 0.75% solution of acetic acid was injected intraperitoneally at an amount of 0.1 mL per 10 g of animal weight. The potential pharmaceutically active substances were injected intragastrically at a dose of 25 mg/kg 30 min before the administration of the acetic acid. Immediately after the introduction of the stimulus, the latent time of the onset of the pain reaction "writhing" was recorded, and the writhings were counted for 30 min. The analgesic effect of compounds was determined by the ability to reduce the number of "writhings" counted for 10, 15, 20 and 30 min, compared with the corresponding indicators in the control animal group. The model drug was the non-steroidal anti-inflammatory drug diclofenac sodium, which was tested at an effective dose of 8 mg/kg (ED_{50} = 8 mg/kg). Control animals received the equivalent volume of starchy mucus. Analgesic activity was

expressed as a percentage reduction in the number of acetic writhings in experimental rats compared to controls.

Statistical processing was carried out by parametric statistical methods with the calculation of the arithmetic mean and standard error. Differences were considered significant at the achieved significance level of $p < 0.05$.

References

1. Meyer, B.N.; Ferrigni, N.R.; Putnam, J.E.; Jacobsen, L.B.; Nicholsand, D.E.; McLaughlin, J.L. Brine Shrimp: A Convenient General Bioassay for Active Plant Constituents. Planta Medica 1982, 45, 31–34.
2. State Pharmacopoeia of the Republic of Kazakhstan; Publishing House "Zhibek Zholy": Almaty, Kazakhstan, 2015; Volume I, 720p.
3. Guidelines for Conducting Preclinical Studies of Drugs. Mironov, A.N., Ed.; GRIF-K: Moscow, Russia, 2012; Part 1; p. 206.
4. Badshah, S.L.; Ullah, A. New developments in non quinolone-based antibiotics for the inhibition of bacterial gyrase and topoisomerase IV. Eur. J. Med. Chem. 2018, 152, 393–400.
5. Council, N.R. Guide for the Care and Use of Laboratory Animals, 8th ed.; National Academies Press: Washington, DC, USA, 2011; 220p
6. Réus, G.Z.; Stringari, R.B.; de Souza, B.; Petronilho, F.; Dal-Pizzol, F.; Hallak, J.E.; Zuardi, A.W.; Crippa, J.A.; Quevedo, J. Harmine and Imipramine Promote Antioxidant Activities in Prefrontal Cortex and Hippocampus. Oxid. Med. Cell. Longev. 2010, 3, 325–331.

2. Copy of the IR spectra

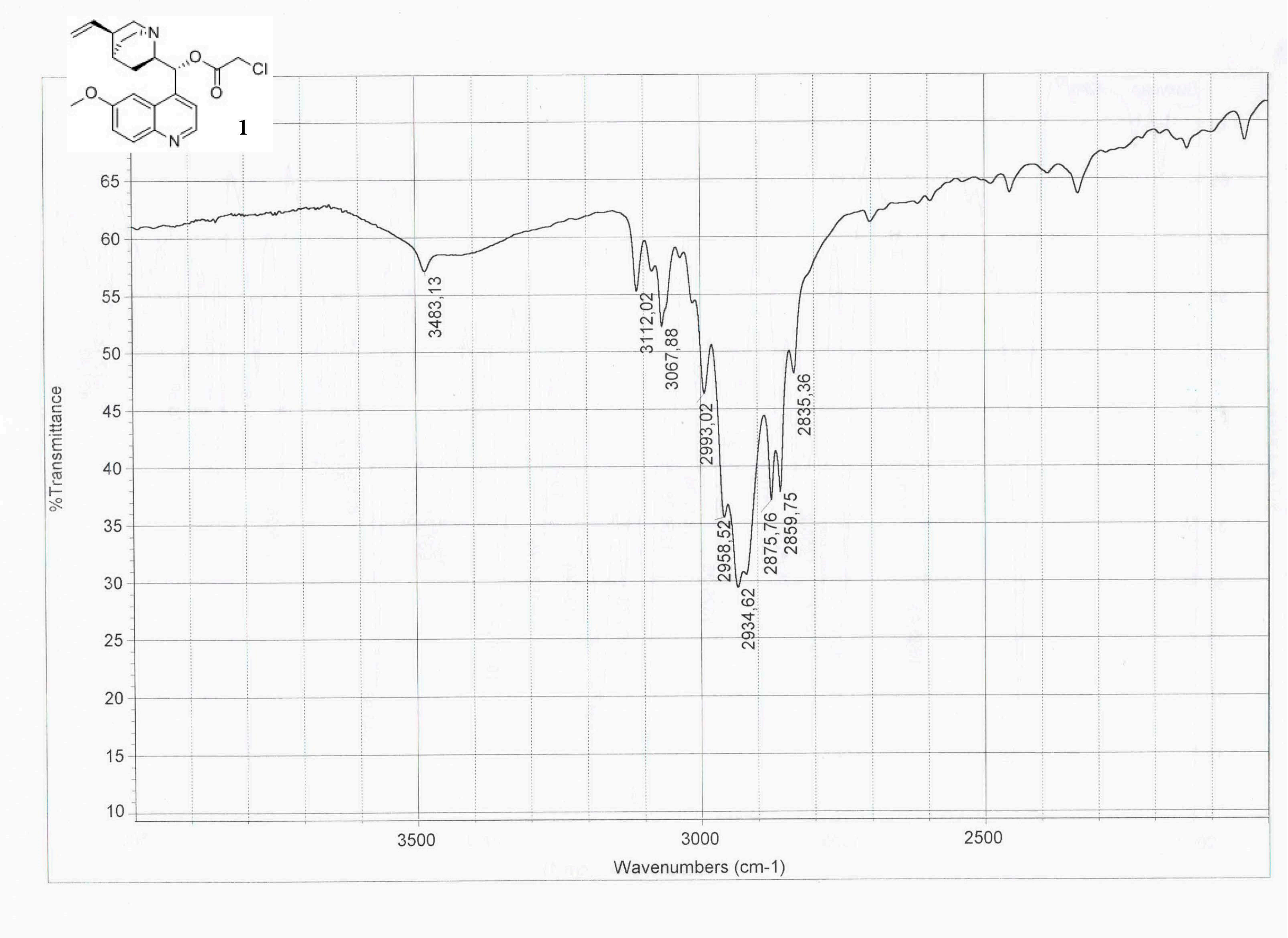

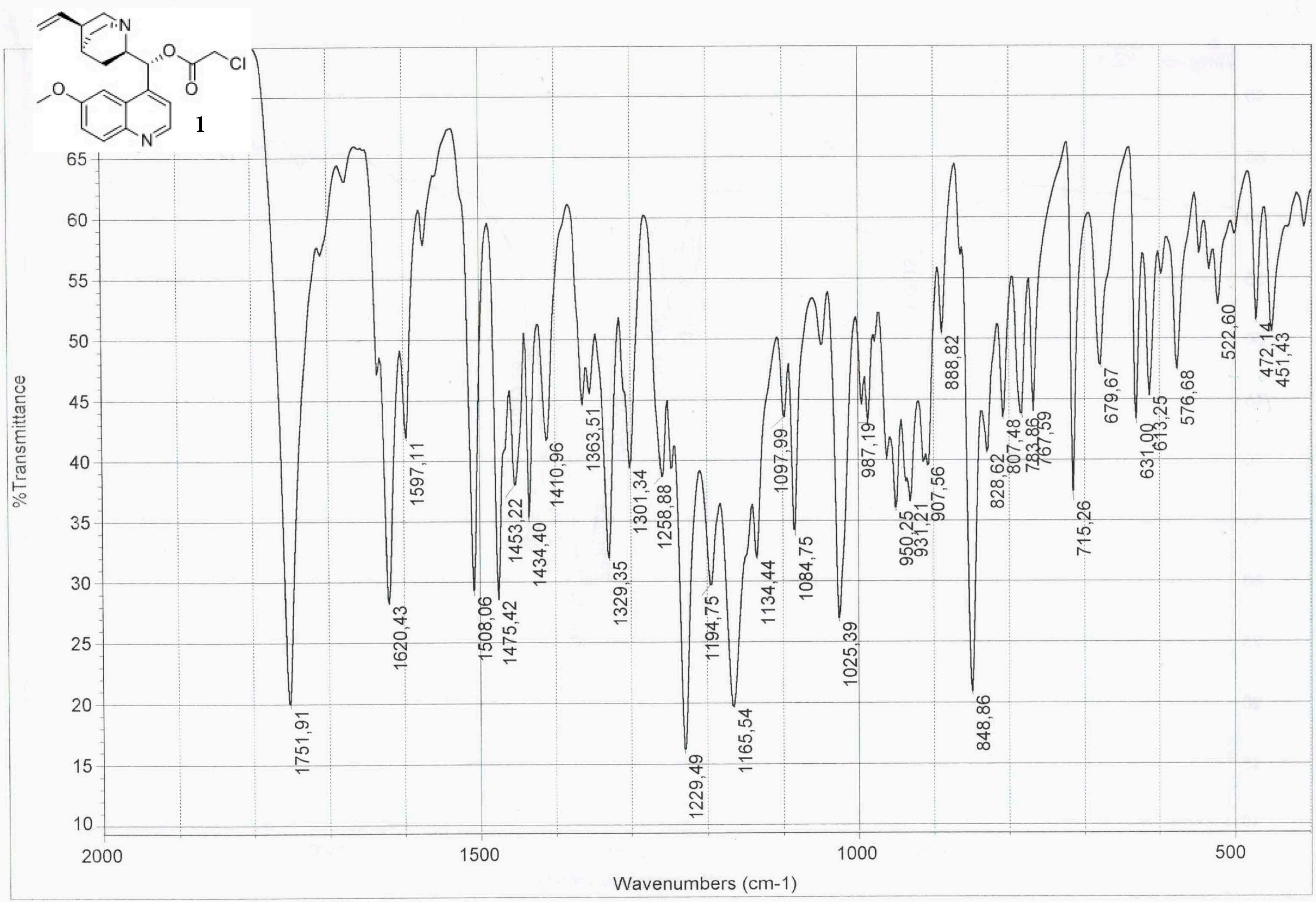
1
%Transmittance
Wavenumbers (cm-1)
65
60
55
50
45
40
35
30
25
20
15
10
2000
1500
1000
500
1751,91
1620,43
1597,11
1508,06
1475,42
1453,22
1434,40
1410,96
1363,51
1329,35
1301,34
1258,88
1229,49
1194,75
1165,54
1134,44
1097,99
1084,75
1025,39
987,19
950,25
931,21
907,56
888,82
848,86
828,62
807,48
783,86
767,59
715,26
679,67
631,00
613,25
576,68
522,60
472,14
451,43

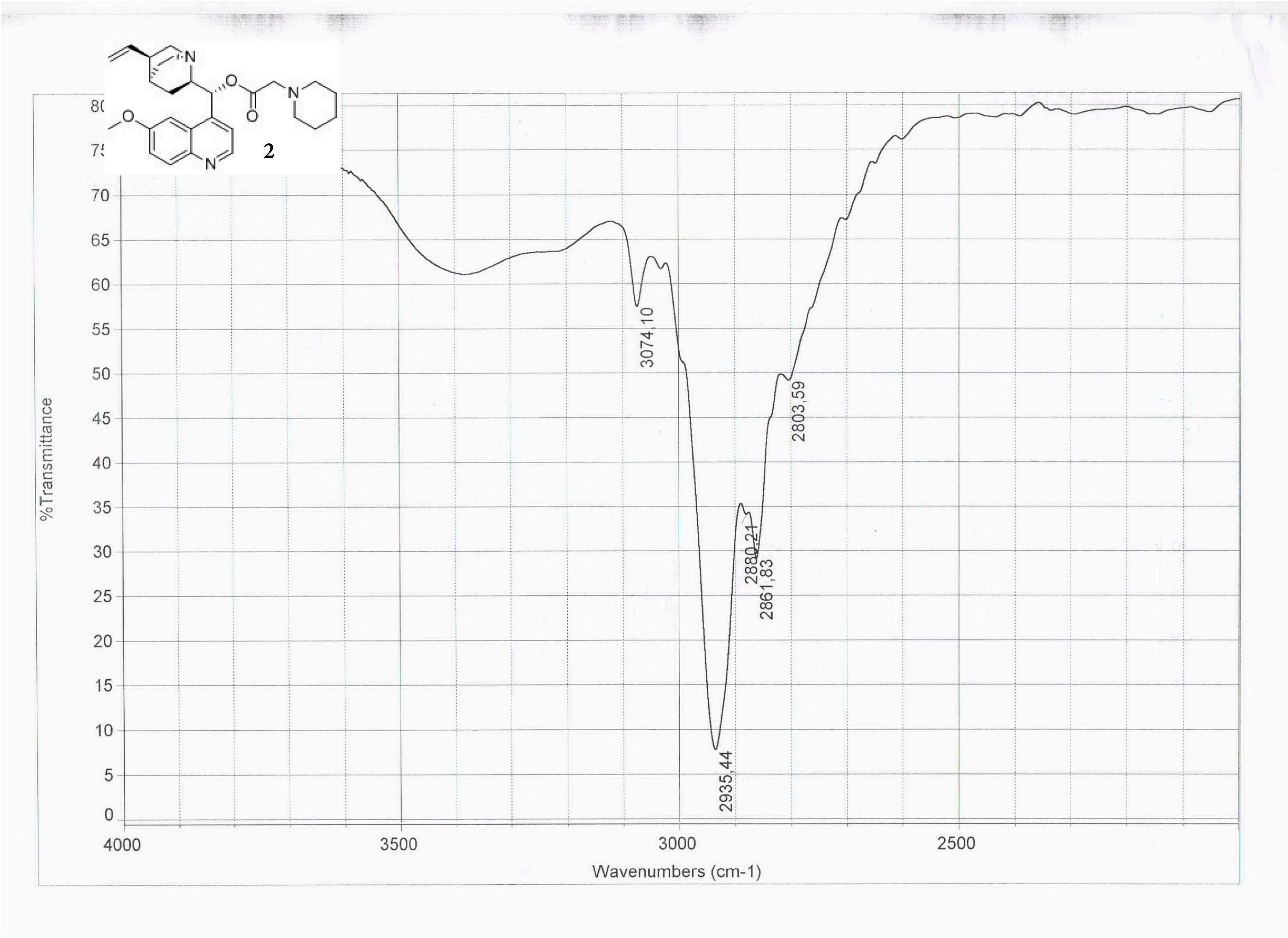
2
%Transmittance
Wavenumbers (cm-1)
3074,10
2935,44
2880,21
2861,83
2803,59
4000
3500
3000
2500

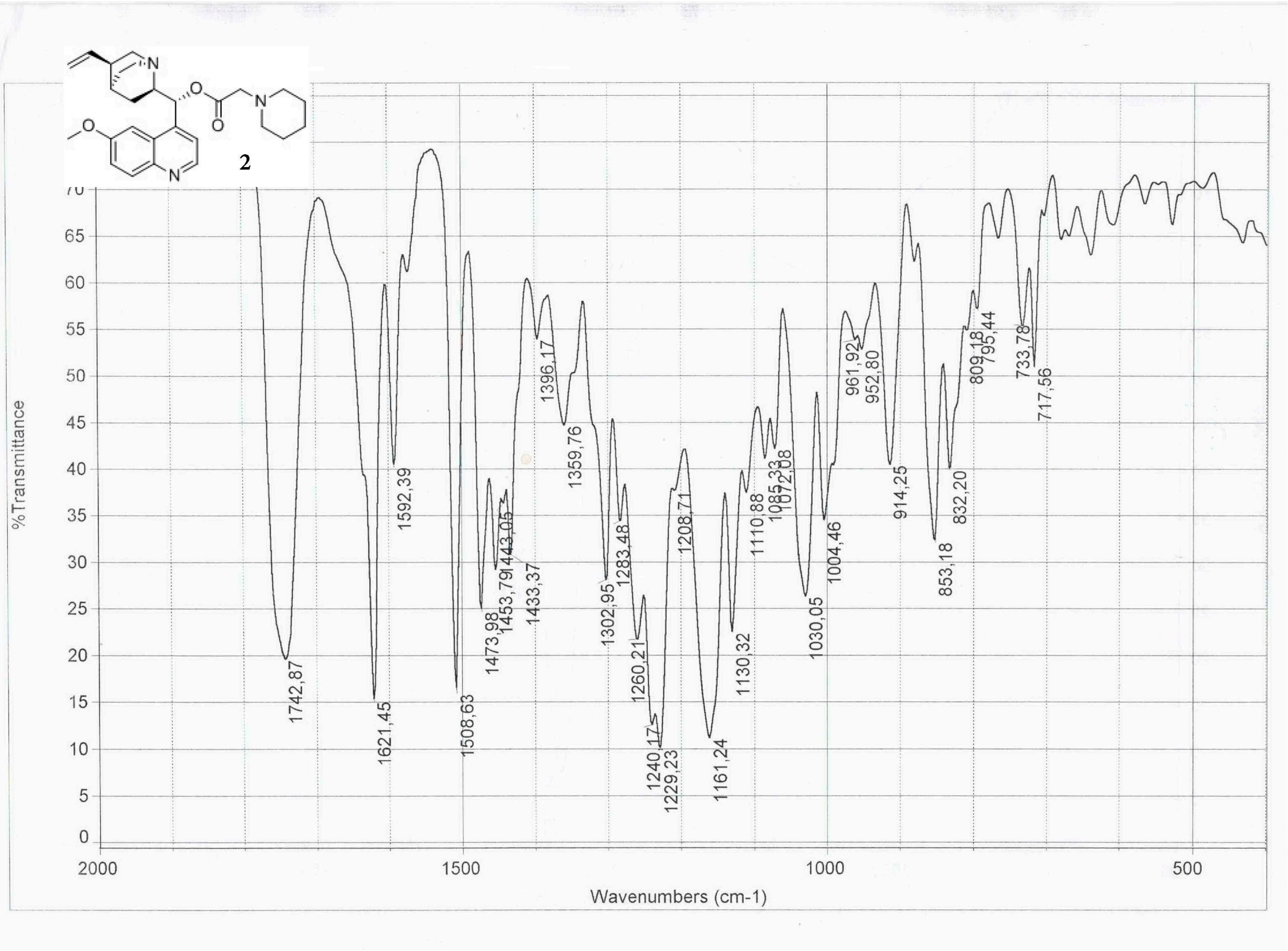
2
%Transmittance
Wavenumbers (cm-1)
2000
1500
1000
500
0
5
10
15
20
25
30
35
40
45
50
55
60
65
1742,87
1621,45
1592,39
1508,63
1473,98
1453,79
1443,05
1433,37
1396,17
1359,76
1302,95
1283,48
1260,21
1240,17
1229,23
1208,71
1161,24
1130,32
1110,88
1085,33
1072,08
1030,05
1004,46
961,92
952,80
914,25
853,18
832,20
809,18
795,44
733,78
717,56

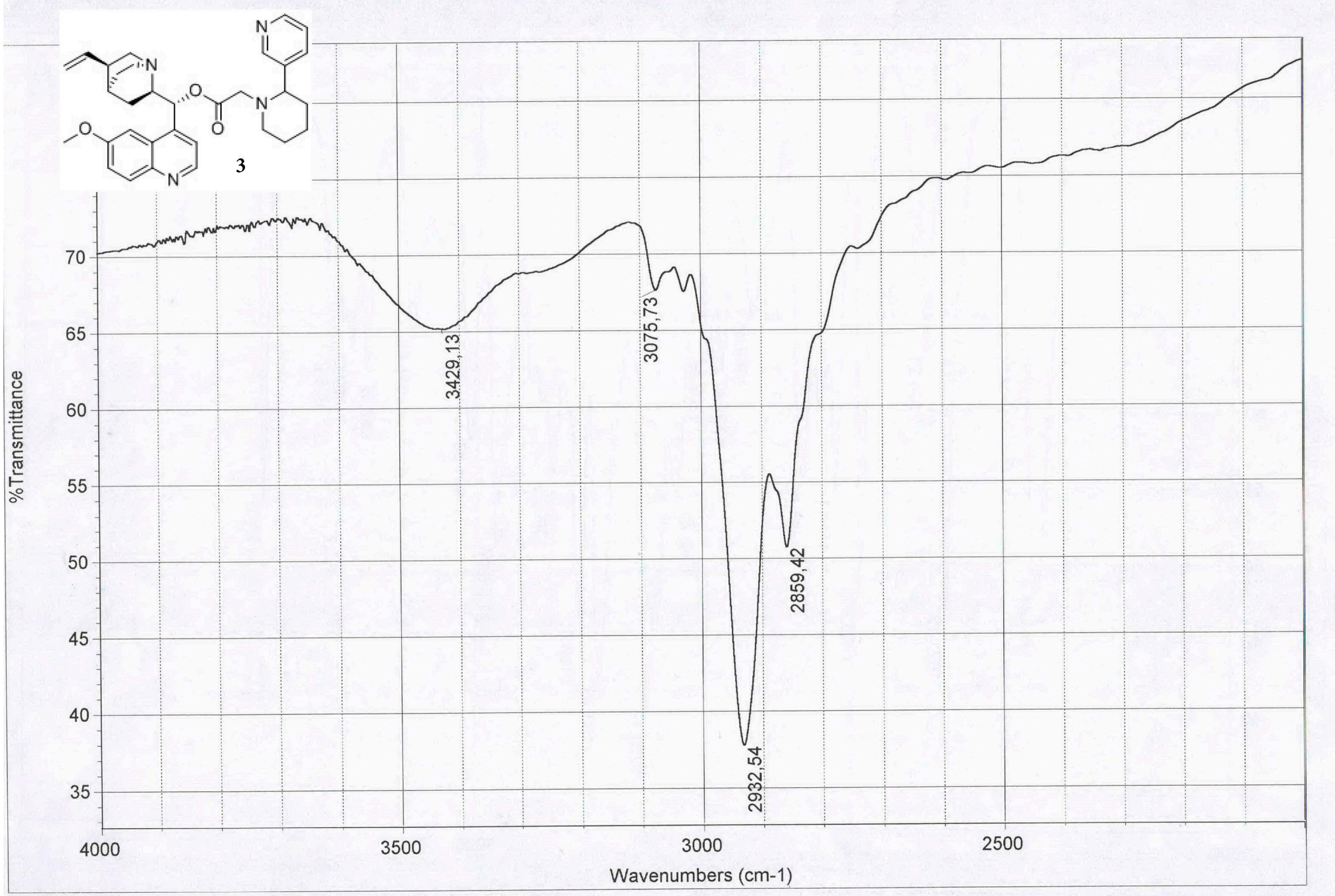

3
%Transmittance
Wavenumbers (cm-1)
3429,13
3075,73
2932,54
2859,42

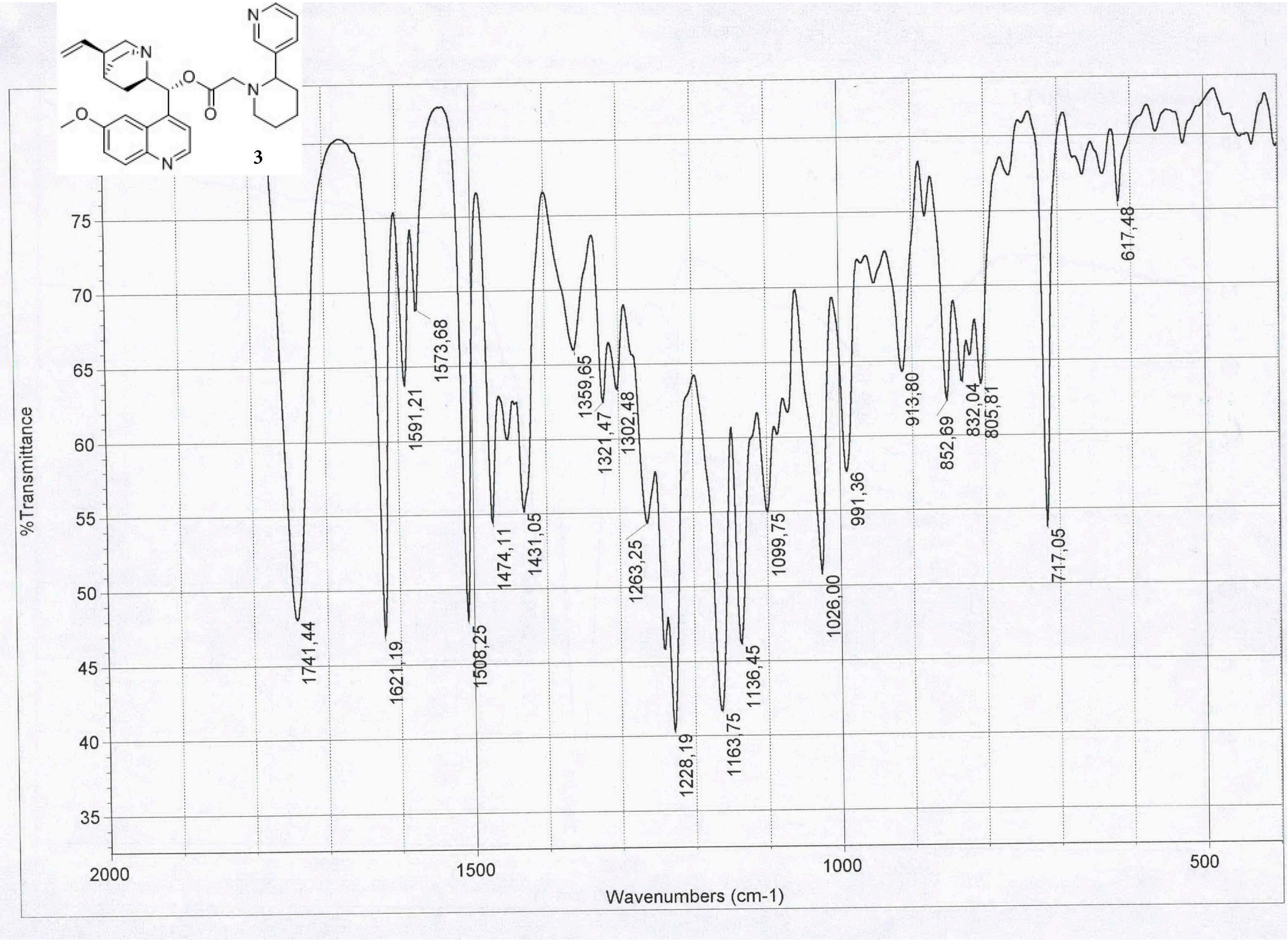
3
N
O
%Transmittance
75
70
65
60
55
50
45
40
35
2000
1500
1000
500
Wavenumbers (cm-1)
1741,44
1621,19
1591,21
1573,68
1508,25
1474,11
1431,05
1359,65
1321,47
1302,48
1263,25
1228,19
1163,75
1136,45
1099,75
1026,00
991,36
913,80
852,69
832,04
805,81
717,05
617,48

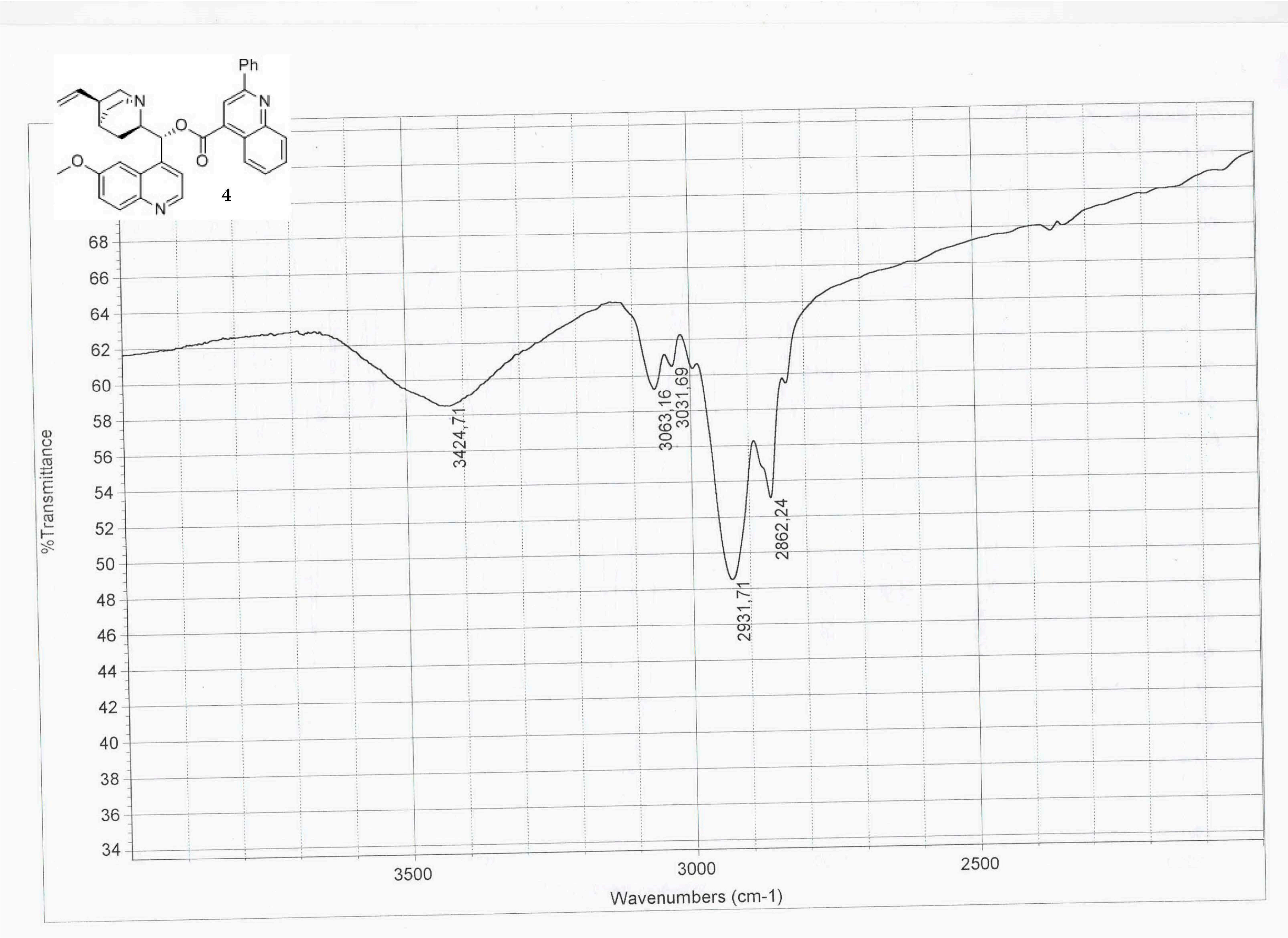
Ph
N
O
O
N
N
O
4
%Transmittance
Wavenumbers (cm-1)
3424,71
3063,16
3031,69
2931,71
2862,24
68
66
64
62
60
58
56
54
52
50
48
46
44
42
40
38
36
34
3500
3000
2500

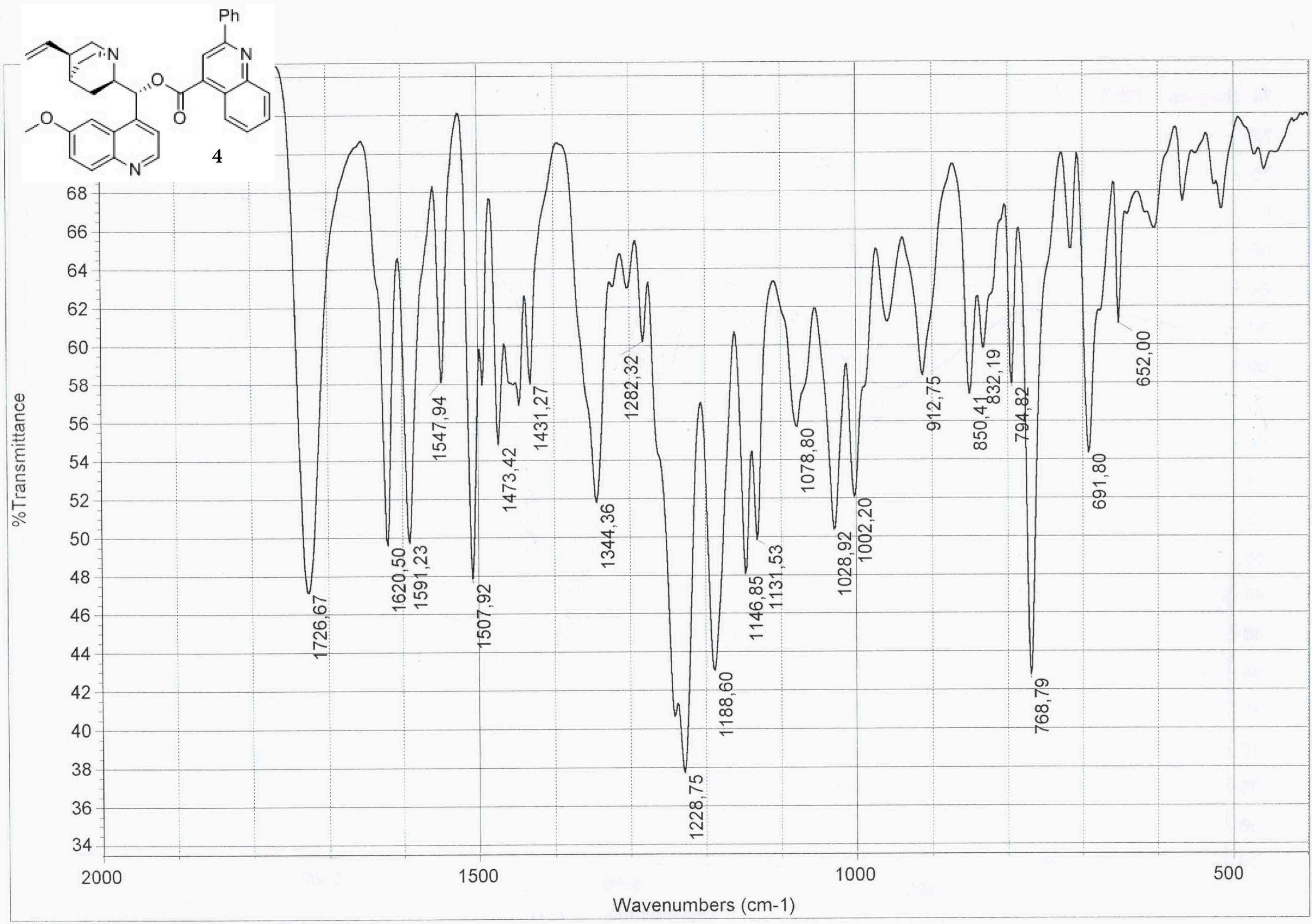
Ph
N
O
O
N
N
4
%Transmittance
Wavenumbers (cm-1)
1726,67
1620,50
1591,23
1547,94
1507,92
1473,42
1431,27
1344,36
1282,32
1228,75
1188,60
1146,85
1131,53
1078,80
1028,92
1002,20
912,75
850,41
832,19
794,82
768,79
691,80
652,00

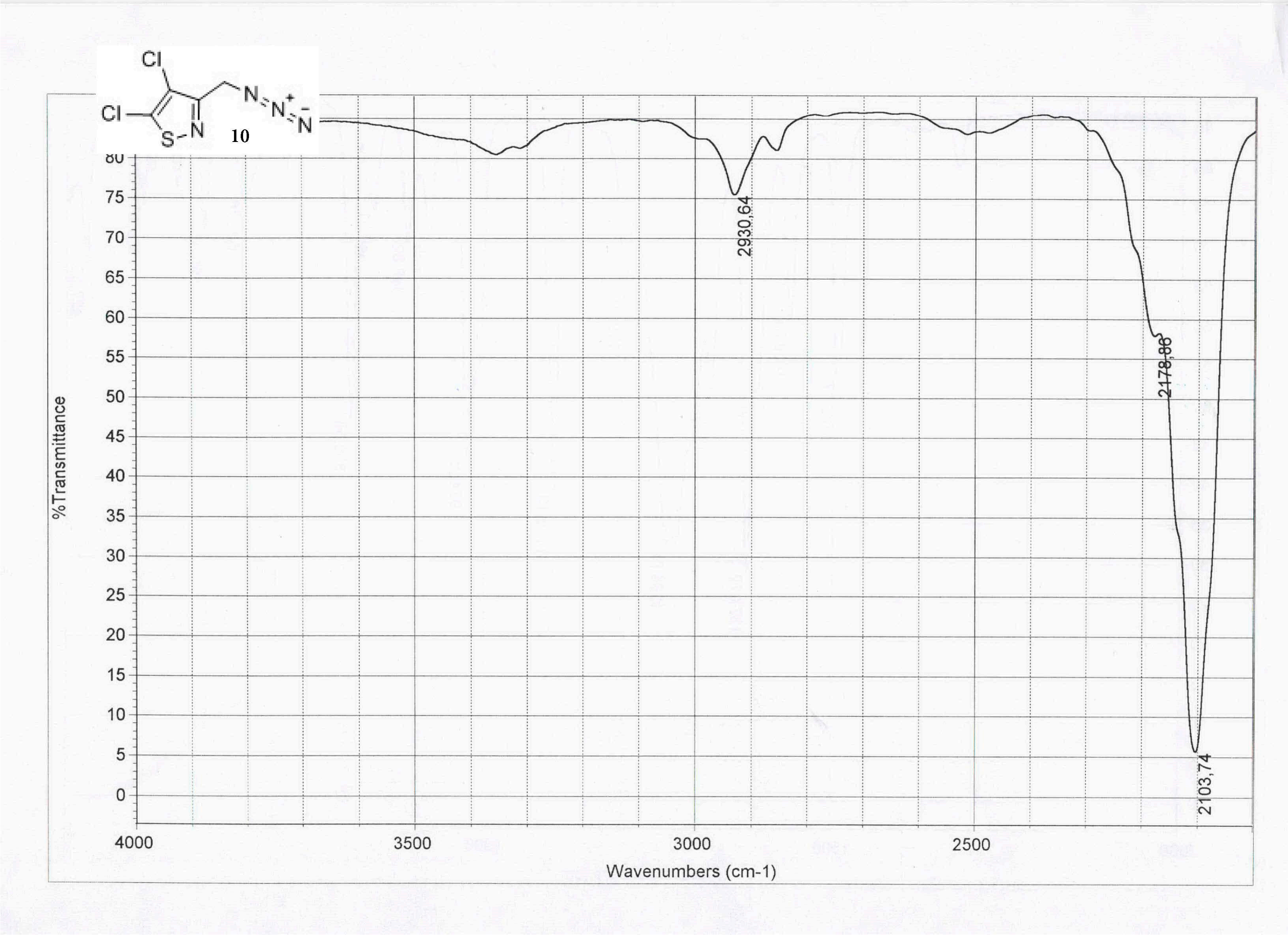
10
%Transmittance
Wavenumbers (cm-1)
2930,64
2178,86
2103,74
4000
3500
3000
2500

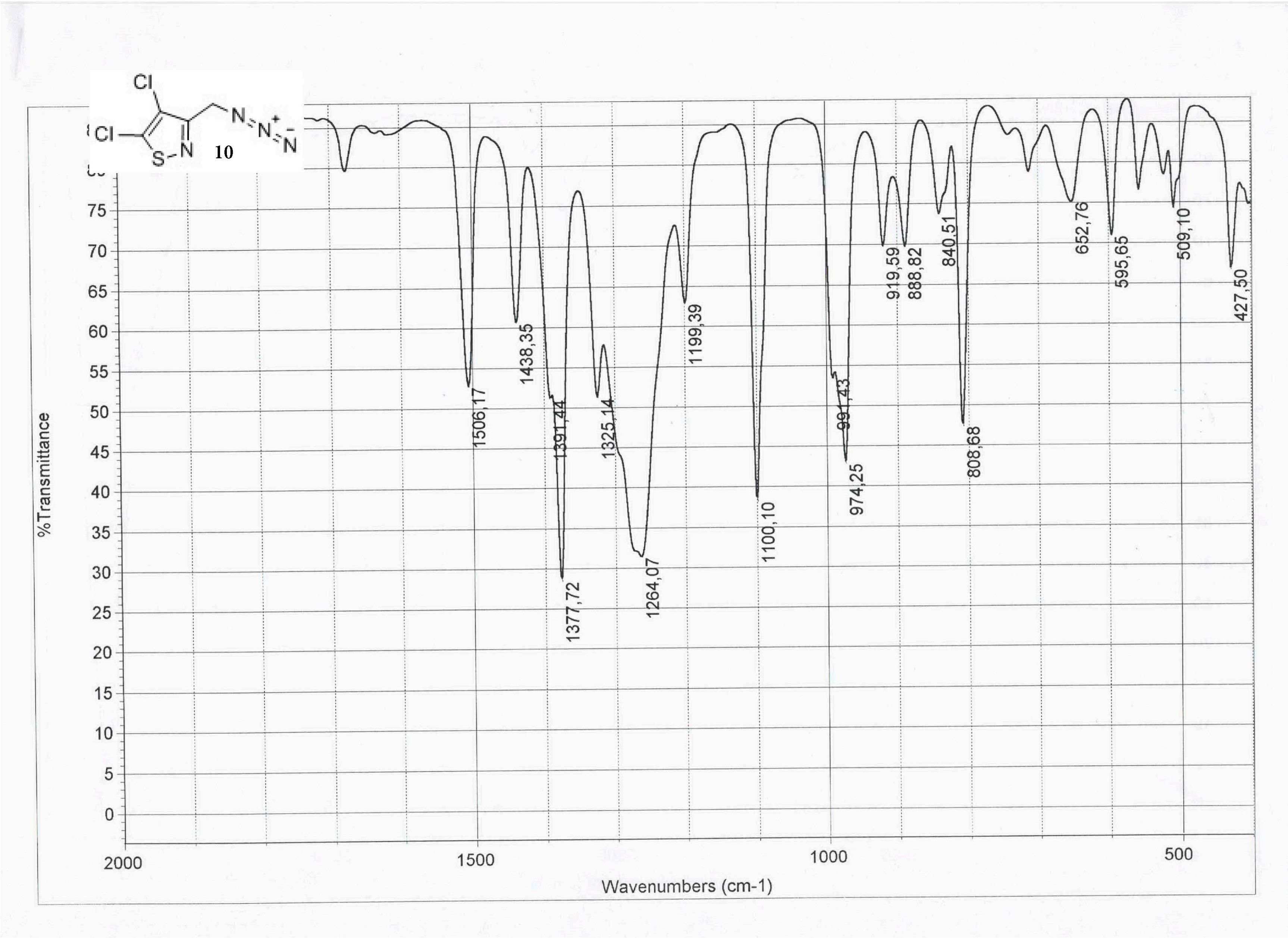
10
%Transmittance
Wavenumbers (cm-1)
2000
1500
1000
500
1506,17
1438,35
1391,44
1377,72
1325,14
1264,07
1199,39
1100,10
991,43
974,25
919,59
888,82
840,51
808,68
652,76
595,65
509,10
427,50

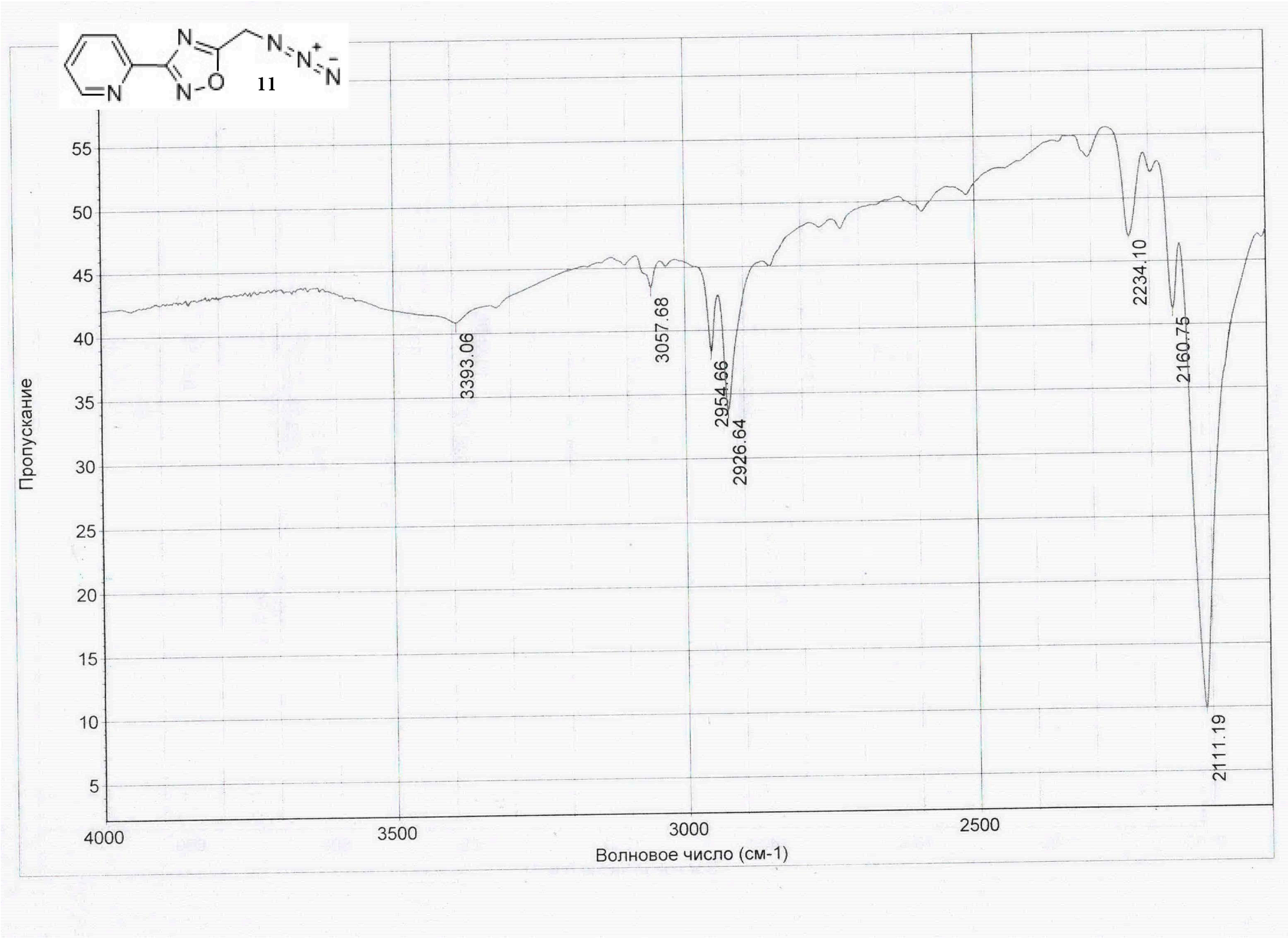
11
Пропускание
55
50
45
40
35
30
25
20
15
10
5
3393.06
3057.68
2954.66
2926.64
2234.10
2160.75
2111.19
4000
3500
3000
2500
Волновое число (см-1)

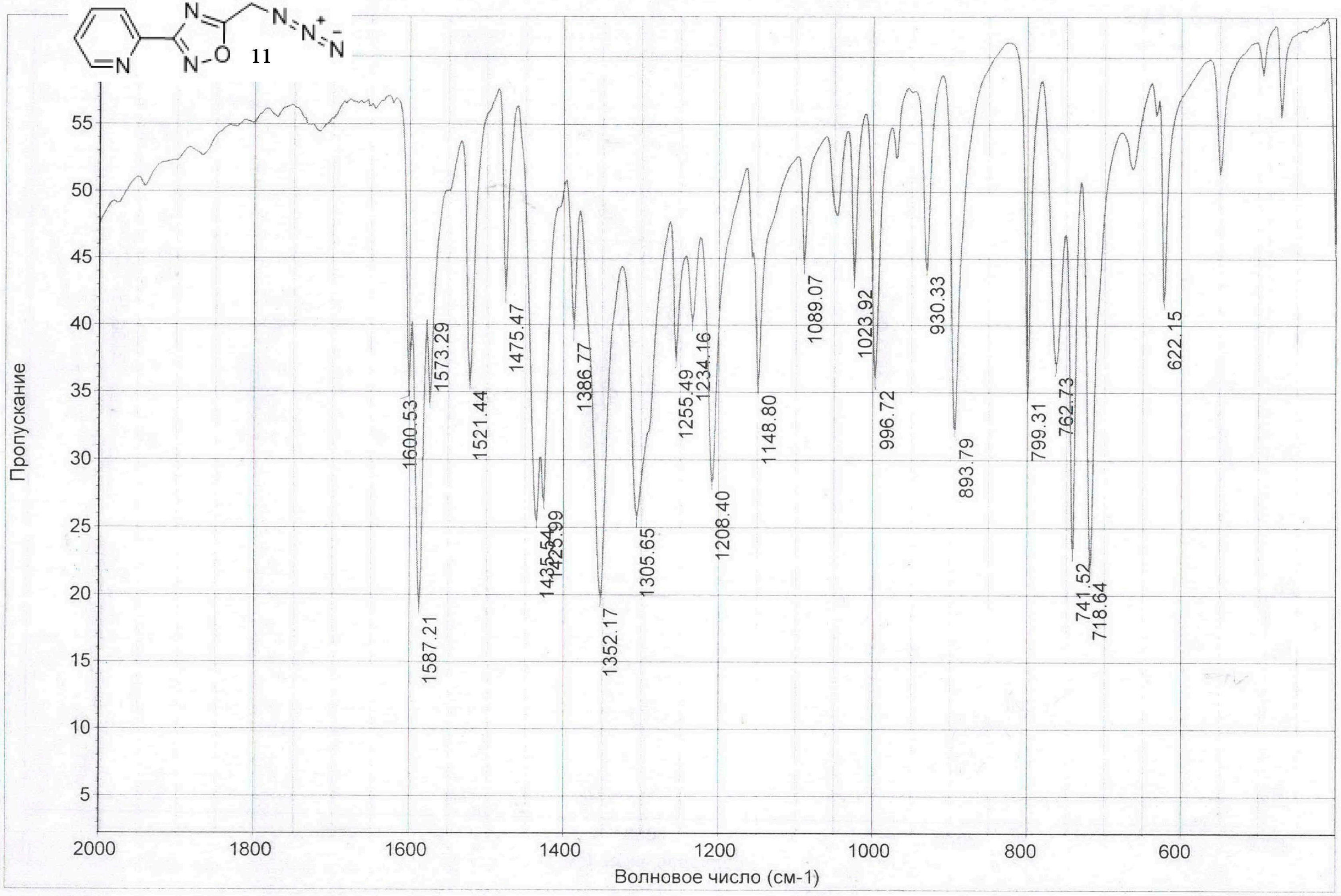
11
Пропускание
Волновое число (см-1)
55
50
45
40
35
30
25
20
15
10
5
2000
1800
1600
1400
1200
1000
800
600
1600.53
1587.21
1573.29
1521.44
1475.47
1435.54
1425.99
1386.77
1352.17
1305.65
1255.49
1234.16
1208.40
1148.80
1089.07
1023.92
996.72
930.33
893.79
799.31
762.73
741.52
718.64
622.15

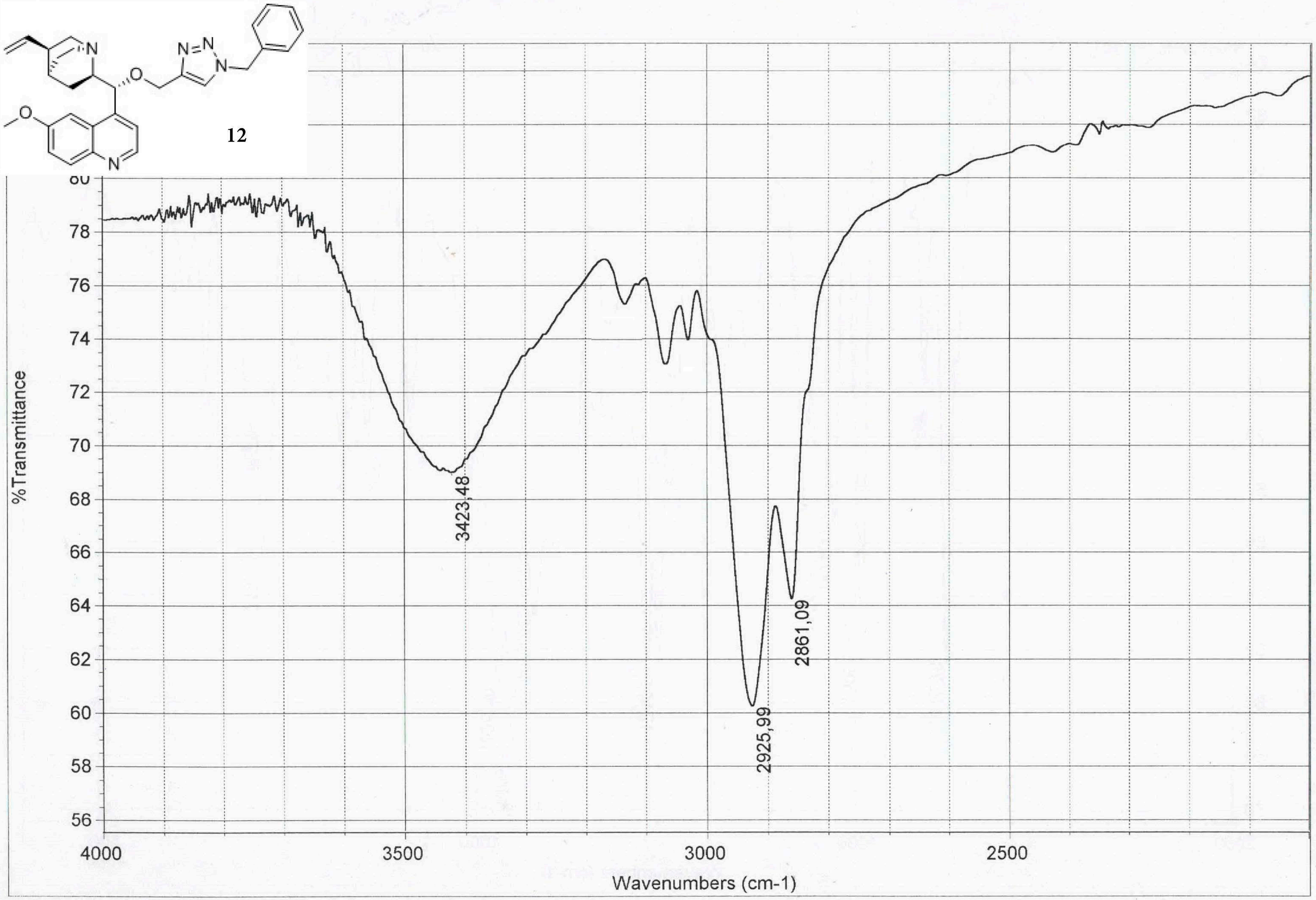

12

%Transmittance
Wavenumbers (cm-1)
3423,48
2925,99
2861,09
4000
3500
3000
2500

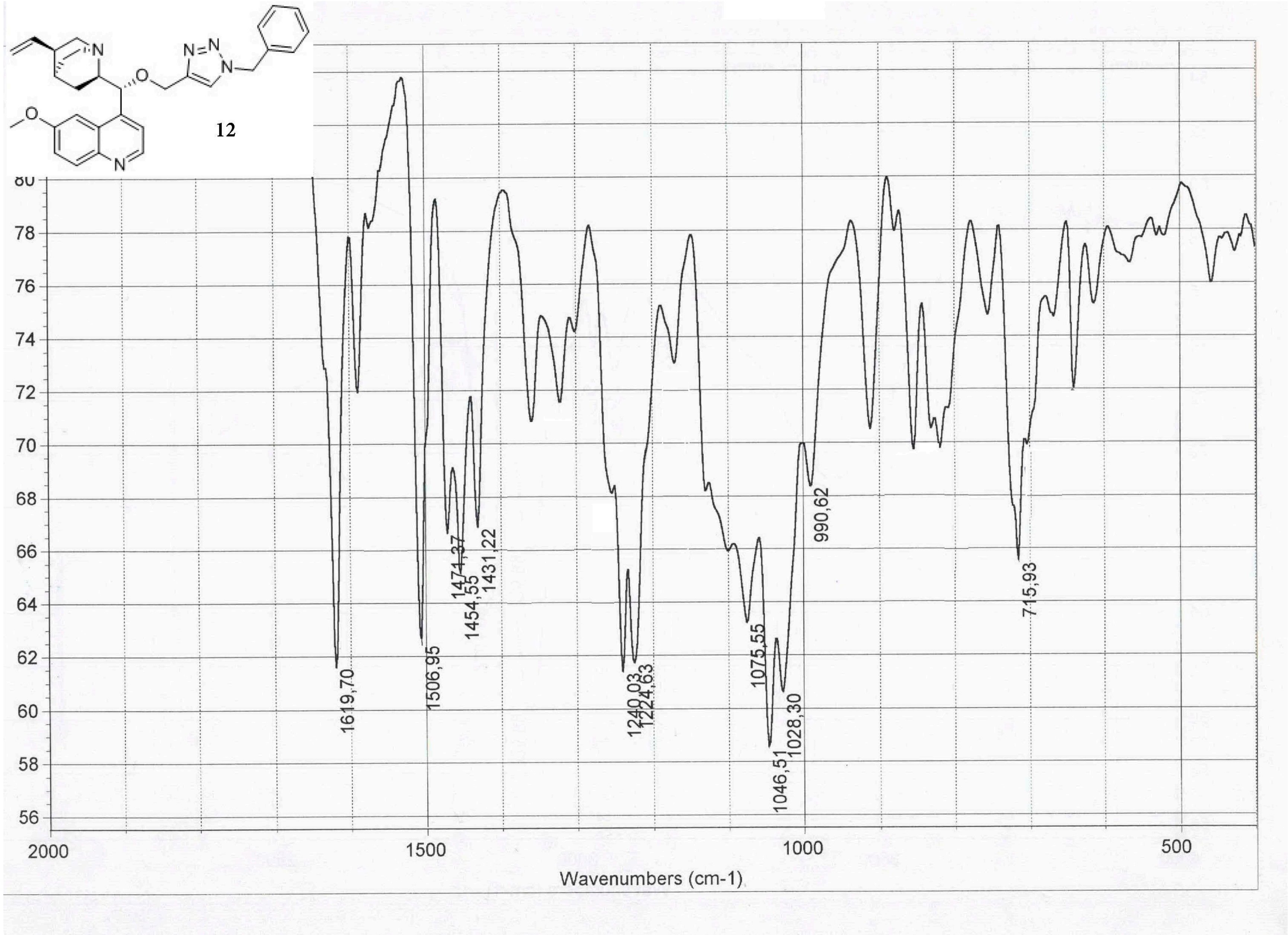
80
78
76
74
72
70
68
66
64
62
60
58
56
2000
1500
1000
500
Wavenumbers (cm-1)
1619,70
1506,95
1474,37
1454,55
1431,22
1240,03
1224,63
1075,55
1046,51
1028,30
990,62
715,93

12

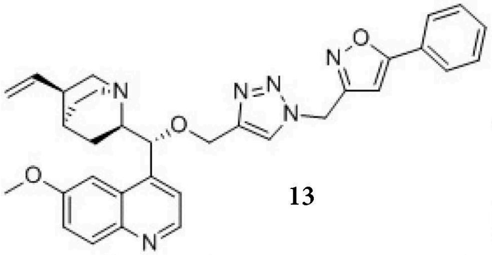
N
O
N=N
N
N
O
N
O

13

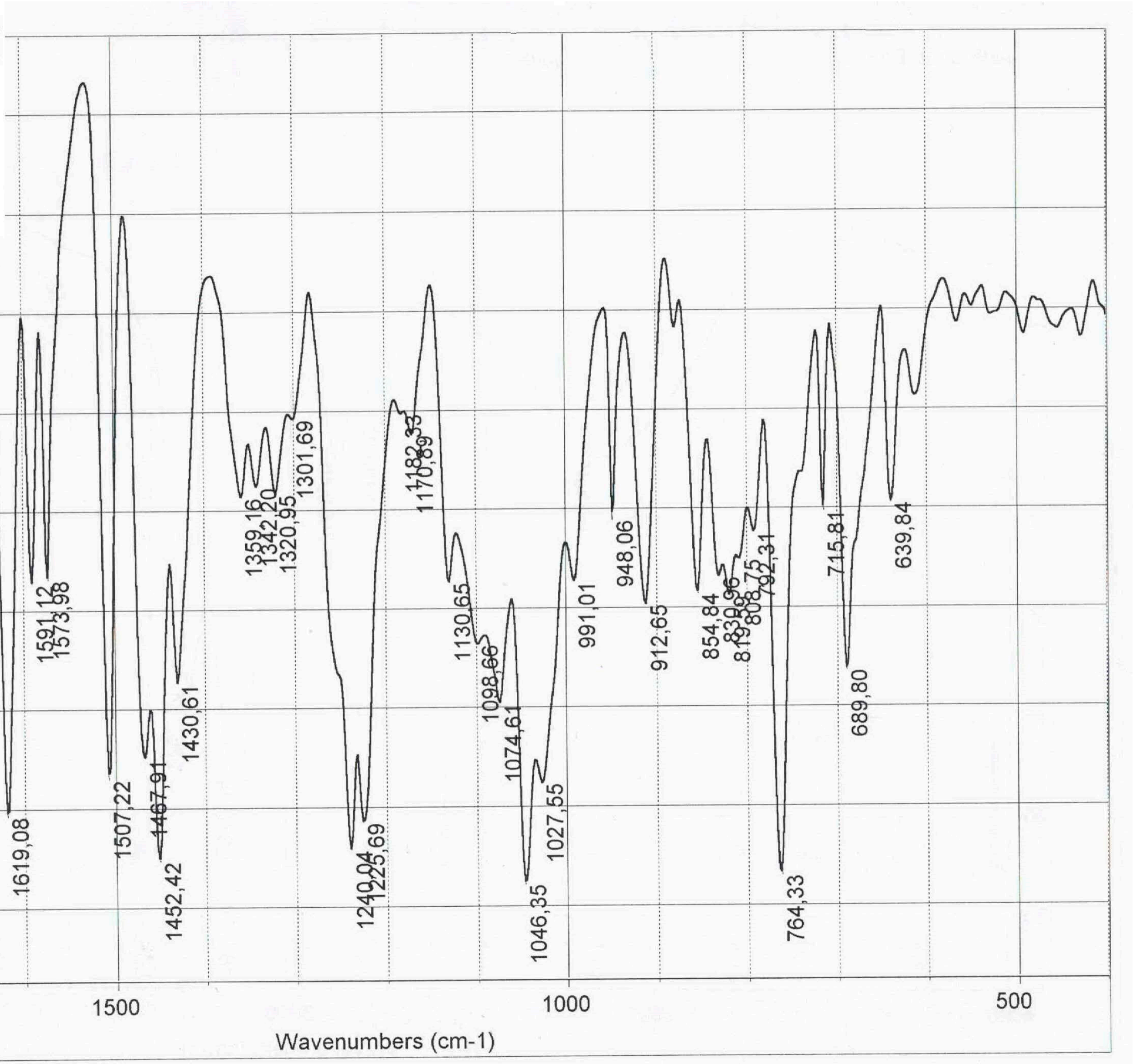
%Transmittance
Wavenumbers (cm-1)
2000
1500
1000
500
35
40
45
50
55
60
65
1619,08
1591,12
1573,98
1507,22
1467,91
1452,42
1430,61
1359,16
1342,20
1320,95
1301,69
1240,04
1225,69
1182,33
1170,89
1130,65
1098,66
1074,61
1046,35
1027,55
991,01
948,06
912,65
854,84
839,96
819,98
808,75
792,31
764,33
715,81
689,80
639,84

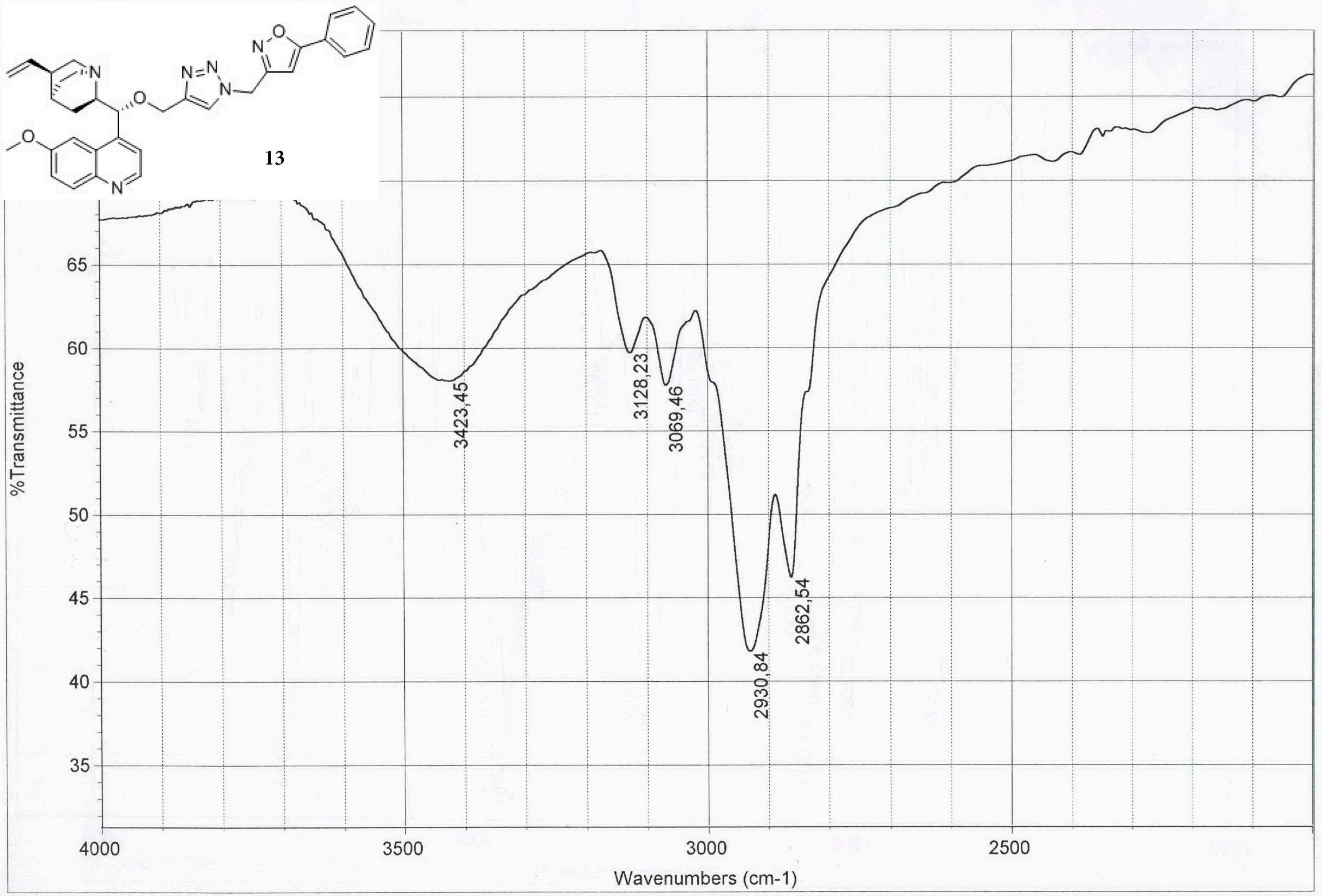
13
%Transmittance
65
60
55
50
45
40
35
4000
3500
3000
2500
Wavenumbers (cm-1)
3423,45
3128,23
3069,46
2930,84
2862,54

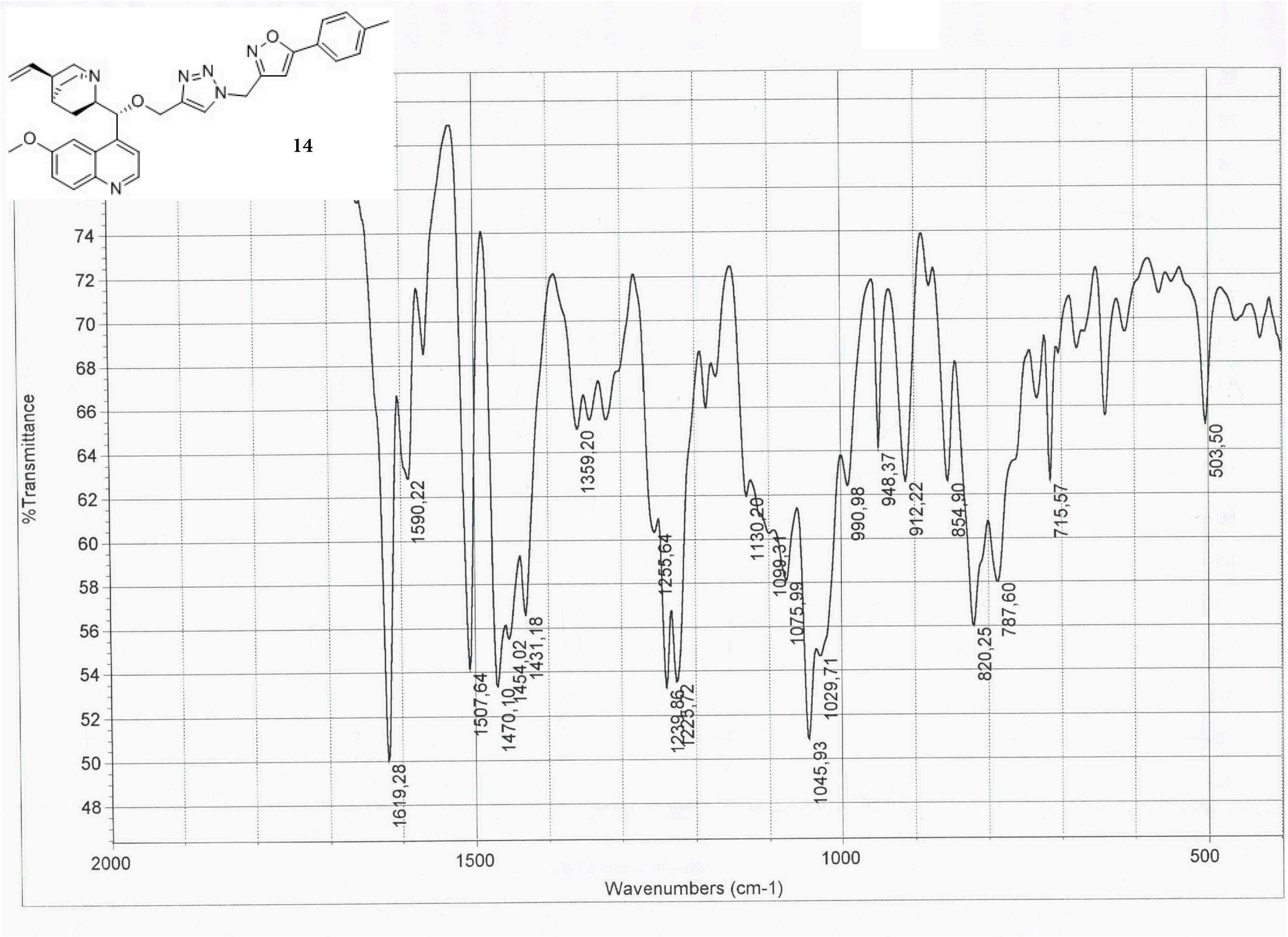
14
%Transmittance
Wavenumbers (cm-1)
74
72
70
68
66
64
62
60
58
56
54
52
50
48
2000
1500
1000
500
1619,28
1590,22
1507,64
1470,10
1454,02
1431,18
1359,20
1255,64
1239,86
1225,72
1130,20
1099,31
1075,99
1045,93
1029,71
990,98
948,37
912,22
854,90
820,25
787,60
715,57
503,50

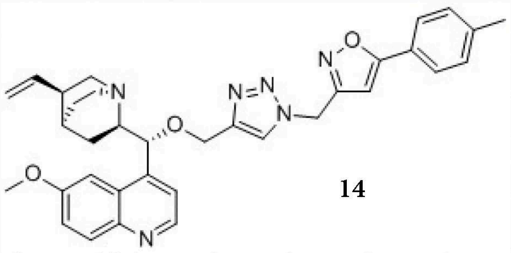

14

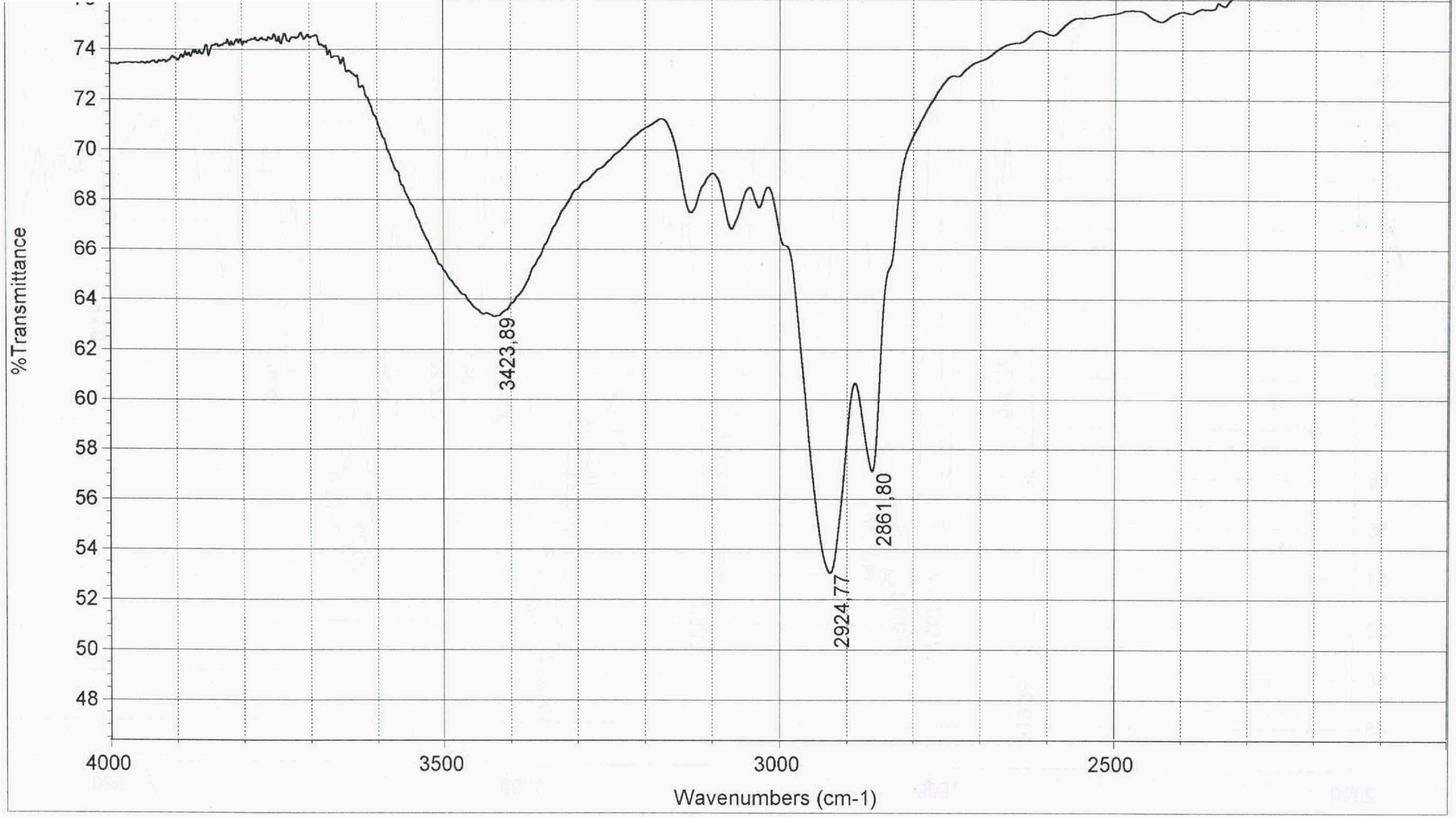

%Transmittance
74
72
70
68
66
64
62
60
58
56
54
52
50
48
3423,89
2924,77
2861,80
4000
3500
3000
2500
Wavenumbers (cm-1)

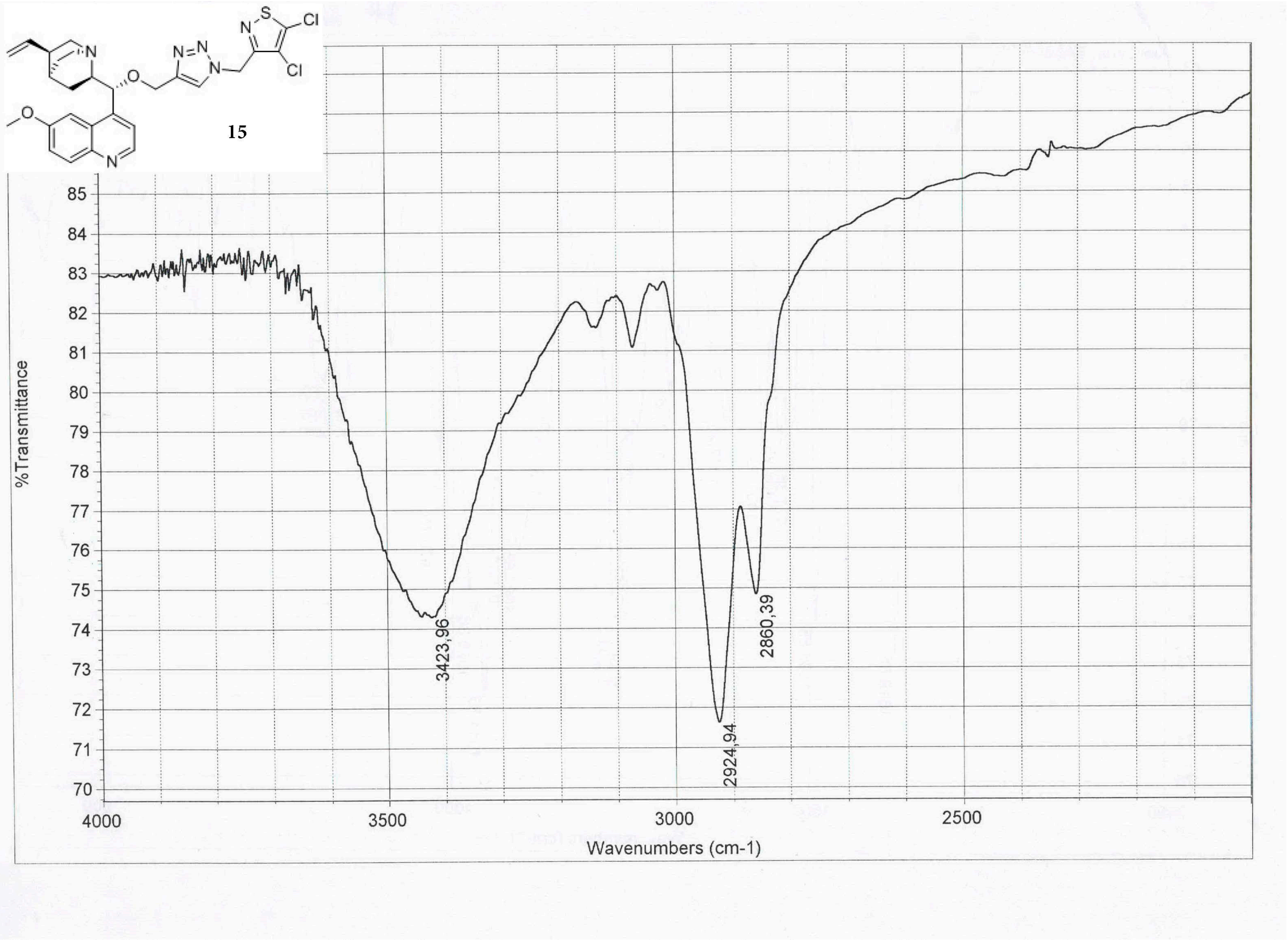
15
%Transmittance
Wavenumbers (cm-1)
3423,96
2924,94
2860,39
4000
3500
3000
2500

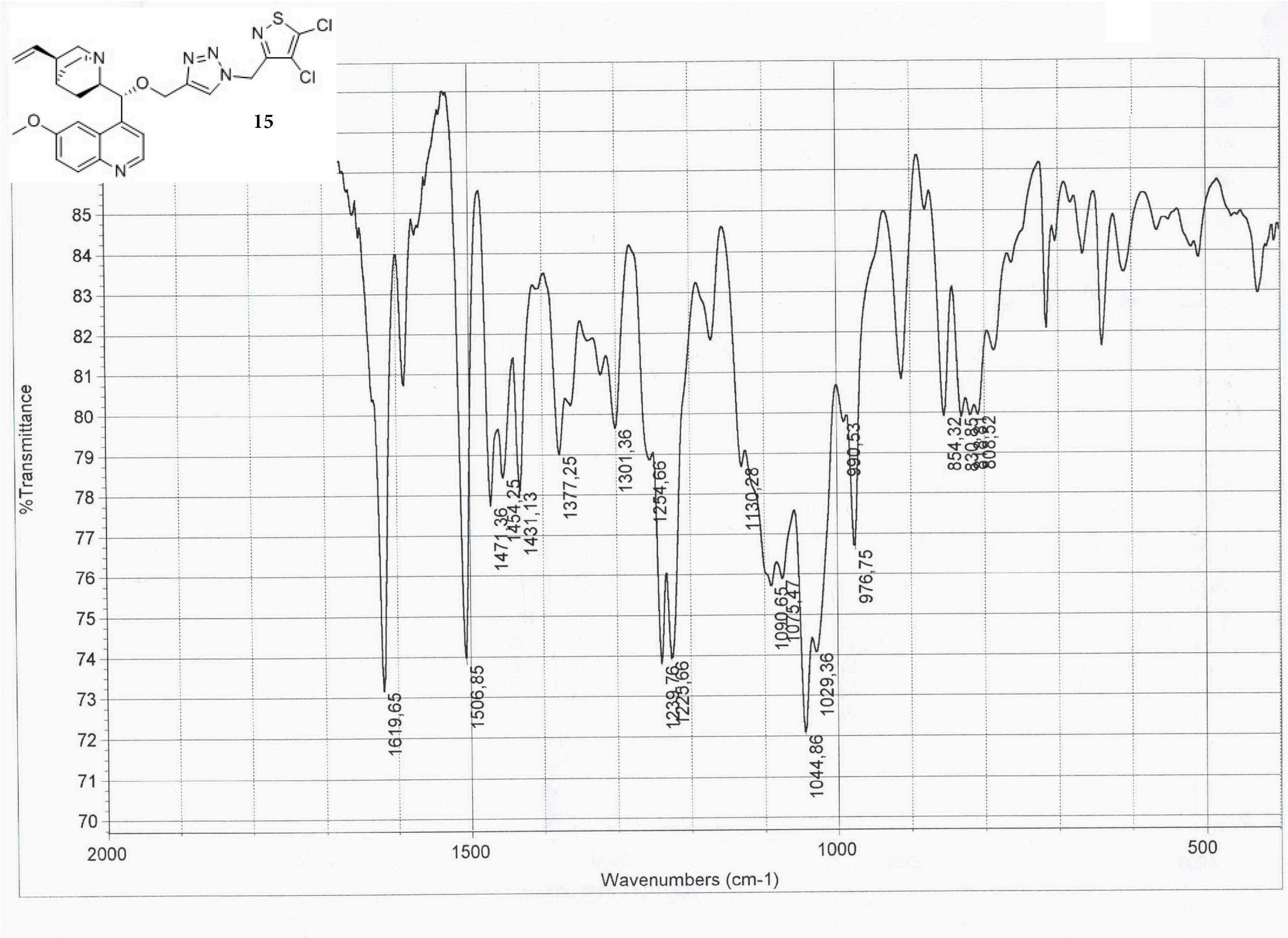

15
%Transmittance
Wavenumbers (cm-1)
2000
1500
1000
500
85
84
83
82
81
80
79
78
77
76
75
74
73
72
71
70
1619,65
1506,85
1471,36
1454,25
1431,13
1377,25
1301,36
1254,66
1239,76
1225,66
1130,28
1090,65
1075,47
1044,86
1029,36
990,53
976,75
854,32
830,85
808,32

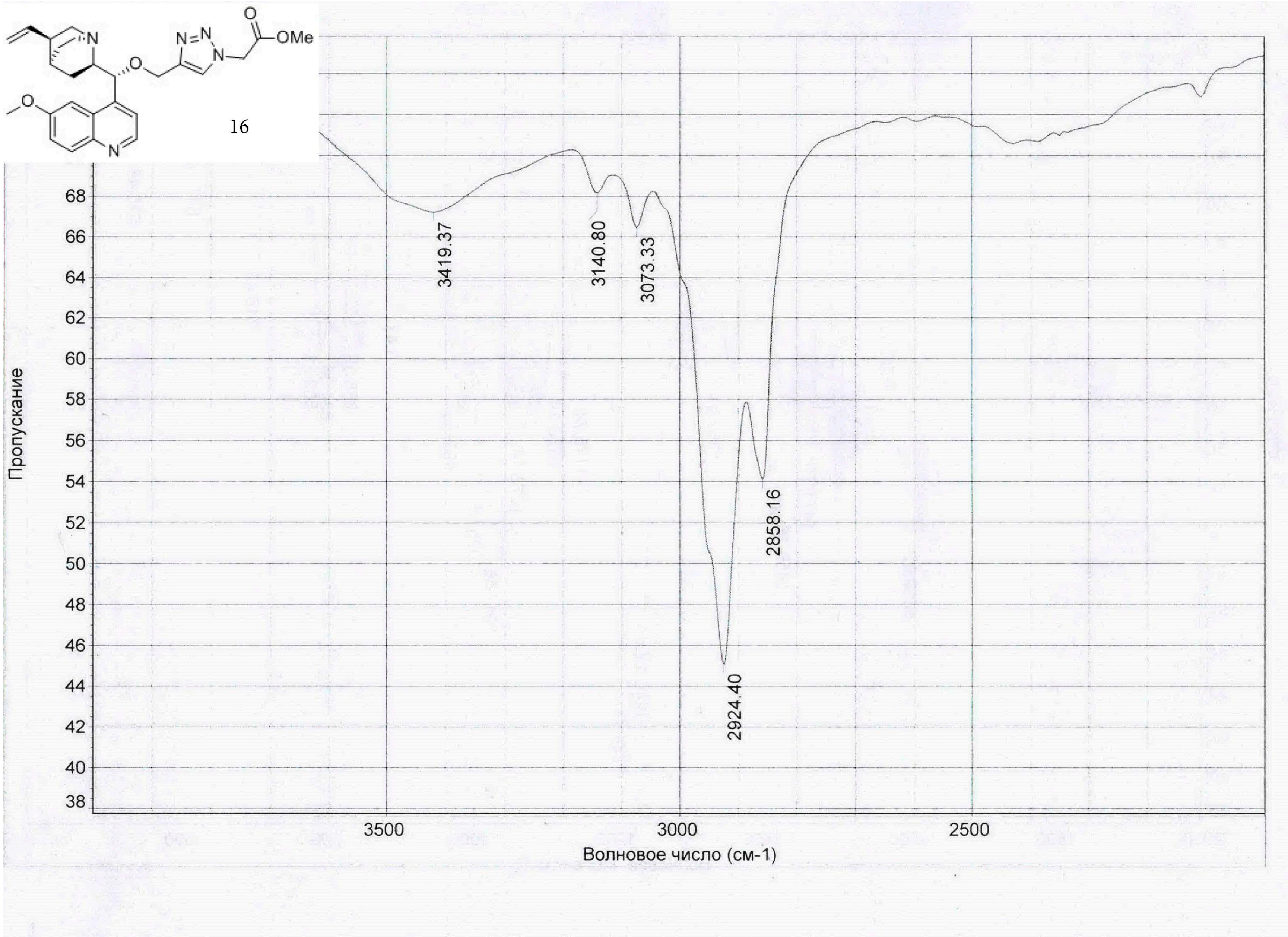
OMe
16
Пропускание
68
66
64
62
60
58
56
54
52
50
48
46
44
42
40
38
3419.37
3140.80
3073.33
2924.40
2858.16
3500
3000
2500
Волновое число (см-1)

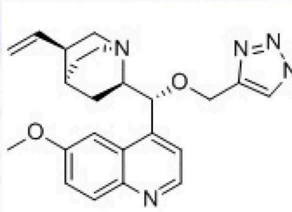
N
O
N
N
N
N
O
OMe
O
N

16

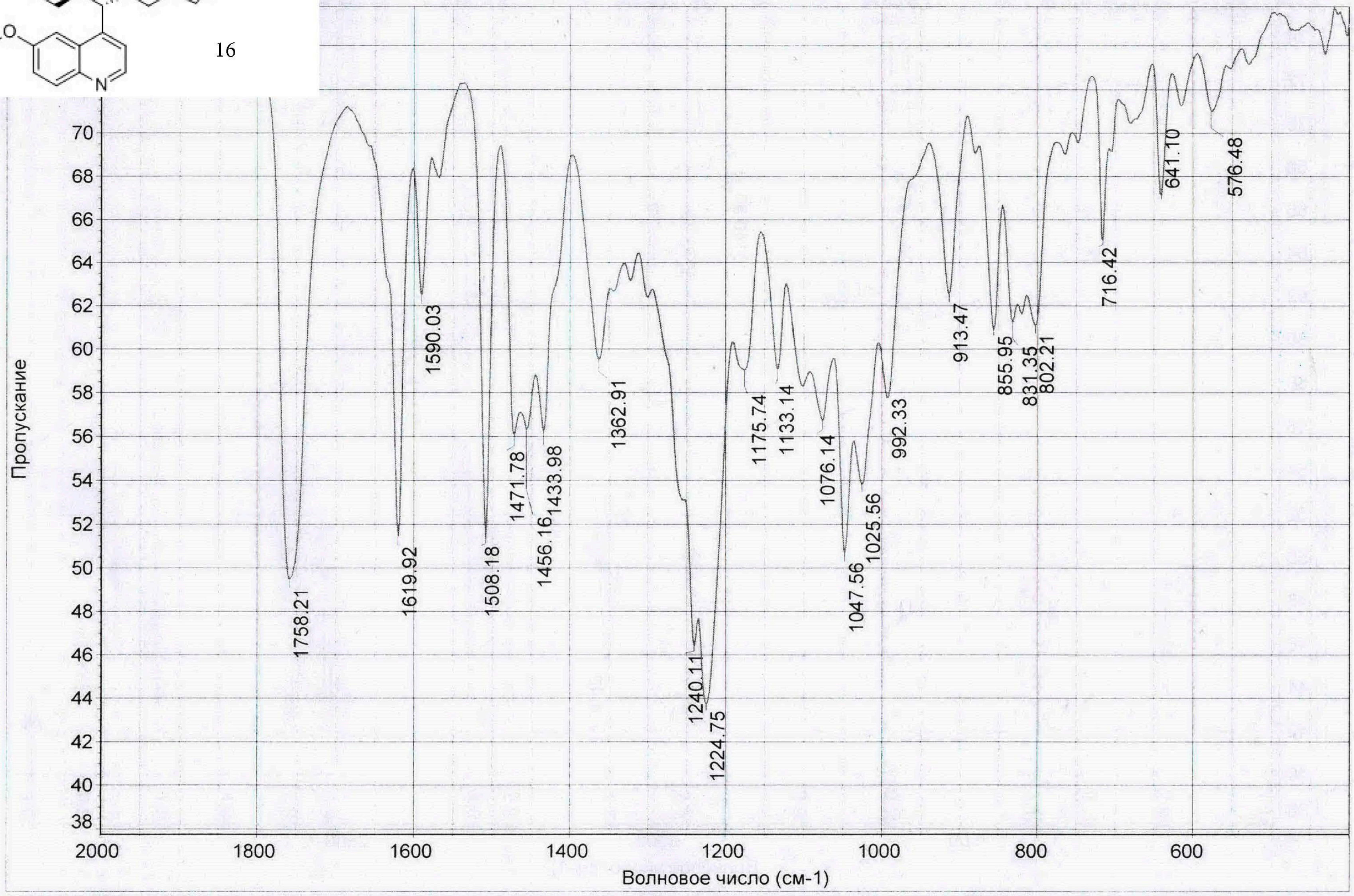
Пропускание
Волновое число (см-1)
1758.21
1619.92
1590.03
1508.18
1471.78
1456.16
1433.98
1362.91
1240.11
1224.75
1175.74
1133.14
1076.14
1047.56
1025.56
992.33
913.47
855.95
831.35
802.21
716.42
641.10
576.48

3. Copy of the NMR spectra and the data of mass-spectrometry

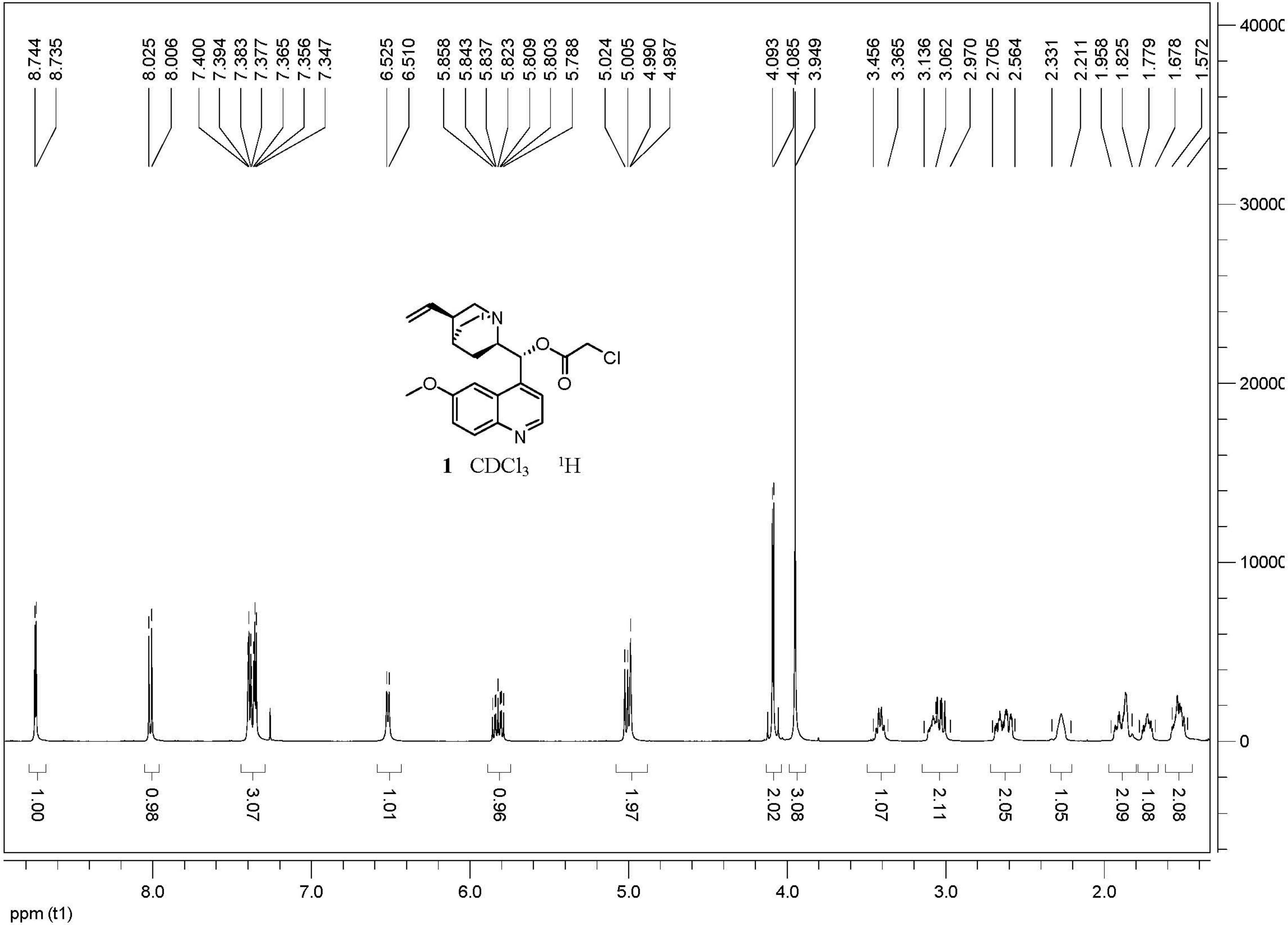

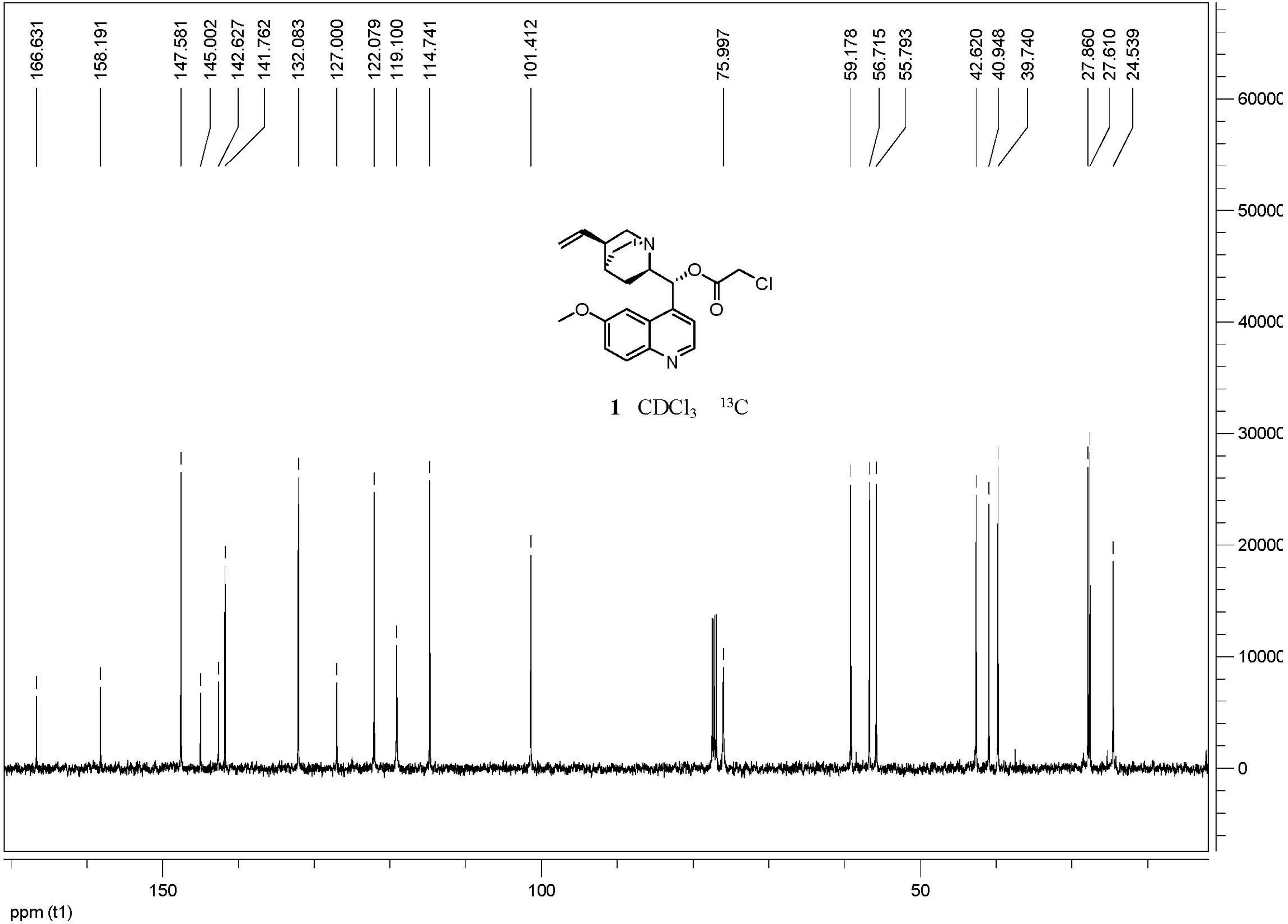
166.631
158.191
147.581
145.002
142.627
141.762
132.083
127.000
122.079
119.100
114.741
101.412
75.997
59.178
56.715
55.793
42.620
40.948
39.740
27.860
27.610
24.539
N
O
Cl
O
O
N
1 CDCl3 13C
60000
50000
40000
30000
20000
10000
0
150
100
50
ppm (t1)

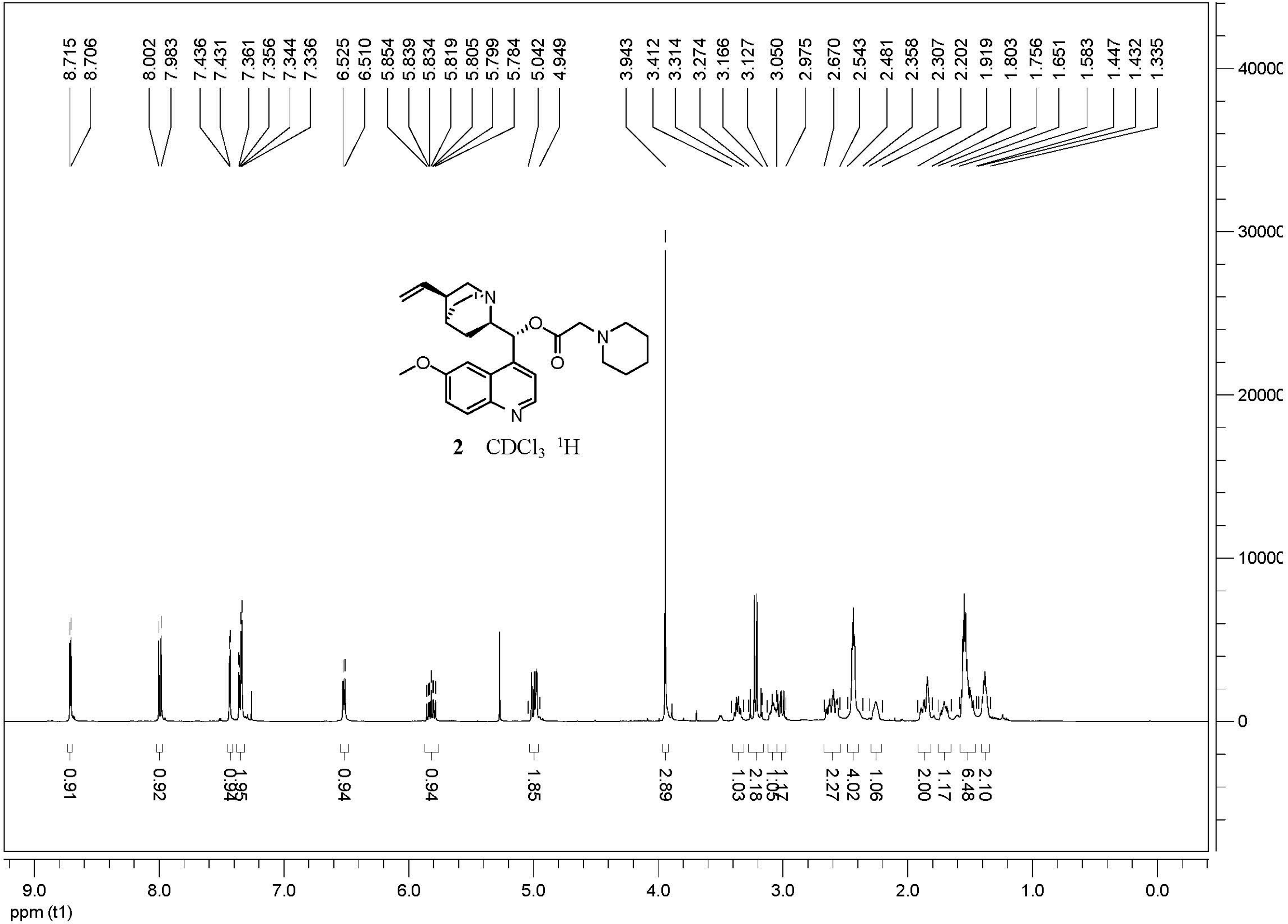
8.715
8.706
8.002
7.983
7.436
7.431
7.361
7.356
7.344
7.336
6.525
6.510
5.854
5.839
5.834
5.819
5.805
5.799
5.784
5.042
4.949
3.943
3.412
3.314
3.274
3.166
3.127
3.050
2.975
2.670
2.543
2.481
2.358
2.307
2.202
1.919
1.803
1.756
1.651
1.583
1.447
1.432
1.335
N
O
O
N
O
N
2 CDCl3 1H
0.91
0.92
0.92
1.95
0.94
0.94
1.85
2.89
1.03
2.18
1.05
1.17
2.27
4.02
1.06
2.00
1.17
6.48
2.10
9.0
8.0
7.0
6.0
5.0
4.0
3.0
2.0
1.0
0.0
ppm (t1)
40000
30000
20000
10000
0

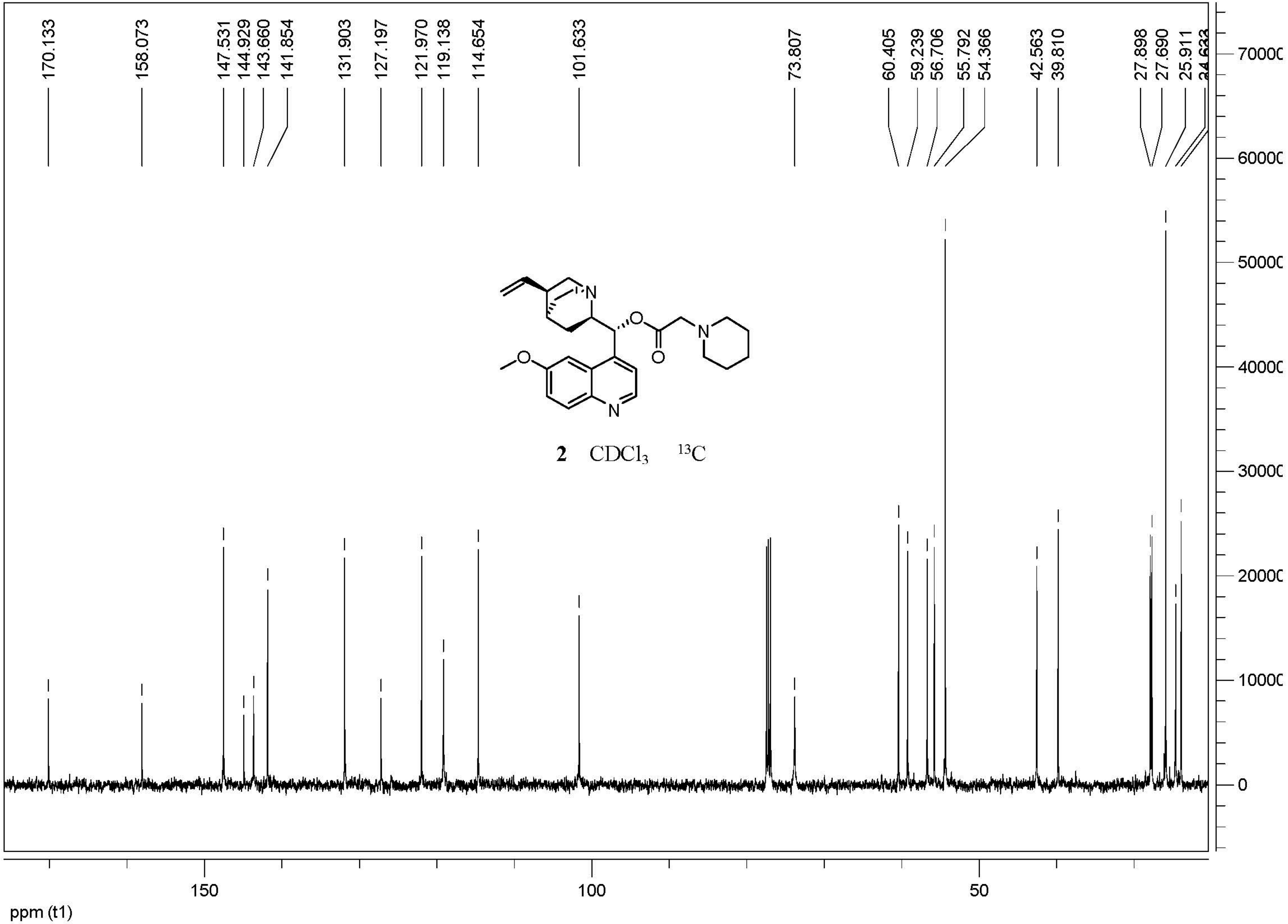
170.133
158.073
147.531
144.929
143.660
141.854
131.903
127.197
121.970
119.138
114.654
101.633
73.807
60.405
59.239
56.706
55.792
54.366
42.563
39.810
27.898
27.690
25.911
2 CDCl3 13C
150
100
50
ppm (t1)
70000
60000
50000
40000
30000
20000
10000
0

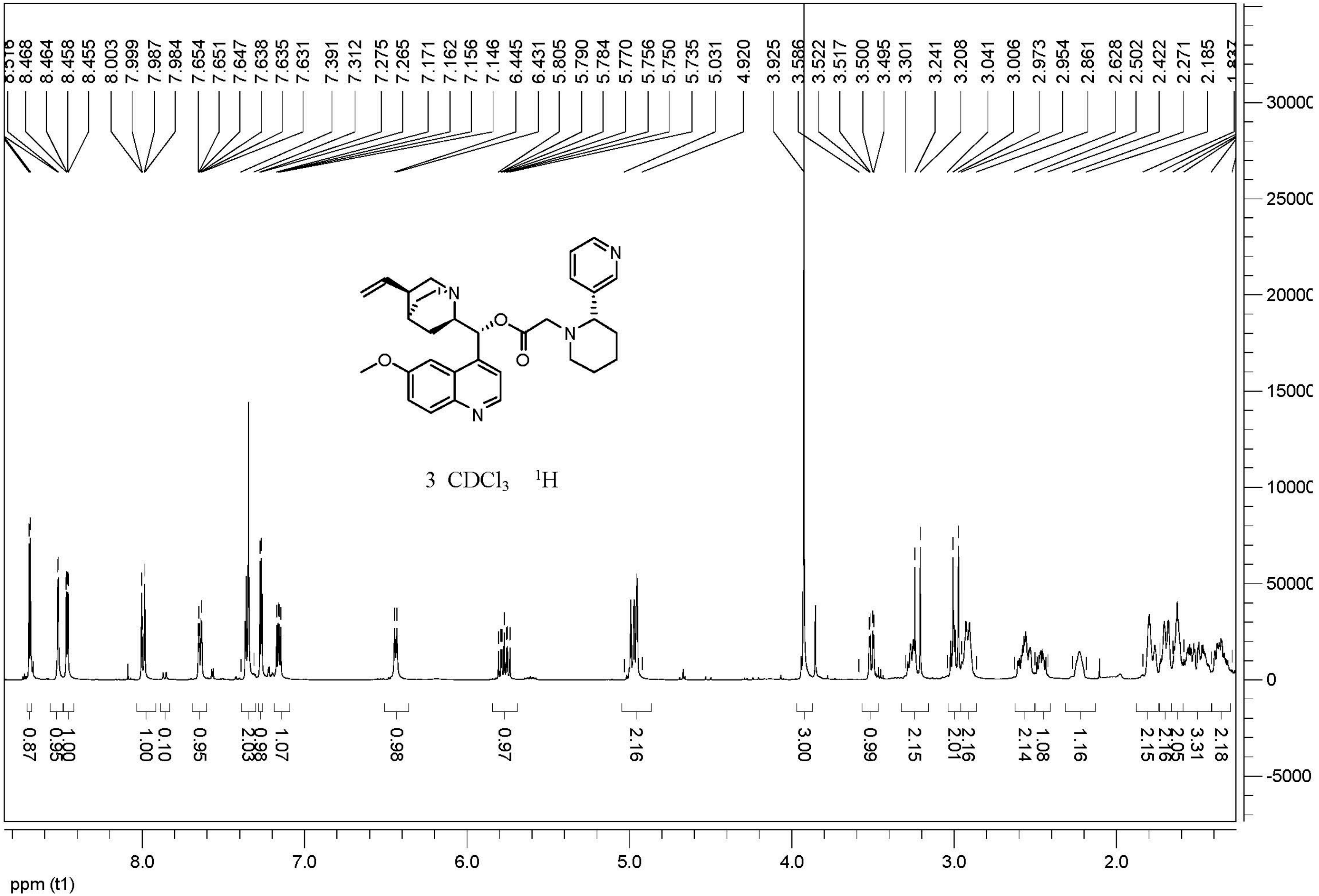
3 CDCl3 1H
ppm (t1)
8.0
7.0
6.0
5.0
4.0
3.0
2.0

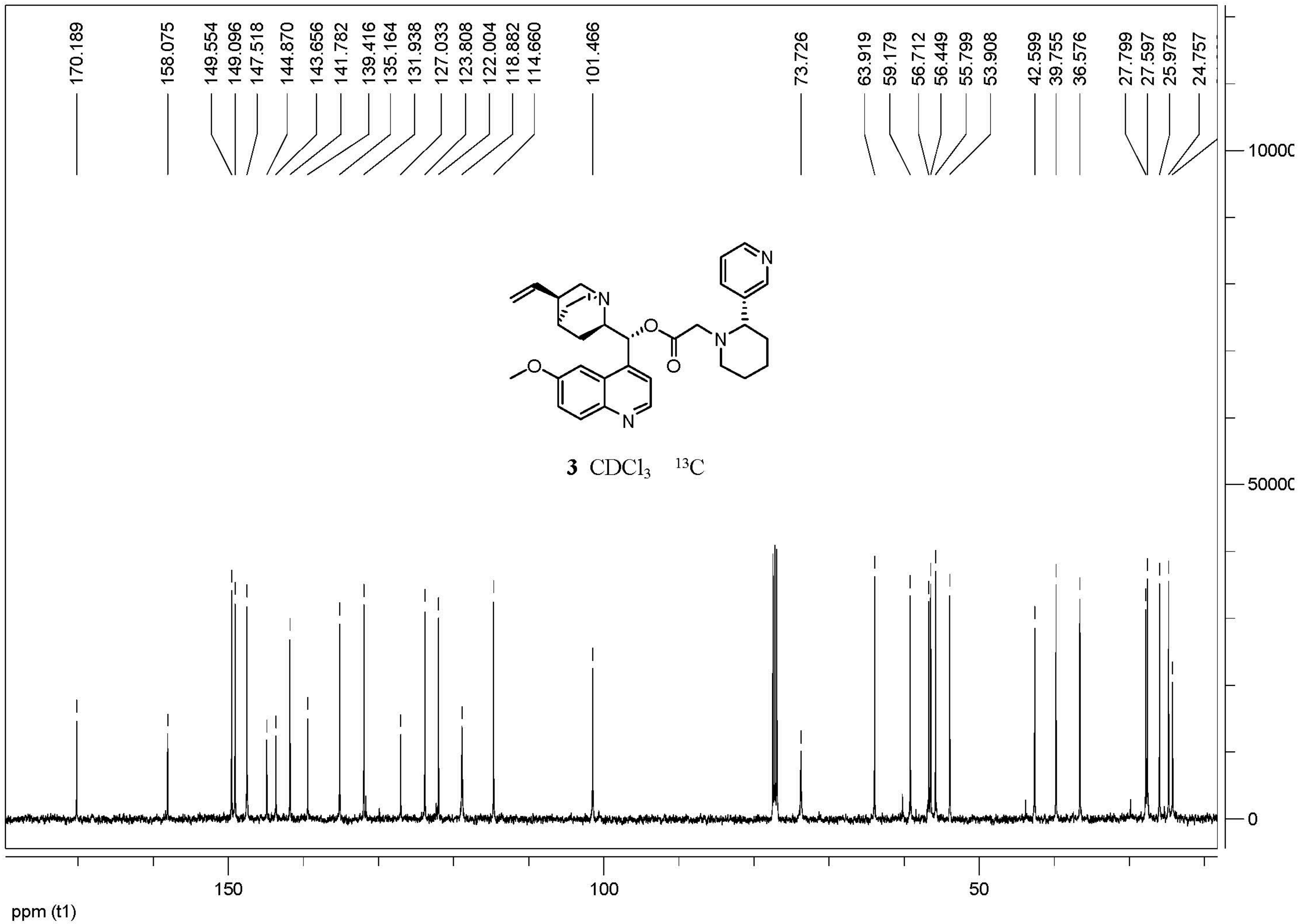
170.189
158.075
149.554
149.096
147.518
144.870
143.656
141.782
139.416
135.164
131.938
127.033
123.808
122.004
118.882
114.660
101.466
73.726
63.919
59.179
56.712
56.449
55.799
53.908
42.599
39.755
36.576
27.799
27.597
25.978
24.757
3 CDCl3 13C
150
100
50
ppm (t1)
0

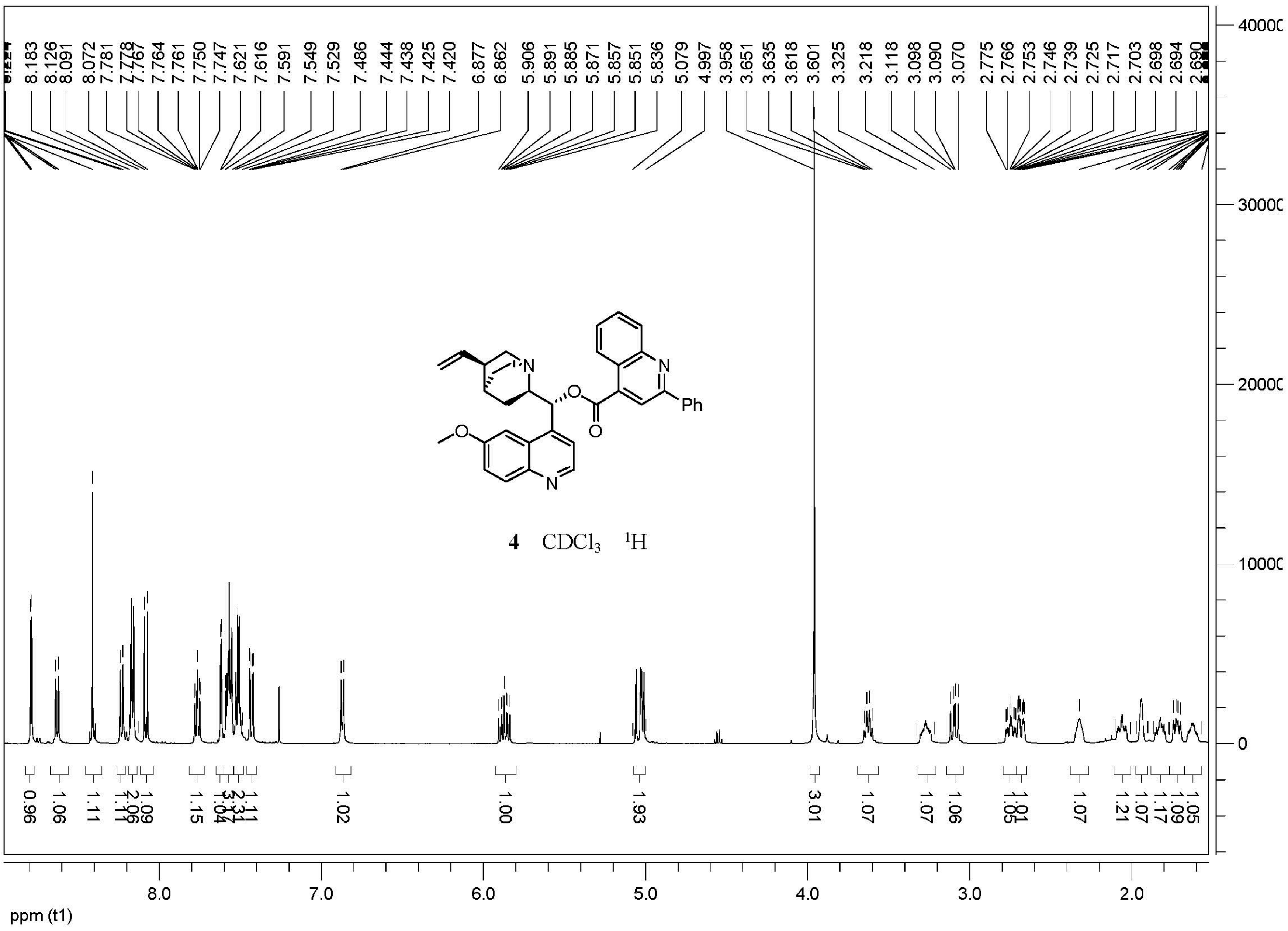
4 CDCl$_3$ ^{1}H
ppm (t1)

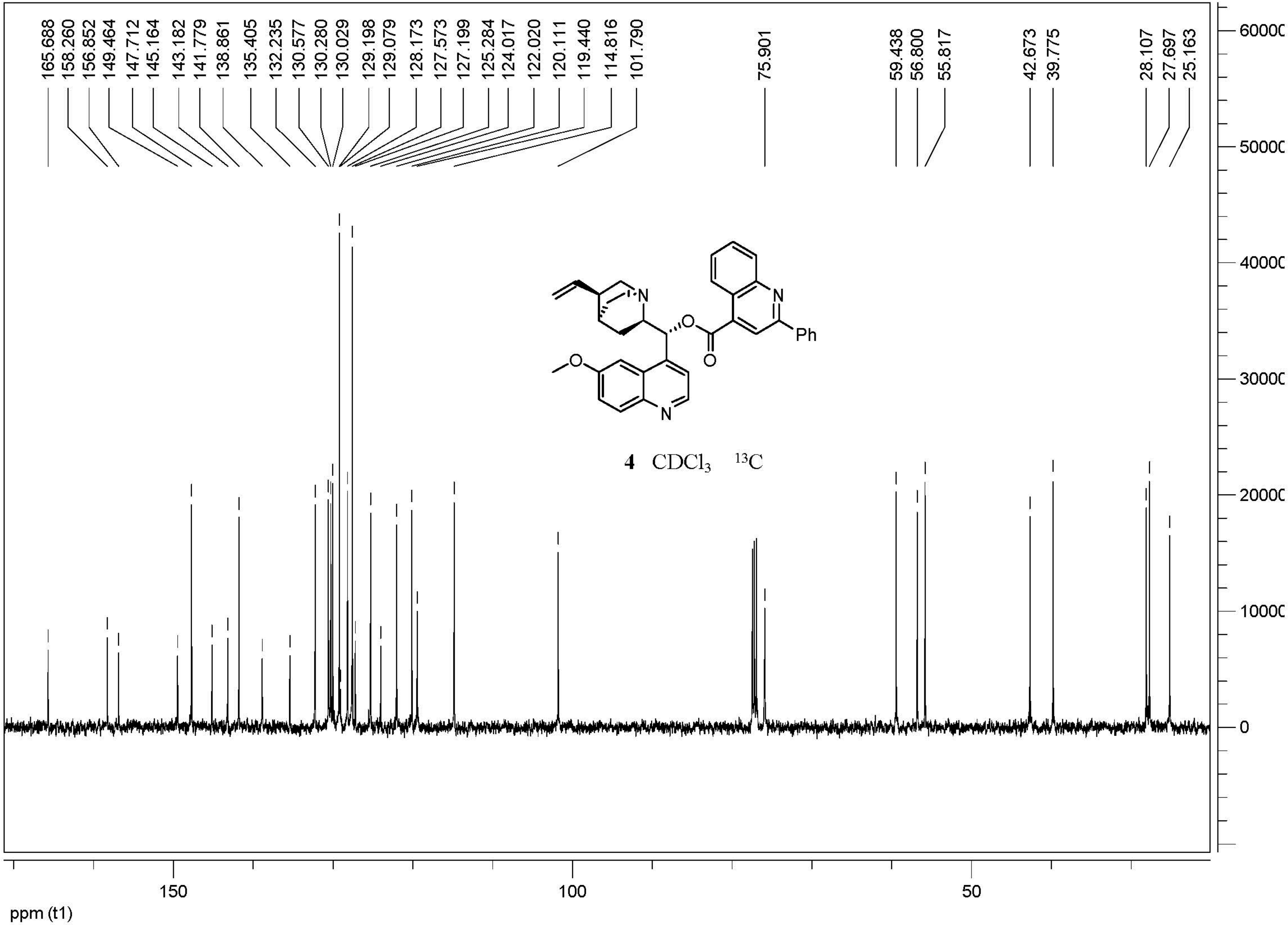
165.688
158.260
156.852
149.464
147.712
145.164
143.182
141.779
138.861
135.405
132.235
130.577
130.280
130.029
129.198
129.079
128.173
127.573
127.199
125.284
124.017
122.020
120.111
119.440
114.816
101.790
75.901
59.438
56.800
55.817
42.673
39.775
28.107
27.697
25.163
N
O
O
N
Ph
O
N
4 $CDCl_3$ ^{13}C
60000
50000
40000
30000
20000
10000
0
150
100
50
ppm (t1)

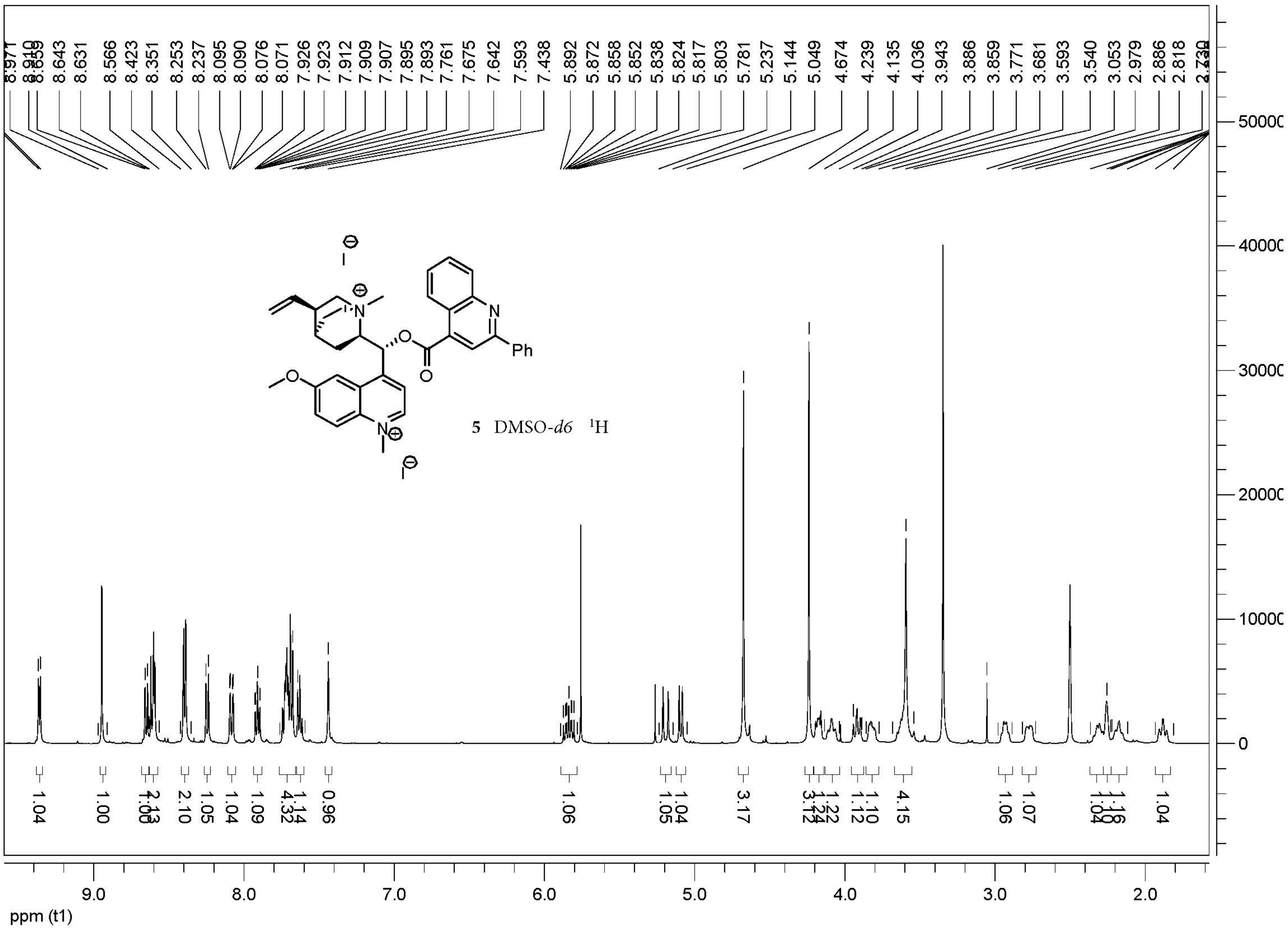
5 DMSO-d6 1H
ppm (t1)
9.0
8.0
7.0
6.0
5.0
4.0
3.0
2.0
Ph
N
O

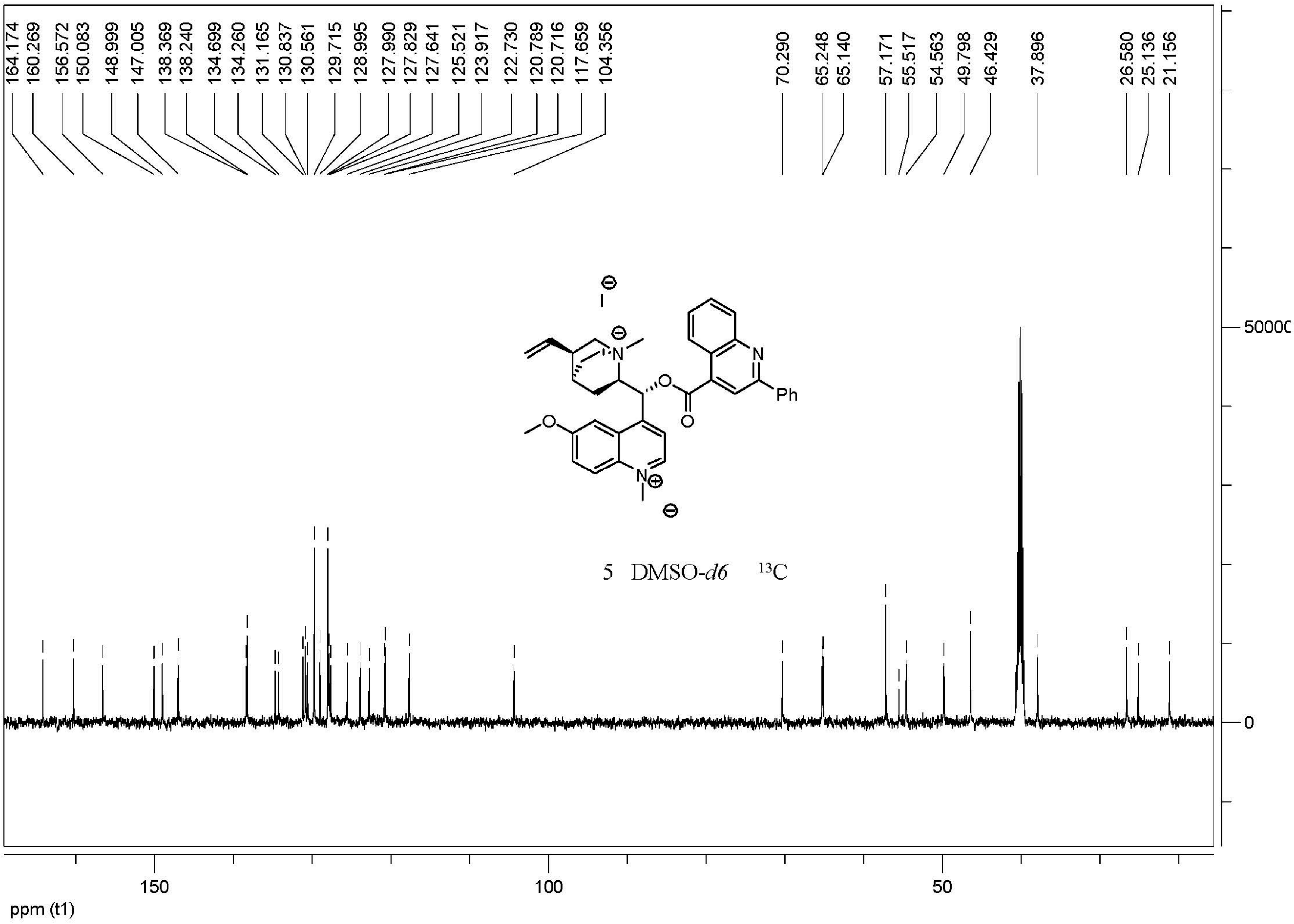
164.174
160.269
156.572
150.083
148.999
147.005
138.369
138.240
134.699
134.260
131.165
130.837
130.561
129.715
128.995
127.990
127.829
127.641
125.521
123.917
122.730
120.789
120.716
117.659
104.356
70.290
65.248
65.140
57.171
55.517
54.563
49.798
46.429
37.896
26.580
25.136
21.156
I
N
O
N
Ph
O
O
N
5 DMSO-d6 13C
50000
0
150
100
50
ppm (t1)

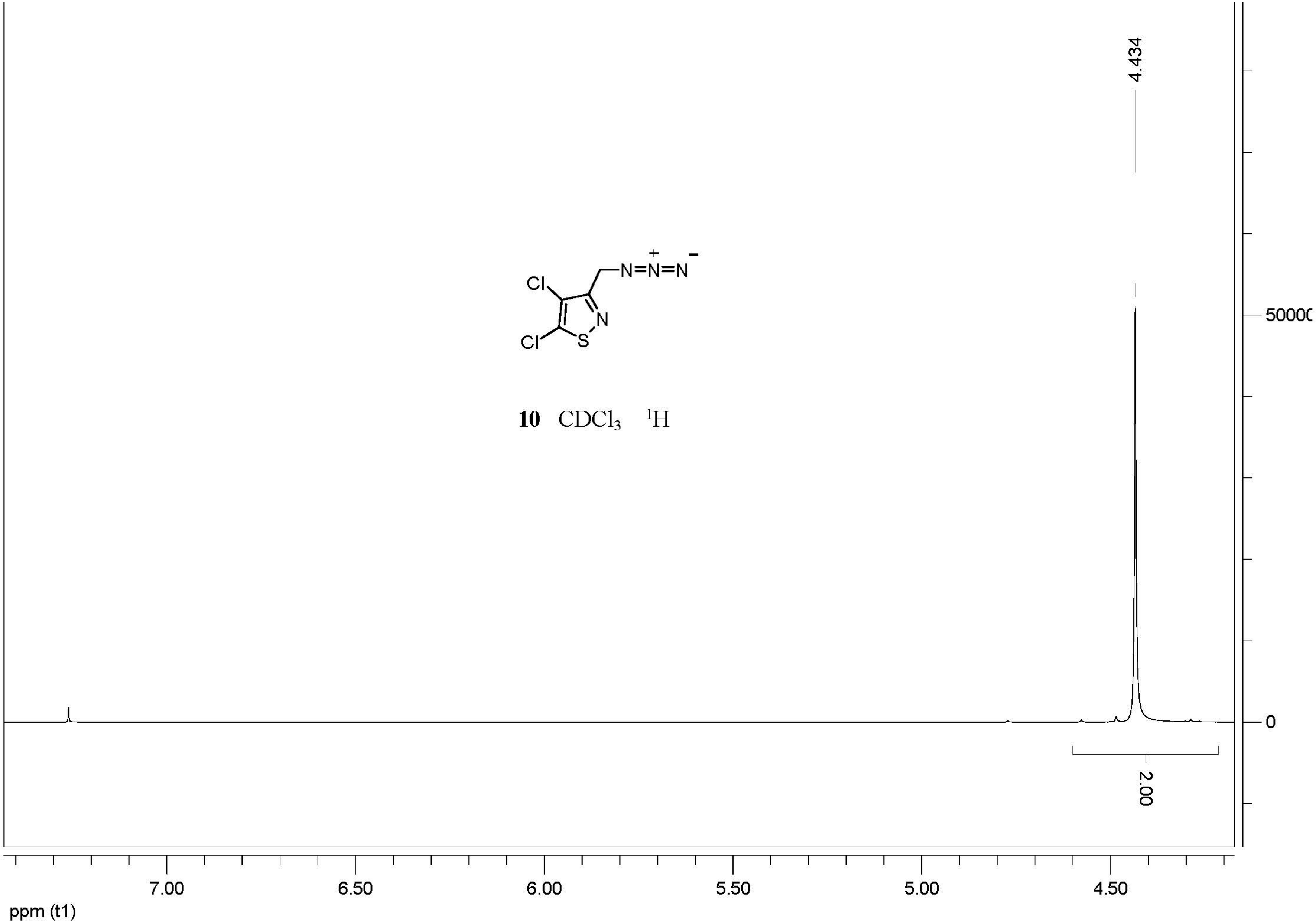
4.434
Cl
Cl
N
S
N=N=N
10 CDCl3 1H
50000
0
2.00
7.00
6.50
6.00
5.50
5.00
4.50
ppm (t1)

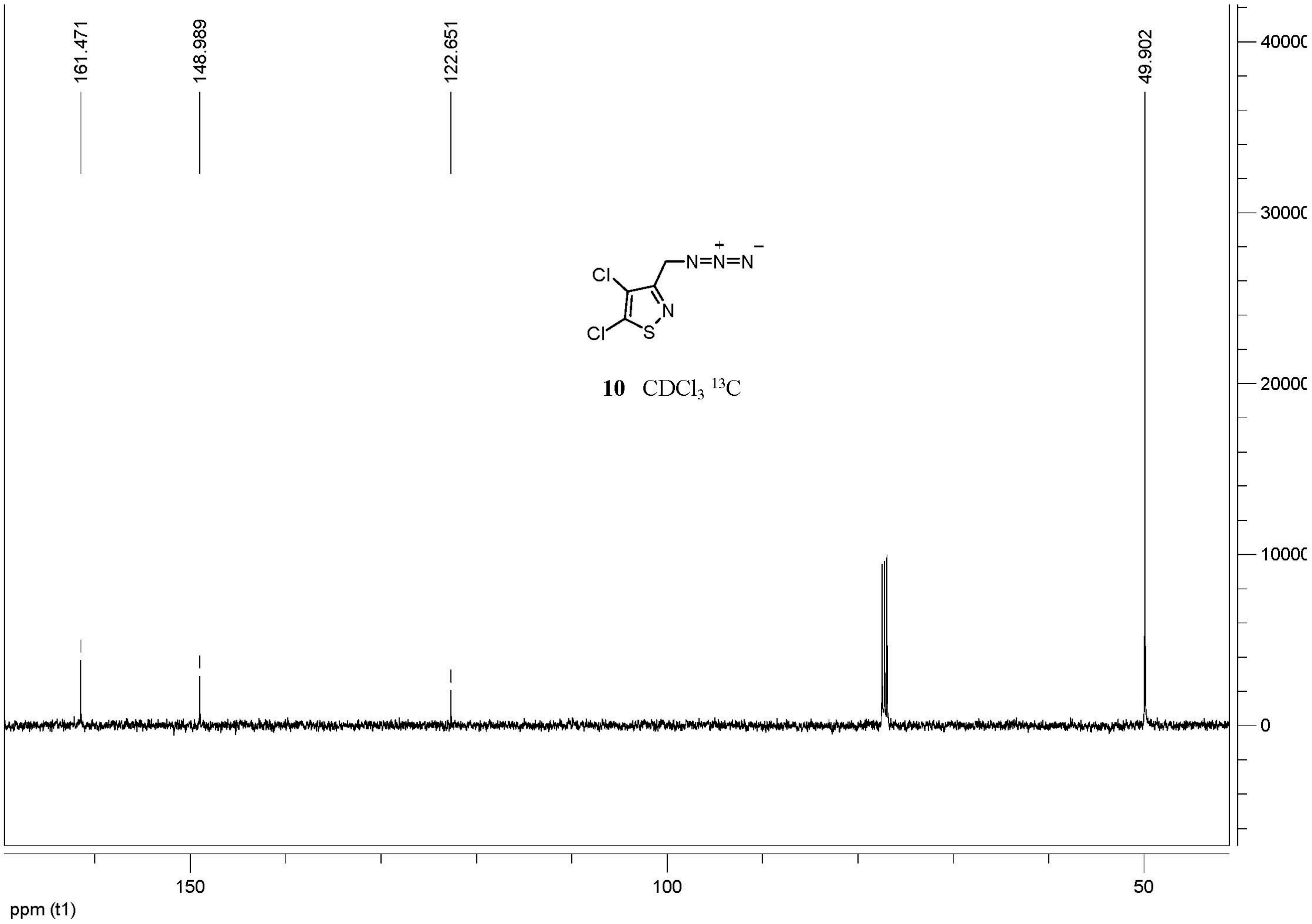
161.471
148.989
122.651
49.902
Cl
Cl
N
S
N=N=N
10 CDCl3 13C
40000
30000
20000
10000
0
150
100
50
ppm (t1)

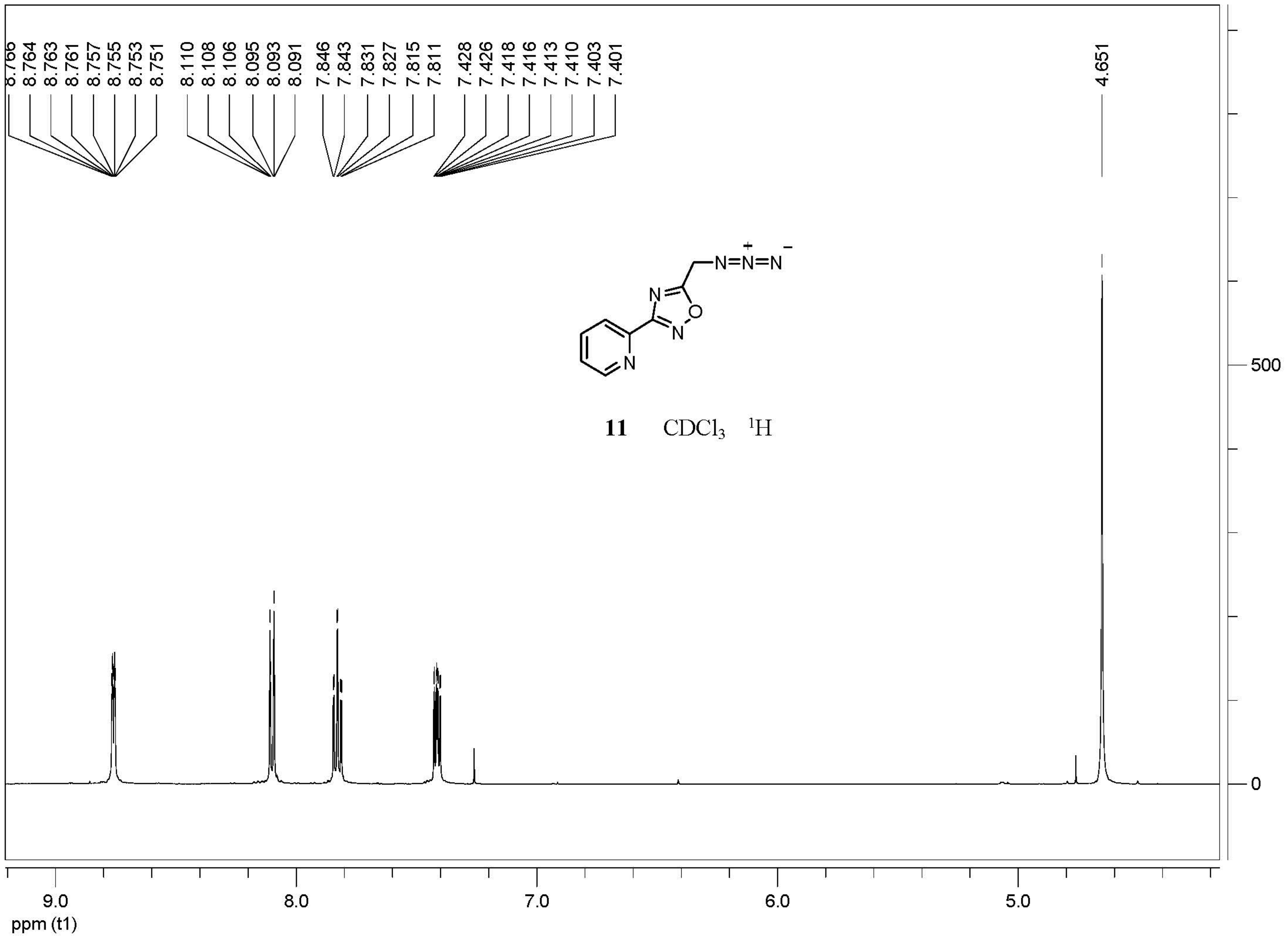

8.766
8.764
8.763
8.761
8.757
8.755
8.753
8.751
8.110
8.108
8.106
8.095
8.093
8.091
7.846
7.843
7.831
7.827
7.815
7.811
7.428
7.426
7.418
7.416
7.413
7.410
7.403
7.401
4.651
11 CDCl3 1H
500
0
9.0
8.0
7.0
6.0
5.0
ppm (t1)

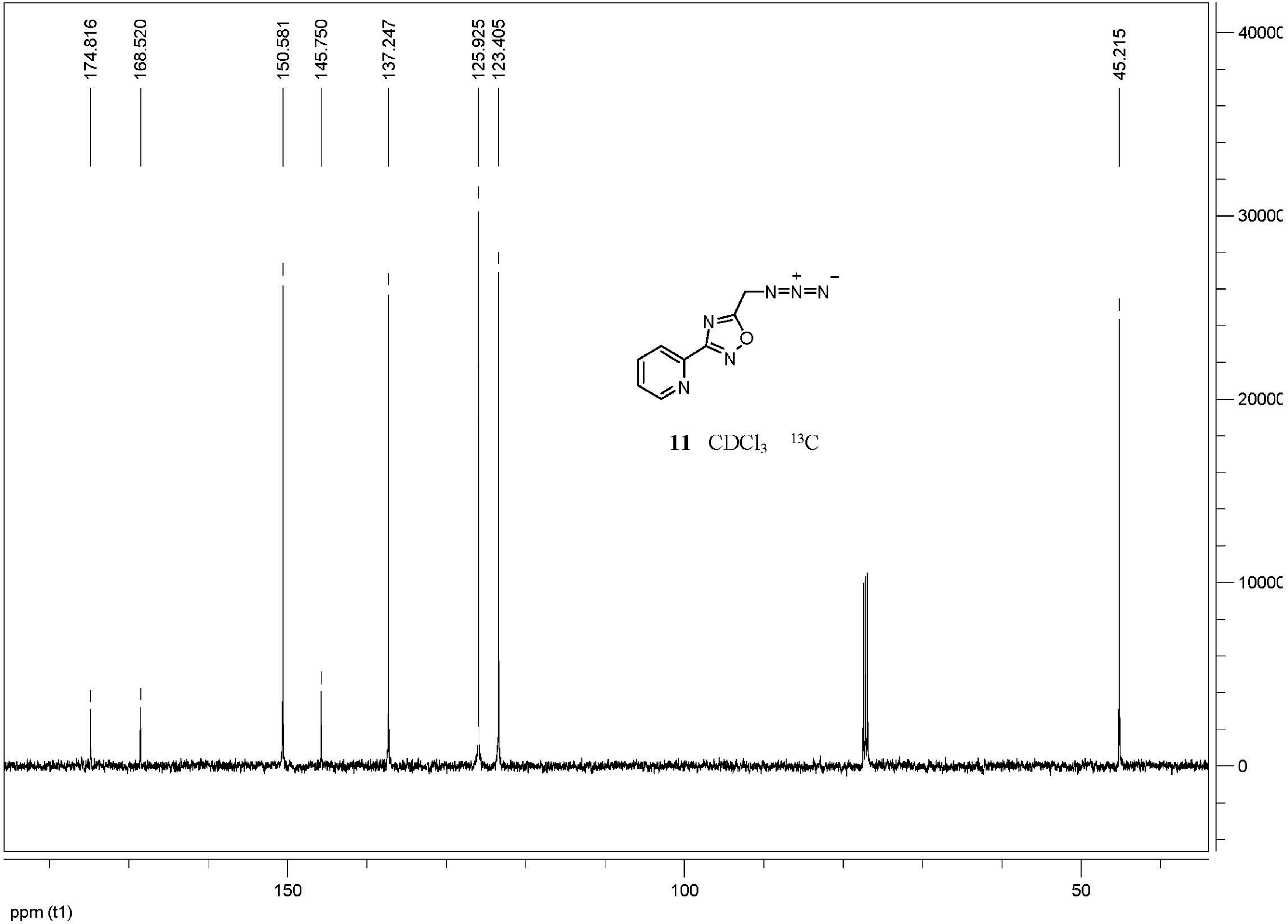
174.816
168.520
150.581
145.750
137.247
125.925
123.405
45.215
N=N=N
N
O
N
N
11 CDCl3 13C
40000
30000
20000
10000
0
150
100
50
ppm (t1)

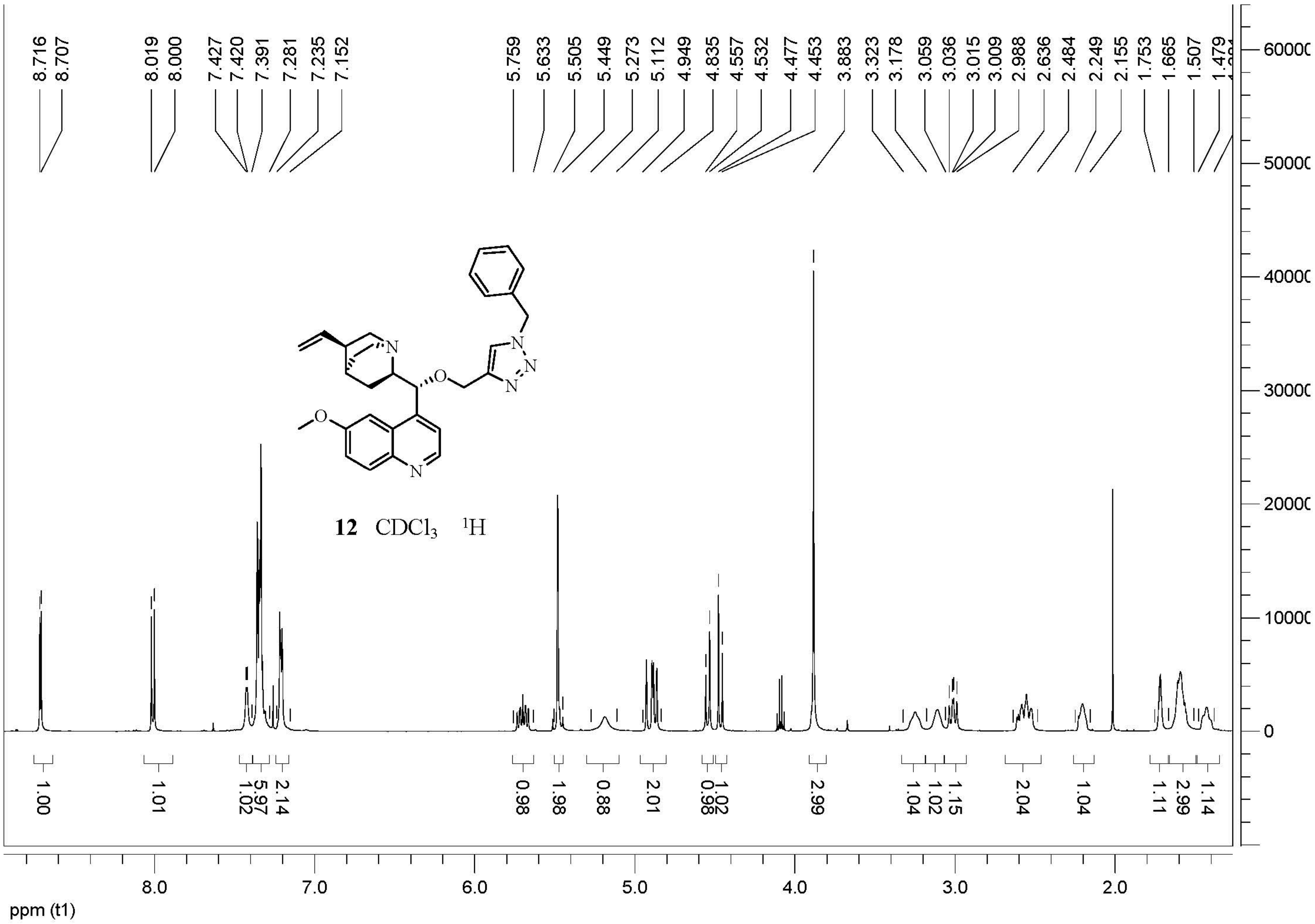
12 CDCl$_3$ ^{1}H
ppm (t1)

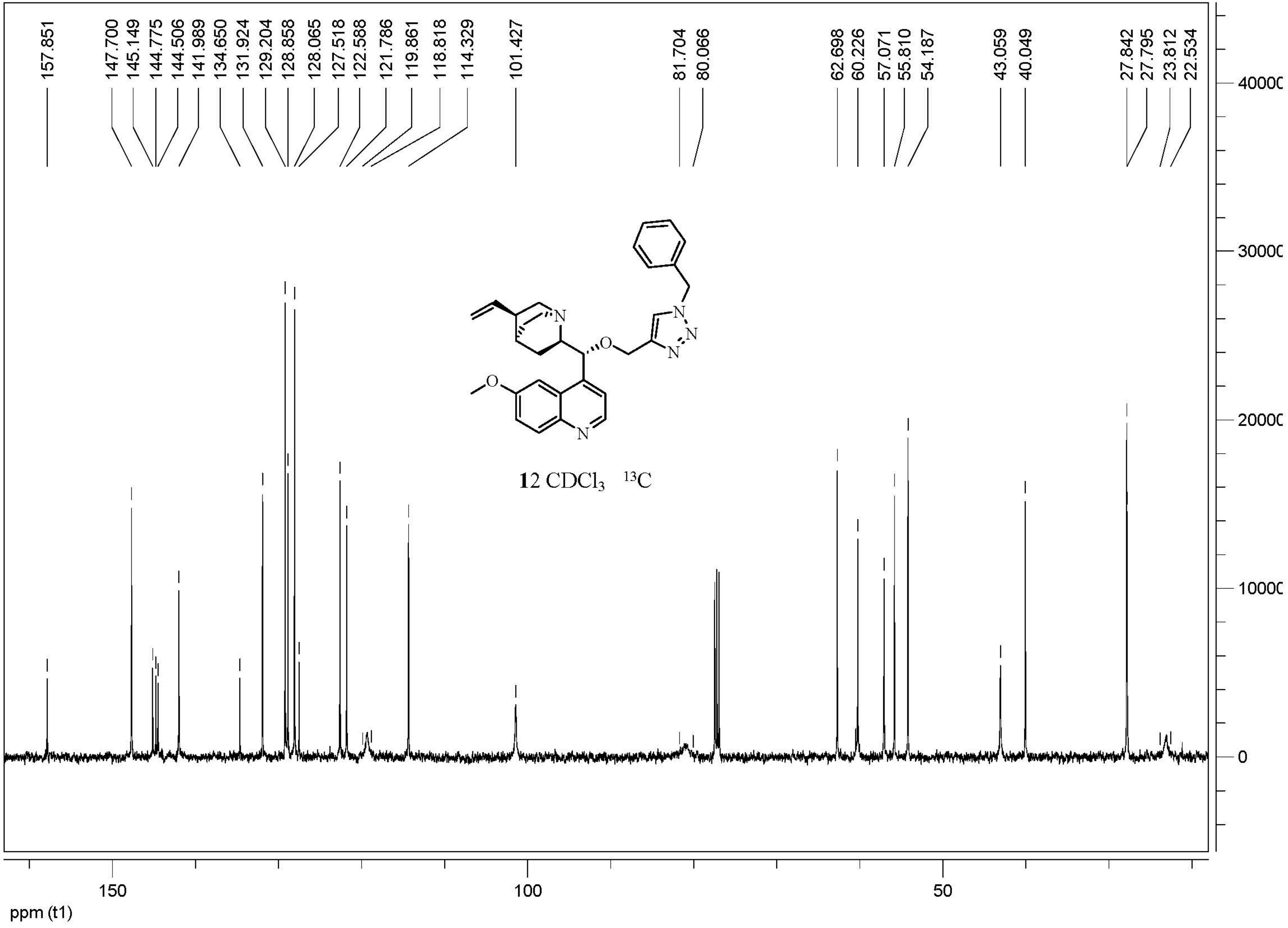
157.851
147.700
145.149
144.775
144.506
141.989
134.650
131.924
129.204
128.858
128.065
127.518
122.588
121.786
119.861
118.818
114.329
101.427
81.704
80.066
62.698
60.226
57.071
55.810
54.187
43.059
40.049
27.842
27.795
23.812
22.534
12 CDCl3 13C
ppm (t1)
150
100
50
40000
30000
20000
10000
0

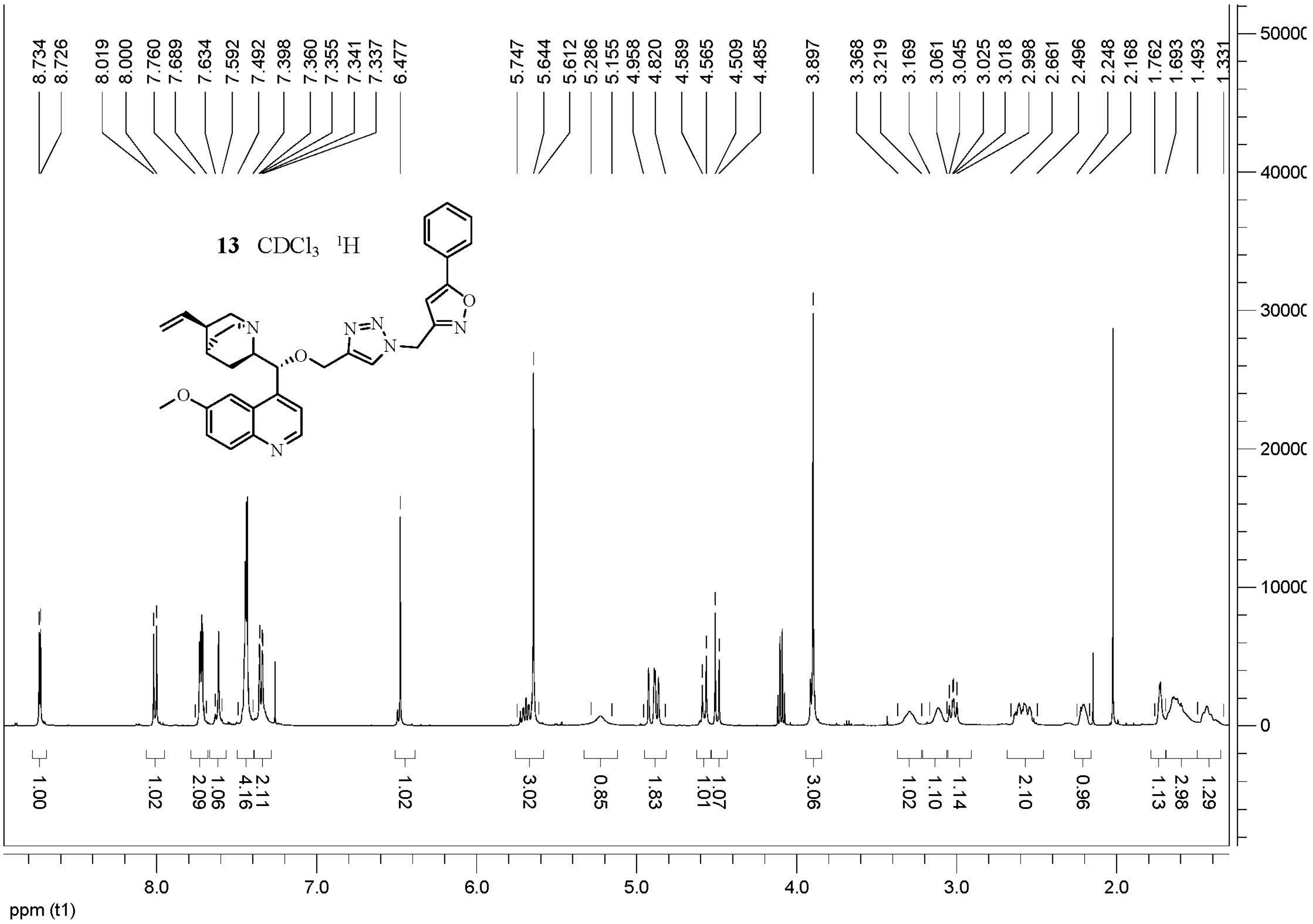
13 CDCl3 1H
ppm (t1)

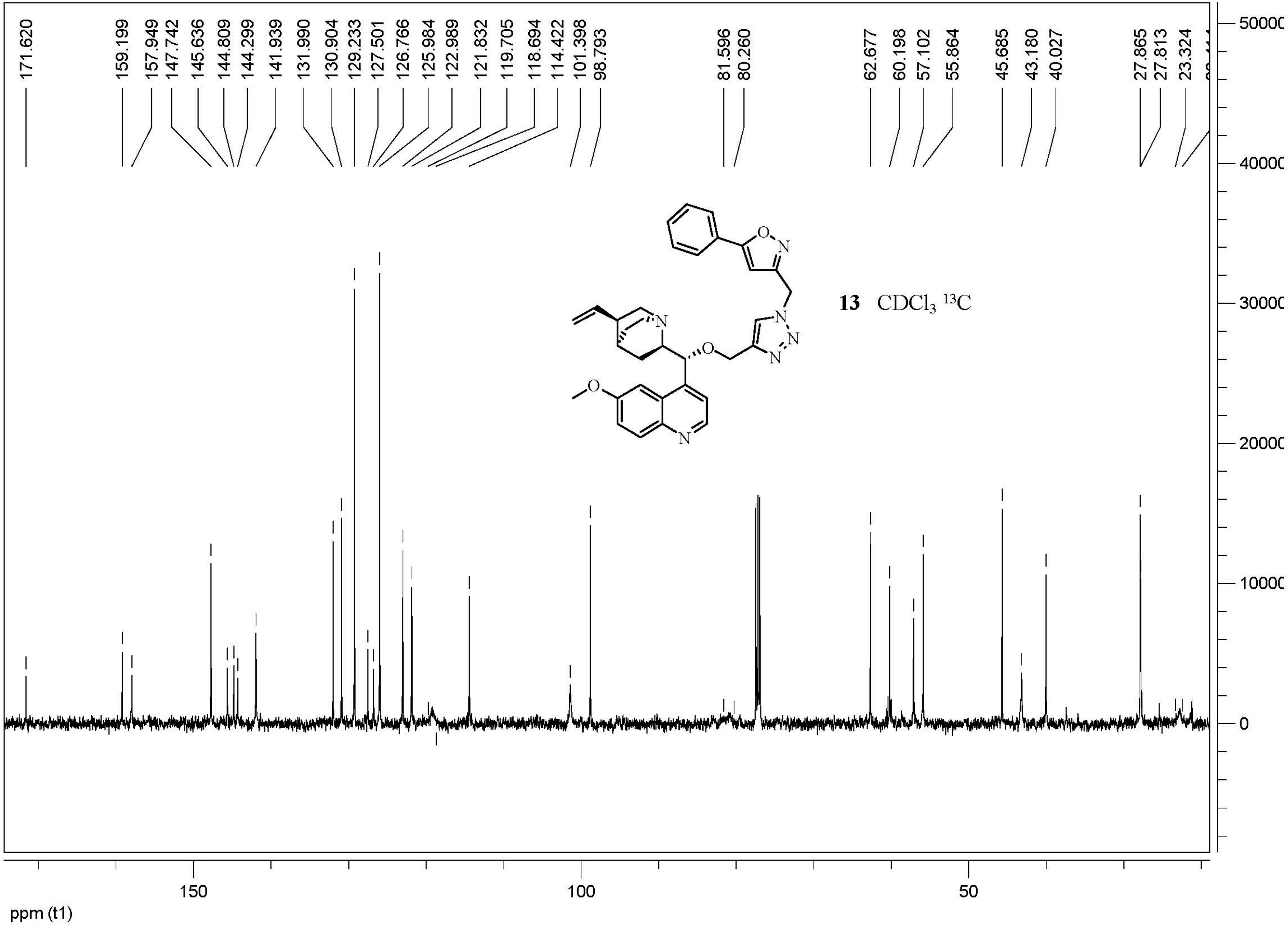
171.620
159.199
157.949
147.742
145.636
144.809
144.299
141.939
131.990
130.904
129.233
127.501
126.766
125.984
122.989
121.832
119.705
118.694
114.422
101.398
98.793
81.596
80.260
62.677
60.198
57.102
55.864
45.685
43.180
40.027
27.865
27.813
23.324
13 CDCl3 13C
150
100
50
ppm (t1)

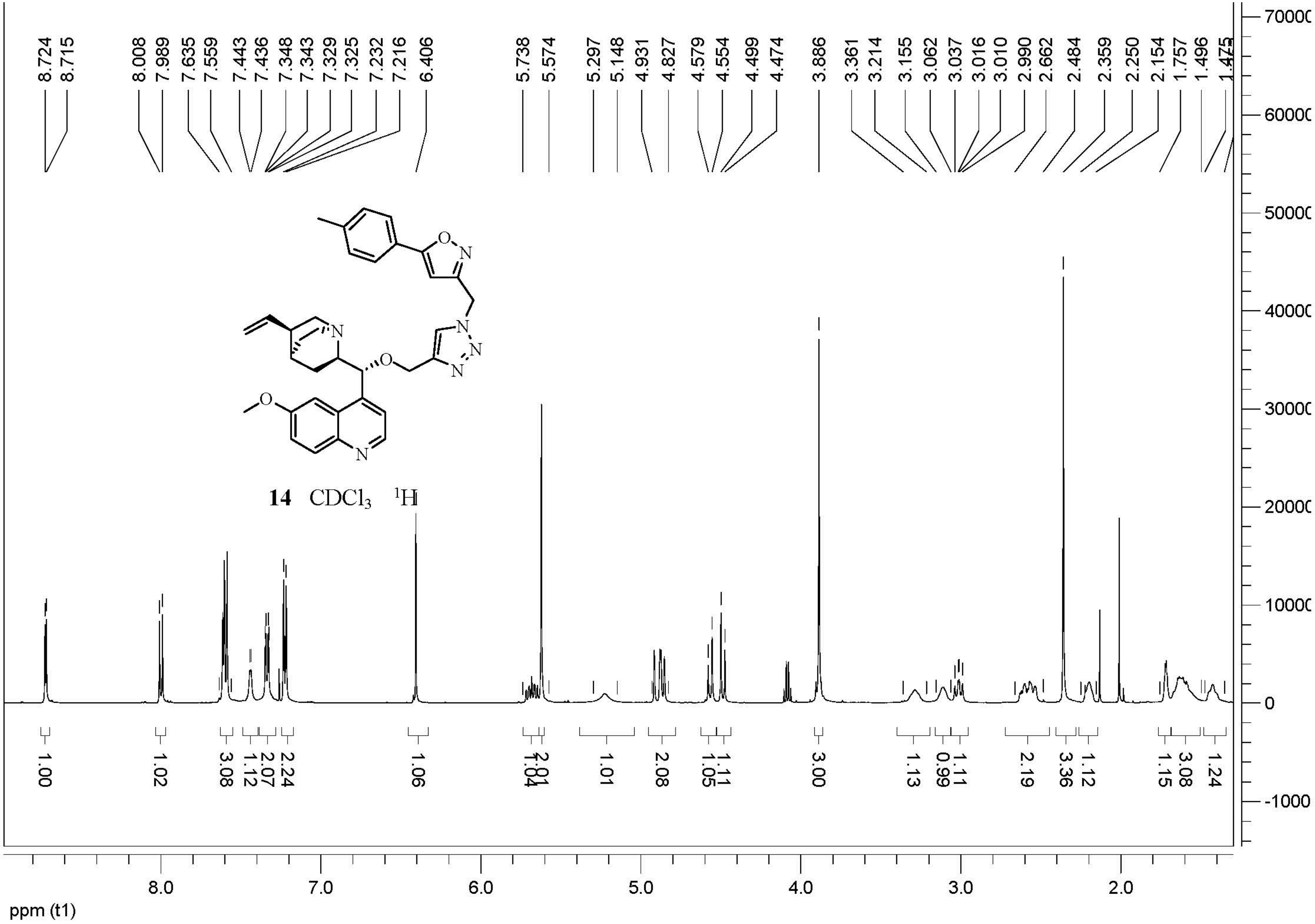
8.724 8.715 8.008 7.989 7.635 7.559 7.443 7.436 7.348 7.343 7.329 7.325 7.232 7.216 6.406 5.738 5.574 5.297 5.148 4.931 4.827 4.579 4.554 4.499 4.474 3.886 3.361 3.214 3.155 3.062 3.037 3.016 3.010 2.990 2.662 2.484 2.359 2.250 2.154 1.757 1.496 1.475
1.00 1.02 3.08 1.12 2.07 2.24 1.06 1.04 2.01 1.01 2.08 1.05 1.11 3.00 1.13 0.99 1.11 2.19 3.36 1.12 1.15 3.08 1.24
14 CDCl3 1H
ppm (t1)
8.0
7.0
6.0
5.0
4.0
3.0
2.0
70000
60000
50000
40000
30000
20000
10000
0
-1000

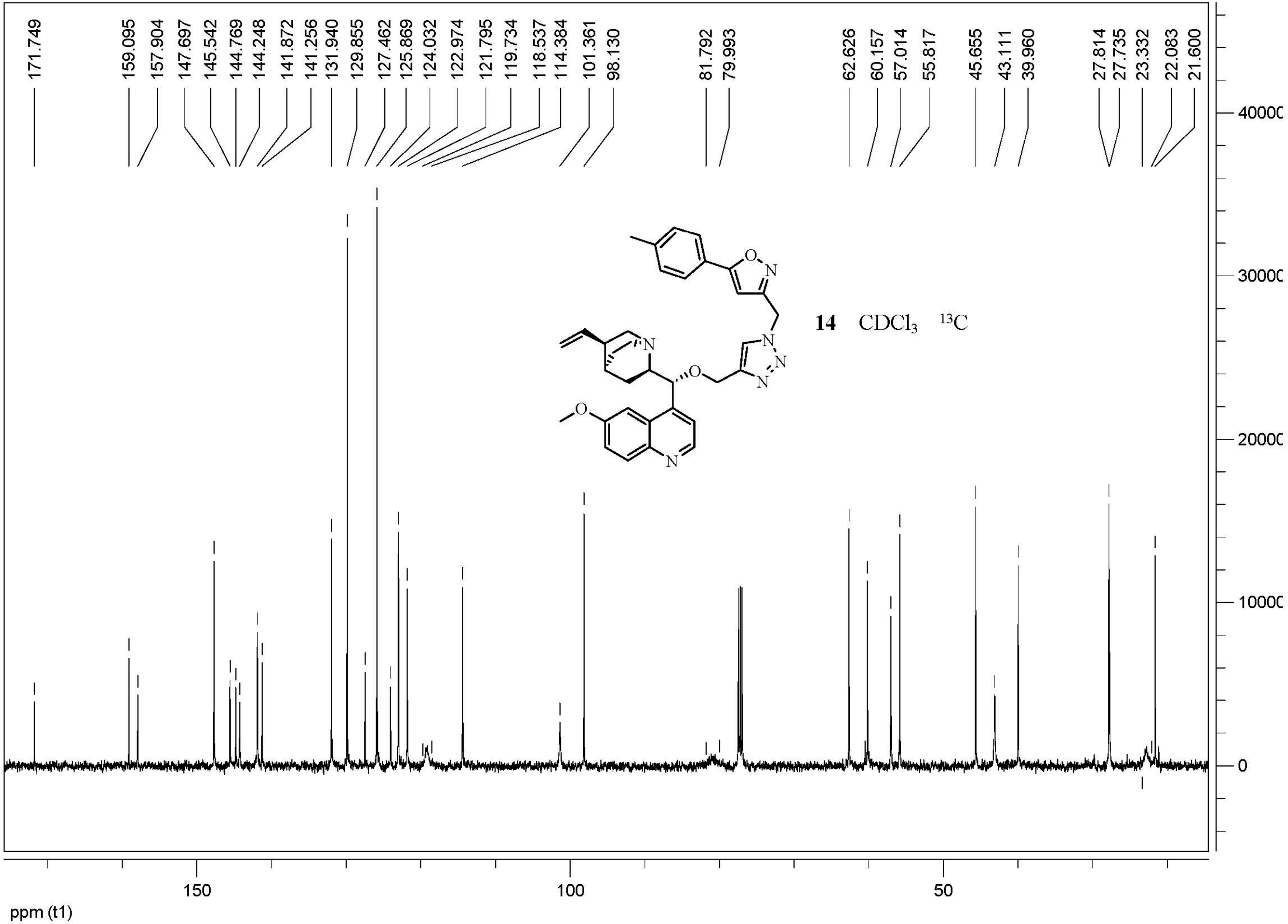
171.749
159.095
157.904
147.697
145.542
144.769
144.248
141.872
141.256
131.940
129.855
127.462
125.869
124.032
122.974
121.795
119.734
118.537
114.384
101.361
98.130
81.792
79.993
62.626
60.157
57.014
55.817
45.655
43.111
39.960
27.814
27.735
23.332
22.083
21.600
14 CDCl3 13C
150
100
50
ppm (t1)
40000
30000
20000
10000
0

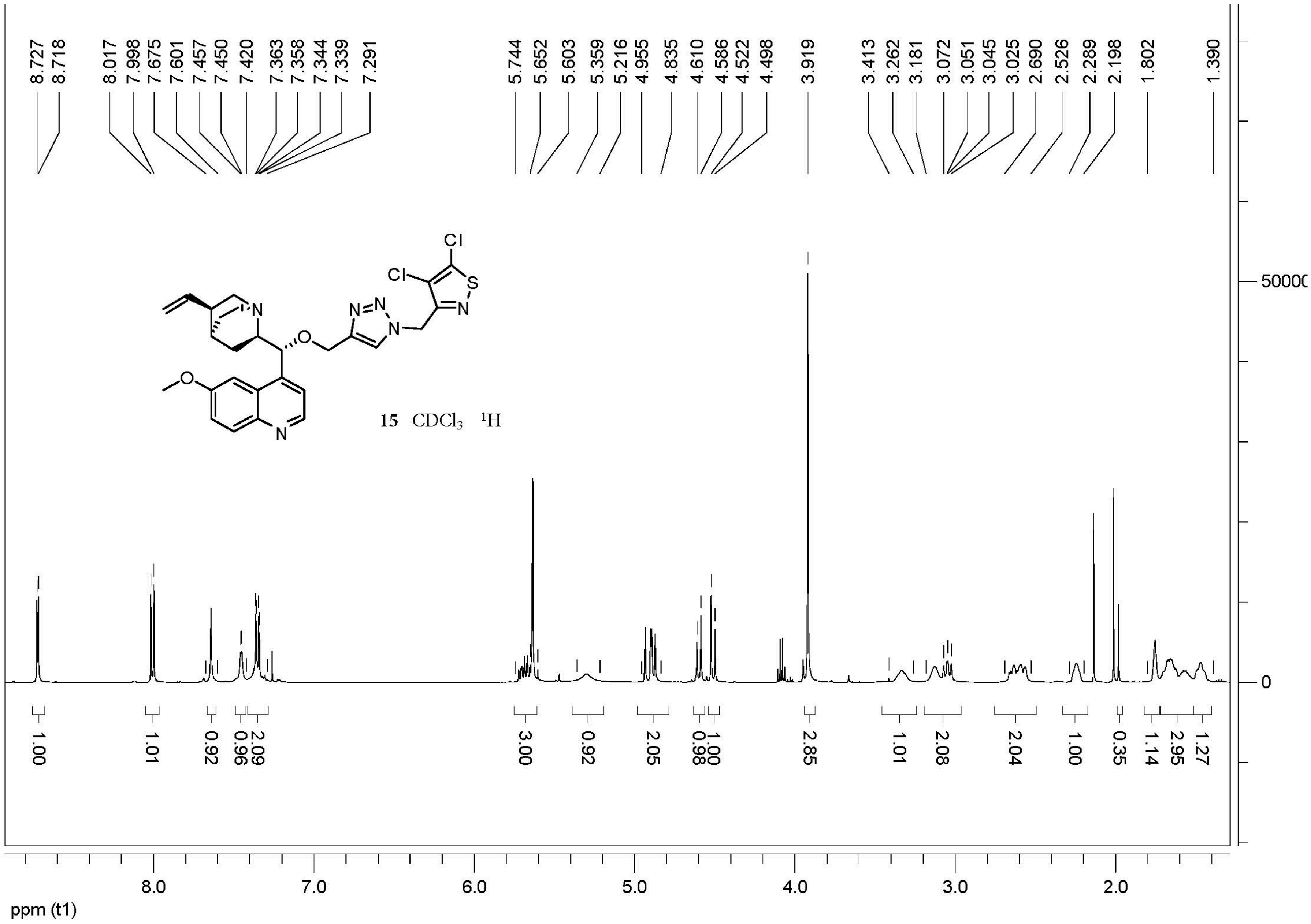
8.727
8.718
8.017
7.998
7.675
7.601
7.457
7.450
7.420
7.363
7.358
7.344
7.339
7.291
5.744
5.652
5.603
5.359
5.216
4.955
4.835
4.610
4.586
4.522
4.498
3.919
3.413
3.262
3.181
3.072
3.051
3.045
3.025
2.690
2.526
2.289
2.198
1.802
1.390
Cl
Cl
S
N
N
N=N
N
O
O
N
15 CDCl3 1H
50000
0
1.00
1.01
0.92
0.96
2.09
3.00
0.92
2.05
0.98
1.00
2.85
1.01
2.08
2.04
1.00
0.35
1.14
2.95
1.27
8.0
7.0
6.0
5.0
4.0
3.0
2.0
ppm (t1)

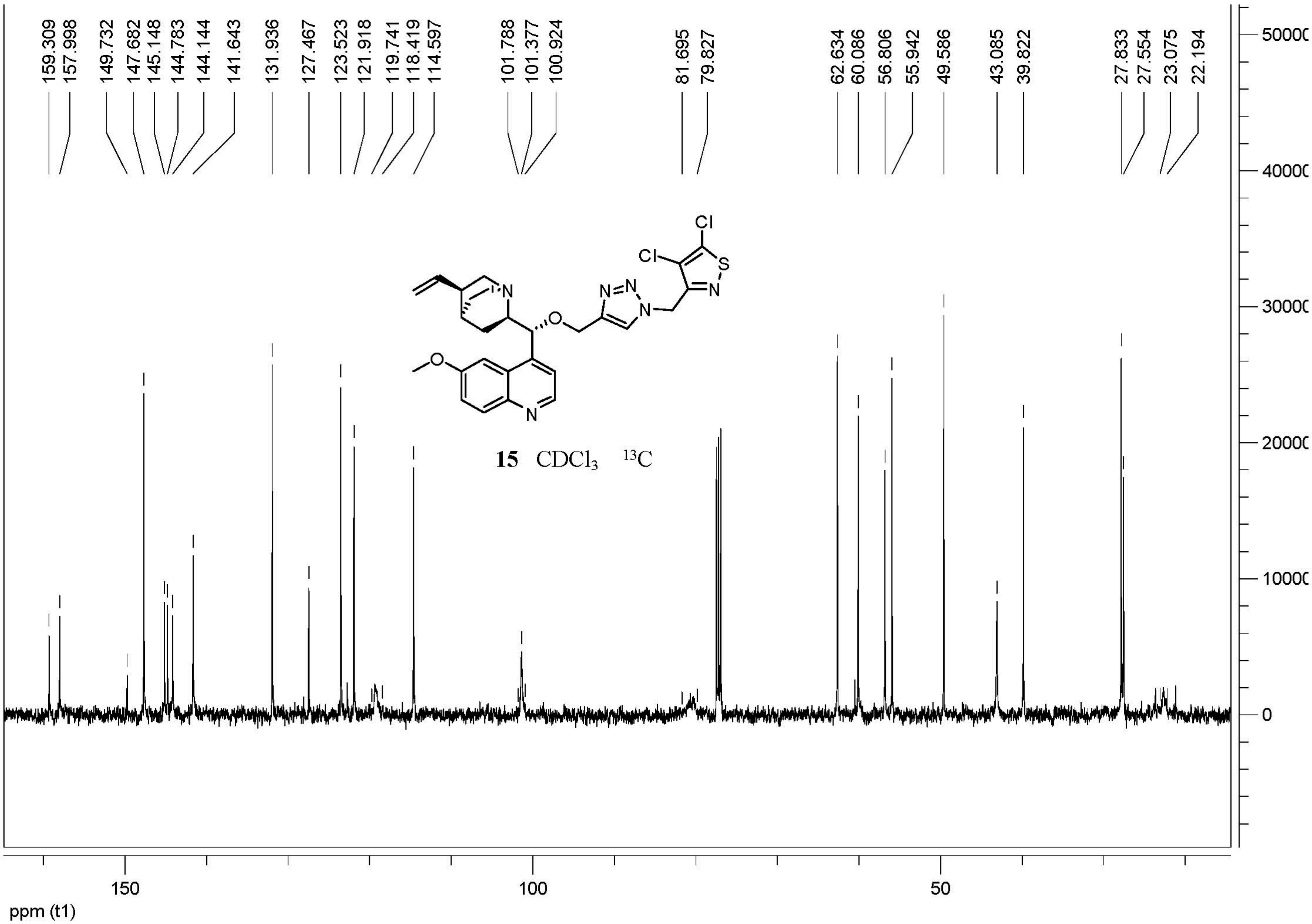
159.309
157.998
149.732
147.682
145.148
144.783
144.144
141.643
131.936
127.467
123.523
121.918
119.741
118.419
114.597
101.788
101.377
100.924
81.695
79.827
62.634
60.086
56.806
55.942
49.586
43.085
39.822
27.833
27.554
23.075
22.194
Cl
Cl
S
N
N
N=N
N
O
O
N
15 CDCl3 13C
50000
40000
30000
20000
10000
0
150
100
50
ppm (t1)

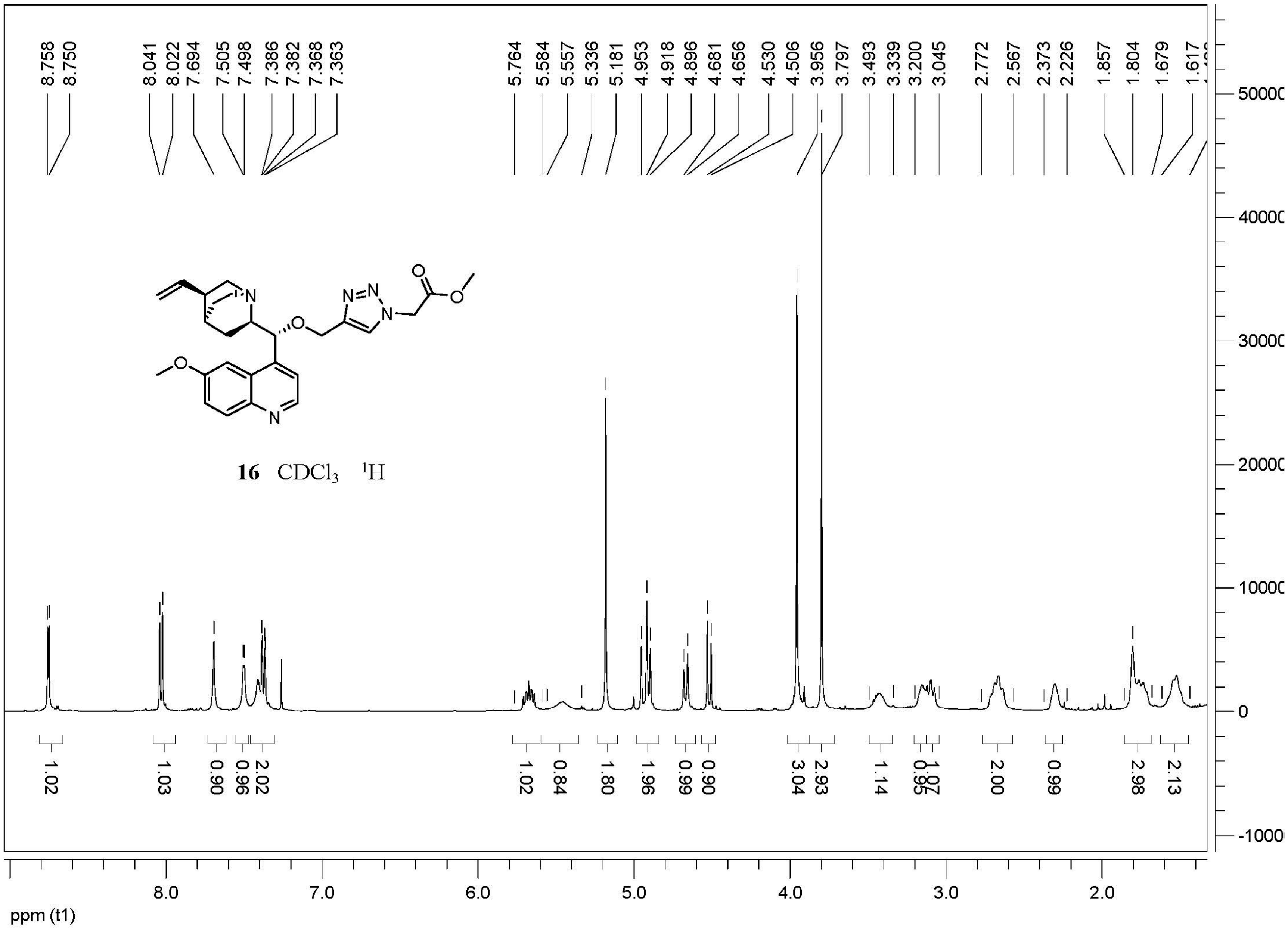
16 CDCl3 1H
8.758
8.750
8.041
8.022
7.694
7.505
7.498
7.386
7.382
7.368
7.363
5.764
5.584
5.557
5.336
5.181
4.953
4.918
4.896
4.681
4.656
4.530
4.506
3.956
3.797
3.493
3.339
3.200
3.045
2.772
2.567
2.373
2.226
1.857
1.804
1.679
1.617
1.02
1.03
0.90
0.96
2.02
1.02
0.84
1.80
1.96
0.99
0.90
3.04
2.93
1.14
2.00
0.99
2.98
2.13
ppm (t1)

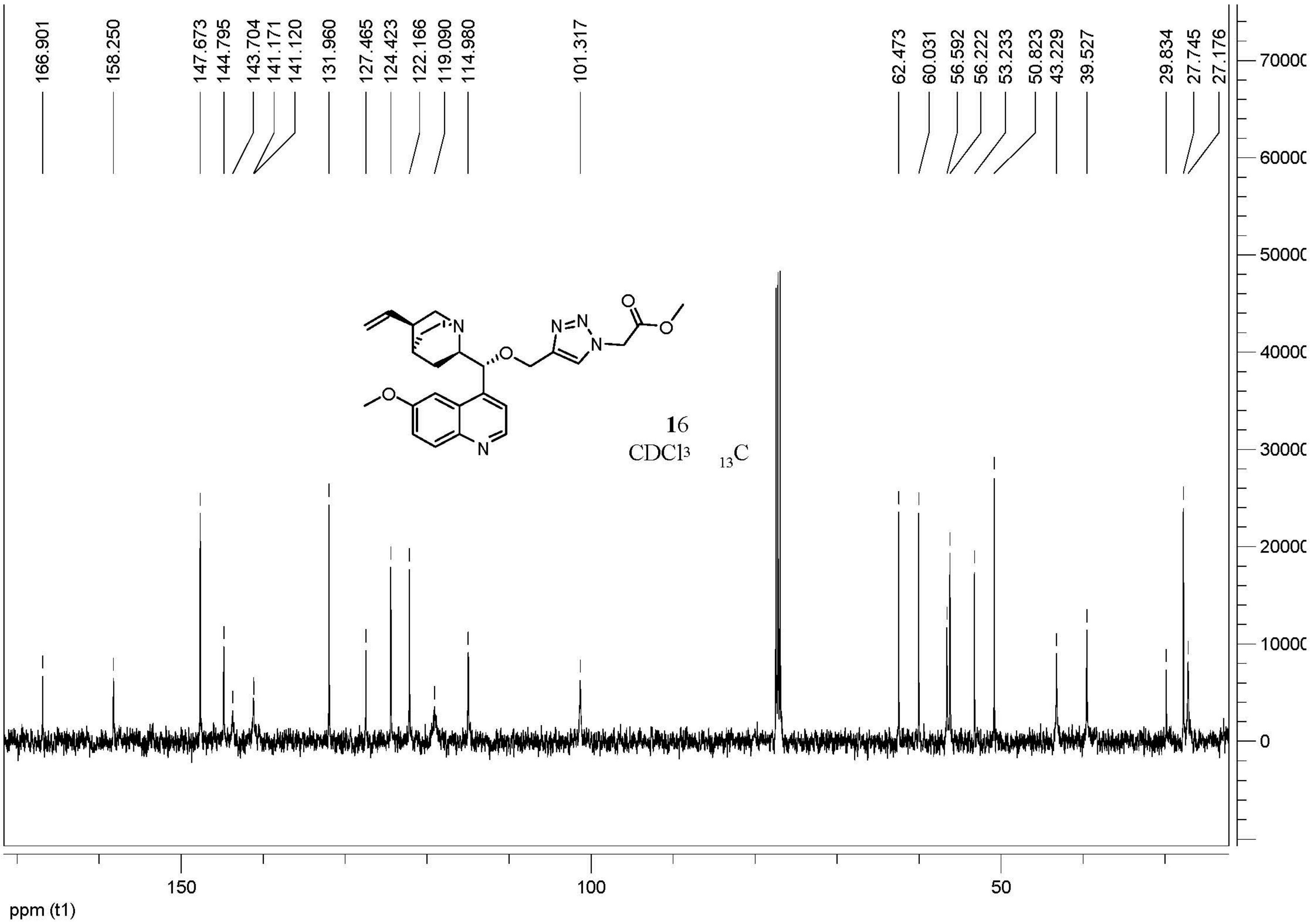
166.901
158.250
147.673
144.795
143.704
141.171
141.120
131.960
127.465
124.423
122.166
119.090
114.980
101.317
62.473
60.031
56.592
56.222
53.233
50.823
43.229
39.527
29.834
27.745
27.176
16
CDCl3
13C
70000
60000
50000
40000
30000
20000
10000
0
150
100
50
ppm (t1)

Qualitative Analysis Report

**Data Filename**	EA-395-2_01.d	**Sample Name**	EA-395-2
**Sample Type**	Sample	**Position**	Vial 2
**Instrument Name**	Instrument 1	**User Name**	
**Acq Method**	All_2021_kol 1-6.m	**Acquired Time**	6/9/2025 2:50:0
**IRM Calibration Status**	Not Applicable	**DA Method**	bvbvbv.m
**Comment**			

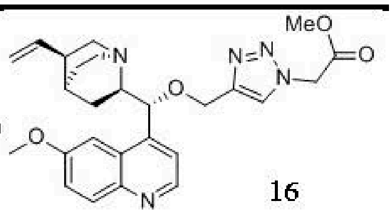

16

**Sample Group**		**Info.**	
**Stream Name**	LC 1	**Acquisition SW Version**	6400 Series Triple Quadrupole 10.0 (127)

User Chromatograms

Fragmentor Voltage 135 **Collision Energy** 0 **Ionization Mode** ESI

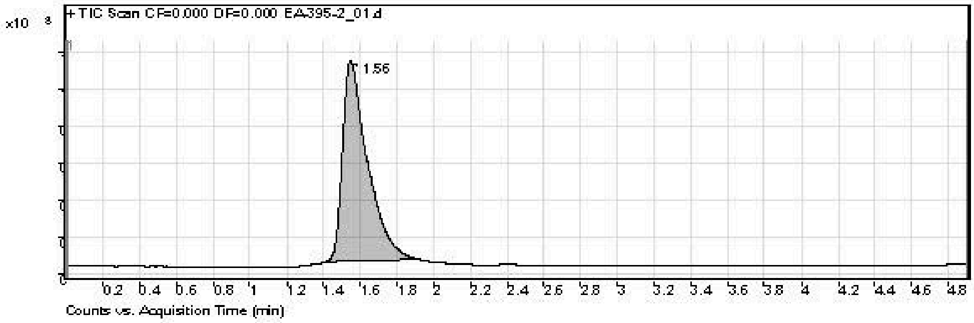

Integration Peak List

Peak	Start	RT	End	Height	Area	Area %
1	1,41	1,56	1,91	108110333,2	1018845286	100

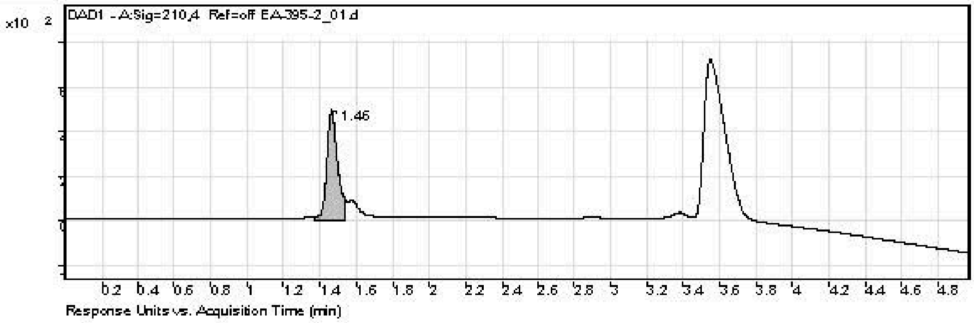

Integration Peak List

Peak	Start	RT	End	Height	Area	Area %
1	1,37	1,46	1,54	495,67	2035,4	100

User Spectra

Spectrum Source	Fragmentor Voltage	Collision Energy	Ionization Mode
Peak (1) in "+ TIC Scan"	135	0	ESI

Qualitative Analysis Report

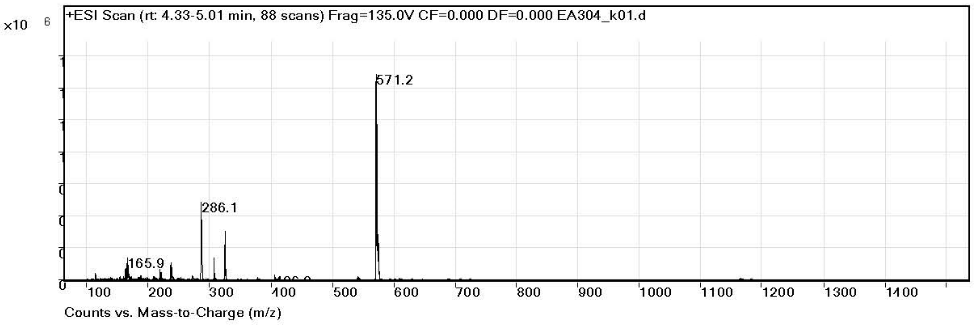

Peak List

m/z	z	Abund
165.9	1	169693.16
286.1		606006.81
287		466965.97
307.2	1	168472.11
325.2	1	379757.03
571.2	1	1604686.88
572.2	1	486139.81
573.2	1	1210232.63
574.2	1	349947.03
575.2	1	280528.25

Spectrum Source
Peak (1) in "DAD1 - B:Sig=220,4 Ref=off"

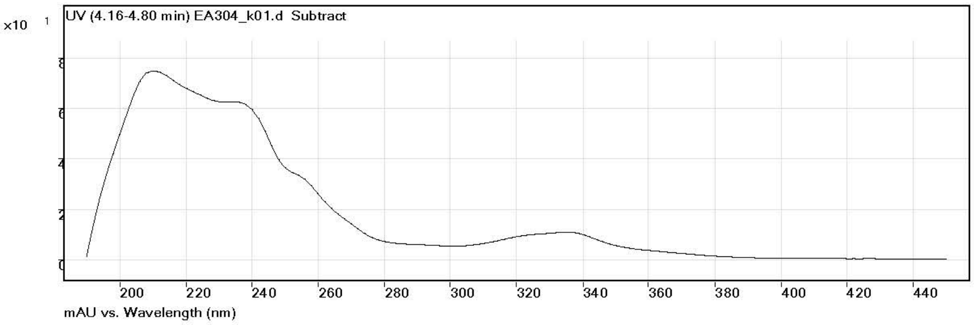

--- End Of Report ---

Qualitative Analysis Report

**Data Filename**	EA304_k01.d	**Sample Name**	EA304
**Sample Type**	Sample	**Position**	Vial 2
**Instrument Name**	Instrument 1	**User Name**	
**Acq Method**	All_2021_kol 1-6.m	**Acquired Time**	12/14/2023 11:[illegible]
**IRM Calibration Status**	Not Applicable	**DA Method**	Default.m
**Comment**			

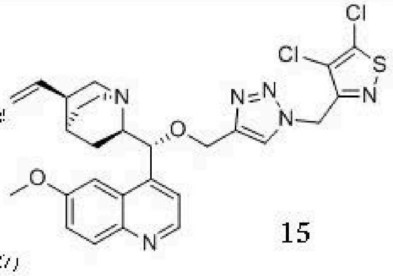

15

**Sample Group**		**Info.**	
**Stream Name**	LC 1	**Acquisition SW Version**	6400 Series Triple Quadrupole 10.0 (12[illegible])

User Chromatograms

Fragmentor Voltage 135 **Collision Energy** 0 **Ionization Mode** ESI

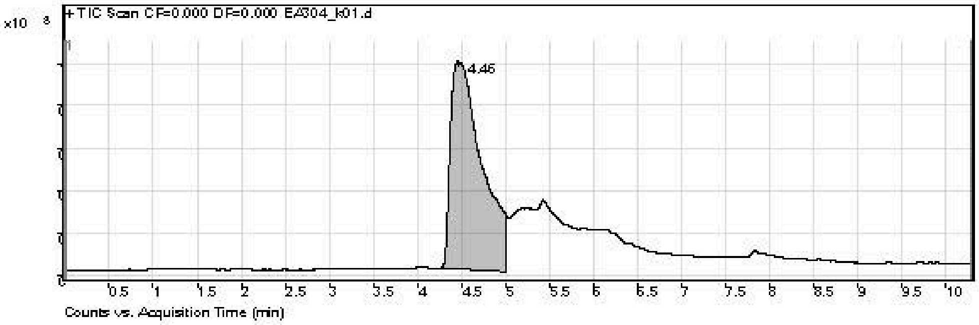

Integration Peak List

Peak	Start	RT	End	Height	Area	Area %
1	4,26	4,46	5,01	97927330,35	2425847955	100

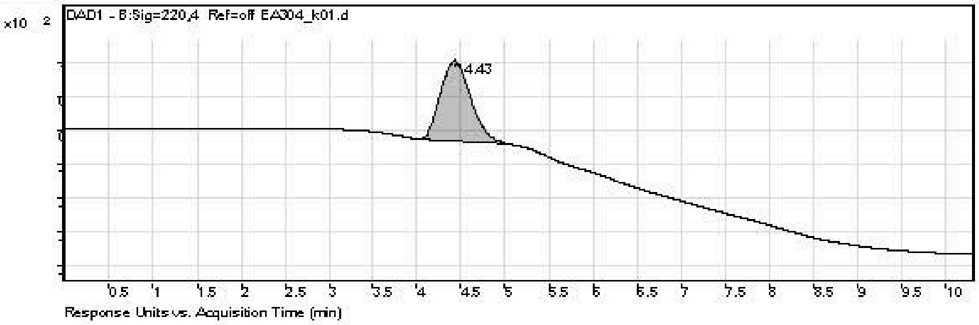

Integration Peak List

Peak	Start	RT	End	Height	Area	Area %
1	4,05	4,43	5,01	119,27	2827,28	100

User Spectra

Spectrum Source	Fragmentor Voltage	Collision Energy	Ionization Mode
Peak (1) in "+ TIC Scan"	135	0	ESI

Qualitative Analysis Report

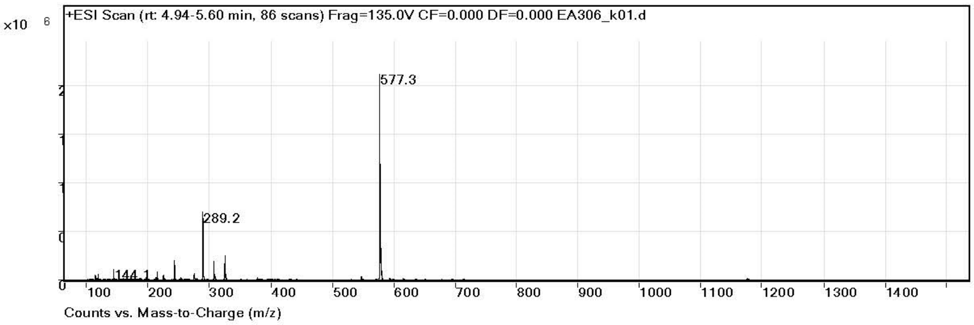

Peak List

m/z	z	Abund
144.1	1	110052.42
215.1	1	79039.6
243.1	1	199304.34
289.2		695282
307.2	1	193488.94
325.2	1	249594.11
577.3	1	2118299.75
578.3	1	744759.25
579.4	1	319429.34
580.4	1	87000.02

Spectrum Source
Peak (1) in "DAD1 - B:Sig=220,4 Ref=off"

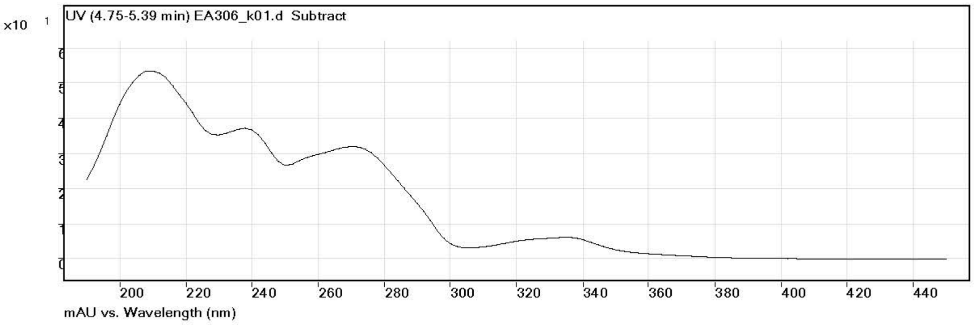

--- End Of Report ---

Qualitative Analysis Report

**Data Filename**	EA306_k01.d	**Sample Name**	EA306
**Sample Type**	Sample	**Position**	Vial 3
**Instrument Name**	Instrument 1	**User Name**	
**Acq Method**	All_2021_kol 1-6.m	**Acquired Time**	12/14/2023 1:
**IRM Calibration Status**	Not Applicable	**DA Method**	Default.m
**Comment**			

**Sample Group**		**Info.**	
**Stream Name**	LC 1	**Acquisition SW Version**	6400 Series Triple Quadrupole 10.0 (1

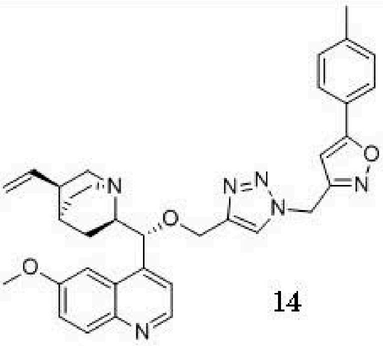

14

User Chromatograms

Fragmentor Voltage 135 **Collision Energy** 0 **Ionization Mode** ESI

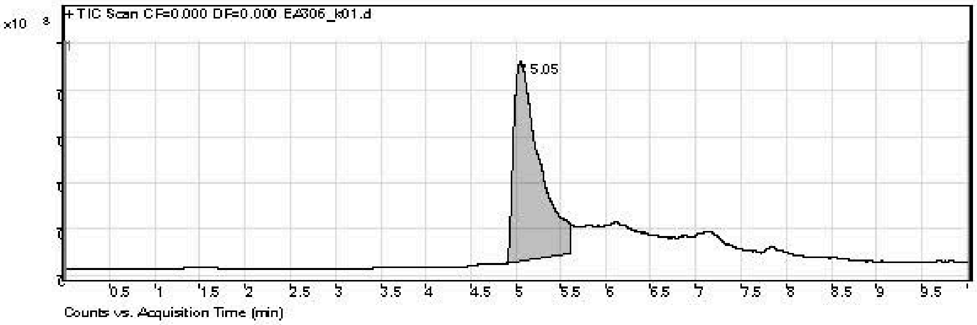

Integration Peak List

Peak	Start	RT	End	Height	Area	Area %
1	4,83	5,05	5,6	85779222,53	1710276452	100

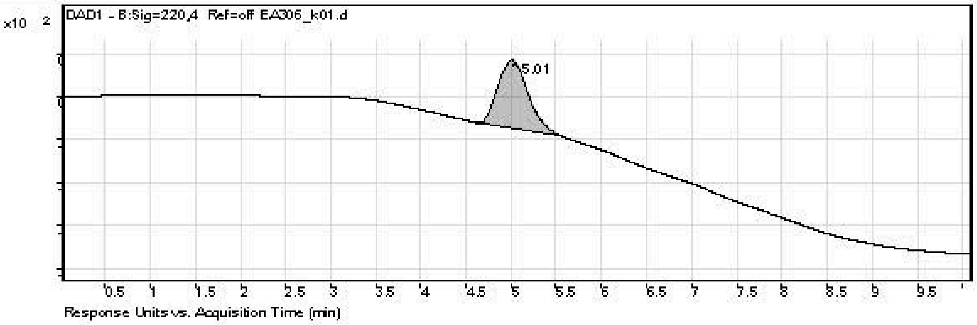

Integration Peak List

Peak	Start	RT	End	Height	Area	Area %
1	4,53	5,01	5,55	79,09	1825,65	100

User Spectra

Spectrum Source	Fragmentor Voltage	Collision Energy	Ionization Mode
Peak (1) in "+ TIC Scan"	135	0	ESI

Qualitative Analysis Report

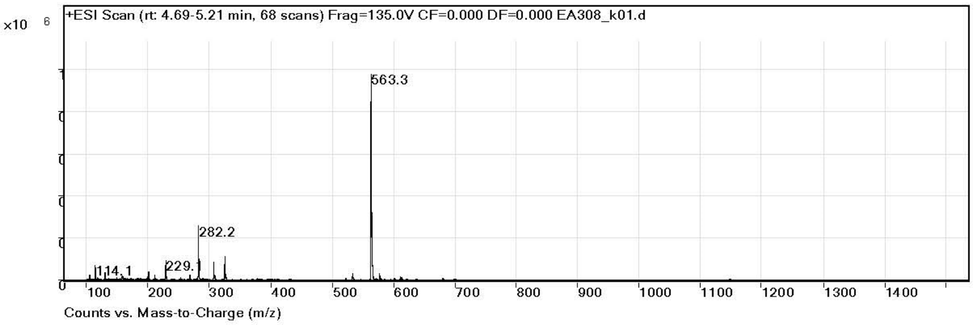

Peak List

m/z	z	Abund
114.1	1	69340.28
201.1		39147.37
229.1	1	91929.88
282.2		256374.64
307.3		81848.57
325.2	1	110687.27
563.3	1	979325.88
564.3	1	360224.84
565.3	1	234007.11
566.4	1	70164.85

Spectrum Source

Peak (1) in "DAD1 - B:Sig=220,4 Ref=off"

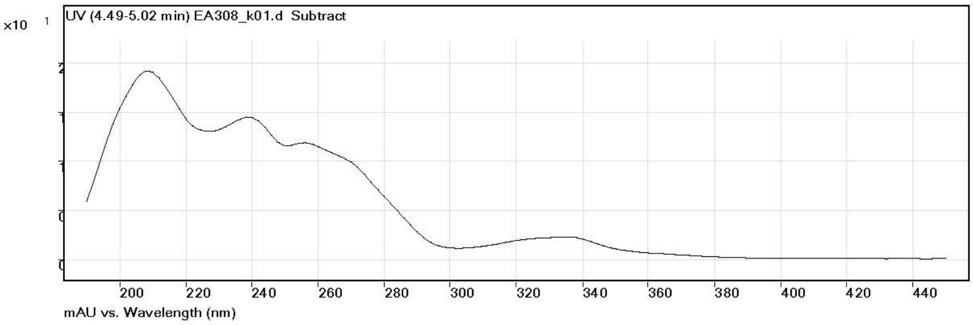

--- End Of Report ---

Qualitative Analysis Report

**Data Filename**	EA308_k01.d	**Sample Name**	EA308
**Sample Type**	Sample	**Position**	Vial 2
**Instrument Name**	Instrument 1	**User Name**	
**Acq Method**	All_2021_kol 1-6.m	**Acquired Time**	12/14/2023 12:29:2
**IRM Calibration Status**	Not Applicable	**DA Method**	Default.m
**Comment**			

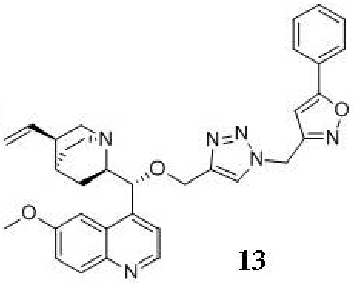

13

**Sample Group**		**Info.**	
**Stream Name**	LC 1	**Acquisition SW Version**	6400 Series Triple Quadrupole 10.0 (127)

User Chromatograms

Fragmentor Voltage 135 **Collision Energy** 0 **Ionization Mode** ESI

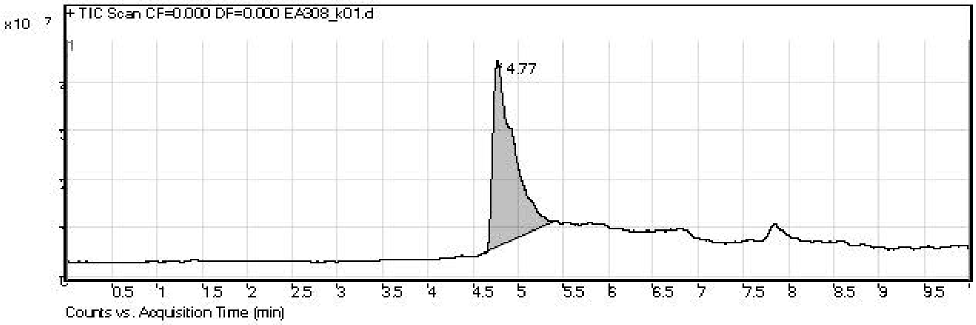

Integration Peak List

Peak	Start	RT	End	Height	Area	Area %
1	4,6	4,77	5,4	38185135,46	610656609,1	100

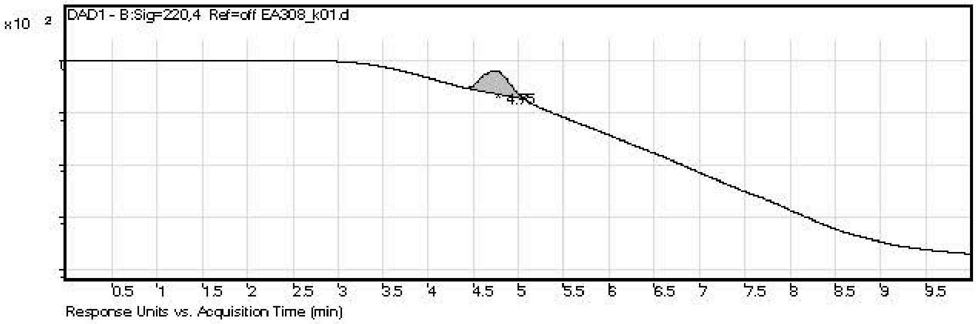

Integration Peak List

Peak	Start	RT	End	Height	Area	Area %
1	4,38	4,75	5,07	22,15	452,36	100

User Spectra

Spectrum Source	Fragmentor Voltage	Collision Energy	Ionization Mode
Peak (1) in "+ TIC Scan"	135	0	ESI

Qualitative Analysis Report

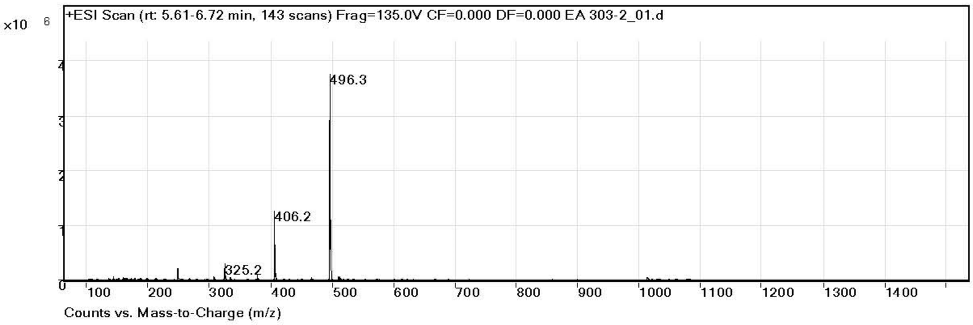

Peak List

m/z	z	Abund
248.7	2	205050.92
325.2	1	285636.38
378.2	1	80299.25
406.2	1	1256980.75
407.2	1	315093.94
408.2	1	116301.55
496.3	1	3757564
497.3	1	1145704
498.3	1	443965.19
499.3	1	105903.77

Spectrum Source
Peak (1) in "DAD1 - B:Sig=220,4 Ref=off"

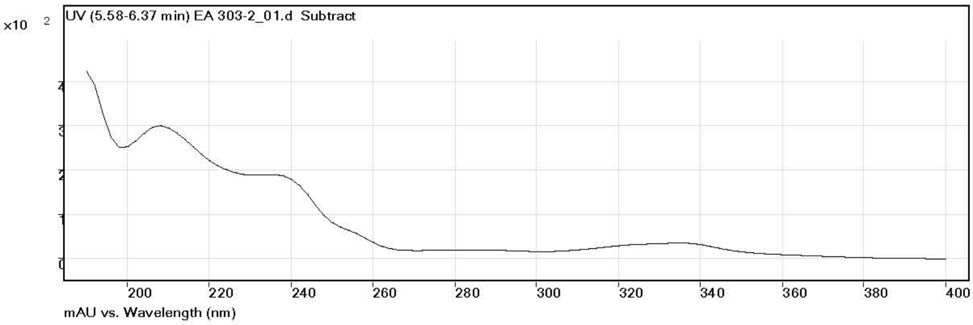

--- End Of Report ---

Qualitative Analysis Report

**Data Filename**	EA 303-2_01.d	**Sample Name**	EA 303-2
**Sample Type**	Sample	**Position**	Vial 2
**Instrument Name**	Instrument 1	**User Name**	
**Acq Method**	All_2021_kol 1-2.m	**Acquired Time**	11/10/2023 9:23
**IRM Calibration Status**	Not Applicable	**DA Method**	Default.m
**Comment**			

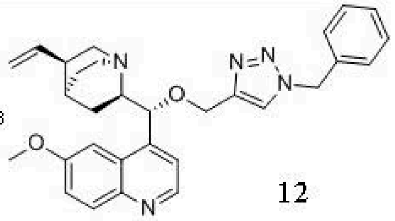

12

**Sample Group**		**Info.**	
**Stream Name**	LC 1	**Acquisition SW Version**	6400 Series Triple Quadrupole 10.0 (127)

User Chromatograms

Fragmentor Voltage 135 **Collision Energy** 0 **Ionization Mode** ESI

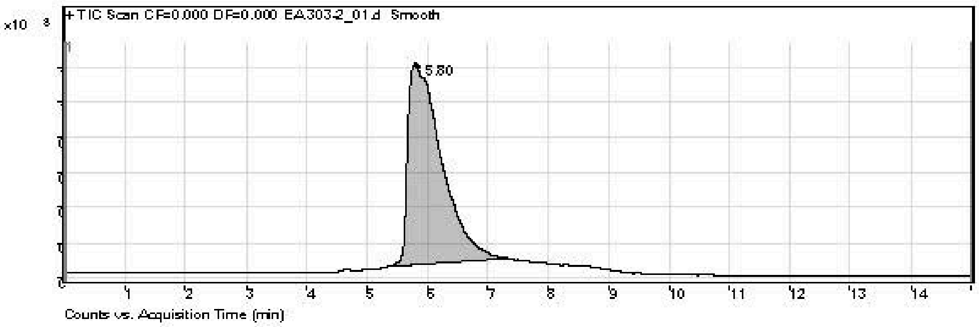

Integration Peak List

Peak	Start	RT	End	Height	Area	Area %
1	5,36	5,8	7,37	114880396,9	4439732499	100

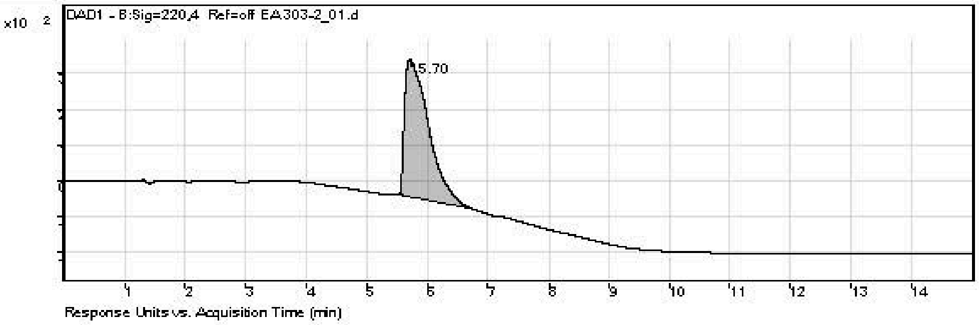

Integration Peak List

Peak	Start	RT	End	Height	Area	Area %
1	5,5	5,7	6,71	382,57	10590,22	100

User Spectra

Spectrum Source	Fragmentor Voltage	Collision Energy	Ionization Mode
Peak (1) in "+ TIC Scan Smo"	135	0	ESI

Qualitative Analysis Report

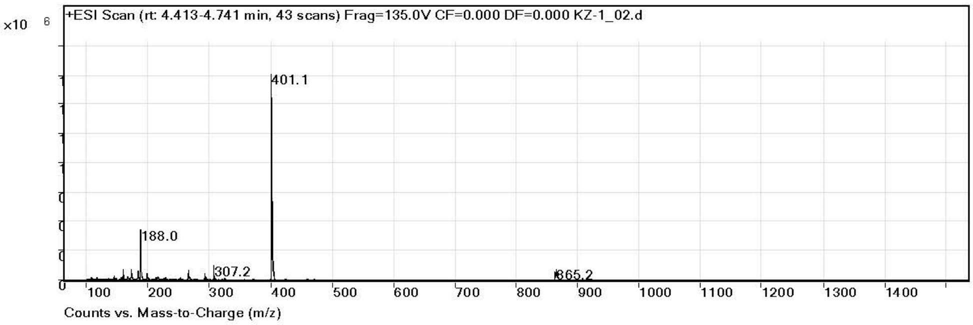

Peak List

m/z	z	Abund
160	1	89664.19
173		85196.3
188		425569.25
189		108902.79
307.2	1	120067.67
401.1	1	1757350.75
402.1	1	387687.22
403.1	1	669798.44
404.1	1	153814.11
865.2	1	89536.85

Spectrum Source
Peak (1) in "DAD1 - A:Sig=215,4 Ref=off"

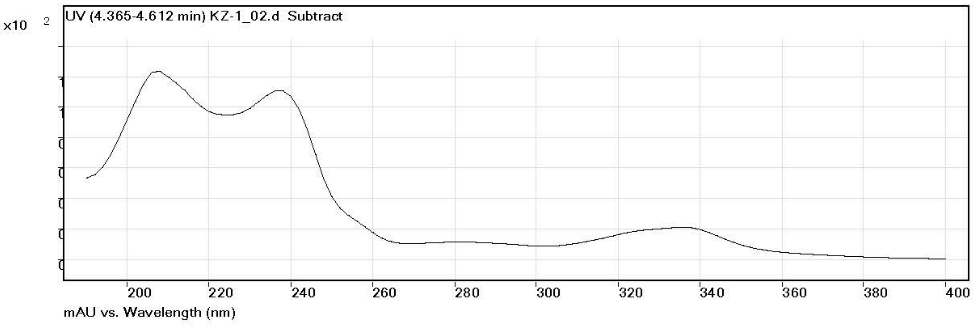

--- End Of Report ---

Qualitative Analysis Report

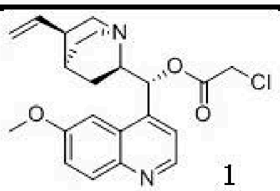

1

**Data Filename**	KZ-1_02.d	**Sample Name**	KZ-1
**Sample Type**	Sample	**Position**	Vial 2
**Instrument Name**	Instrument 1	**User Name**	
**Acq Method**	All_2021_kol 1-2.m	**Acquired Time**	3/2/2022 1:57:31 PM
**IRM Calibration Status**	Not Applicable	**DA Method**	Default.m
**Comment**			

**Sample Group**		**Info.**	
**Stream Name**	LC 1	**Acquisition SW Version**	6400 Series Triple Quadrupole 10.0 (127)

User Chromatograms

Fragmentor Voltage 135 **Collision Energy** 0 **Ionization Mode** ESI

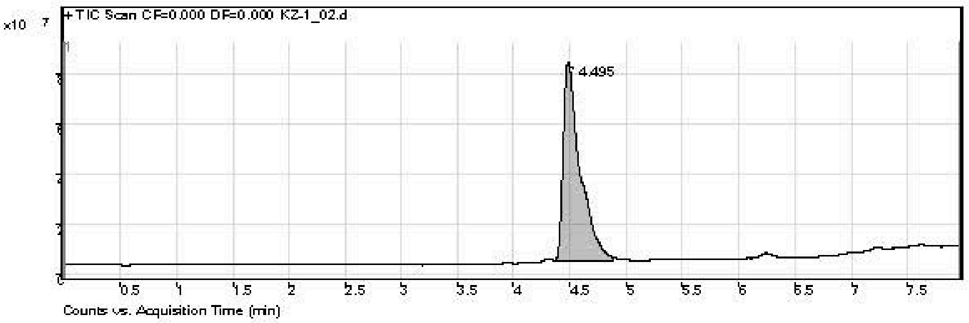

Integration Peak List

Peak	Start	RT	End	Height	Area	Area %
1	4,374	4,495	4,881	77886365,06	827841431,6	100

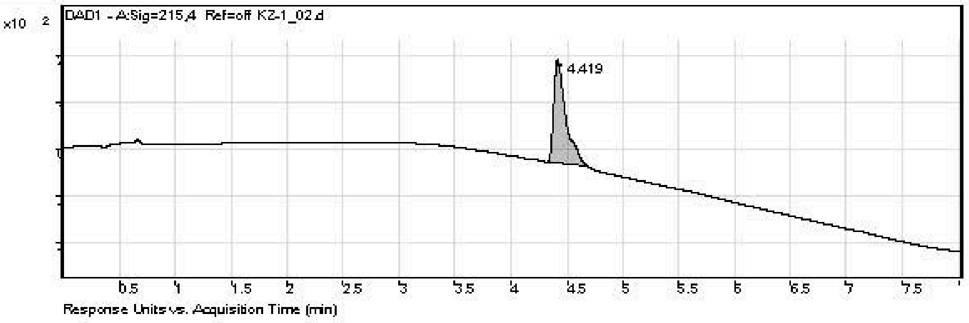

Integration Peak List

Peak	Start	RT	End	Height	Area	Area %
1	4,332	4,419	4,685	221,05	1655,82	100

User Spectra

Spectrum Source	Fragmentor Voltage	Collision Energy	Ionization Mode
Peak (1) in "+ TIC Scan"	135	0	ESI

Qualitative Analysis Report

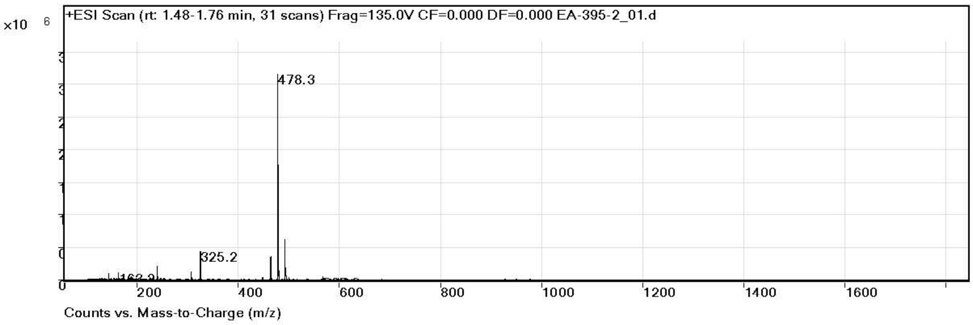

Peak List

m/z	z	Abund
163.2		105450.31
239.7	2	212676.48
307.3		118680.18
325.2	1	437826.38
464.3	1	355058.41
478.3	1	3159167
479.3	1	816537.44
480.3	1	136854.52
492.3	1	614789.69
493.3	1	182651.36

Spectrum Source
Peak (1) in "DAD1 - A:Sig=210,4 Ref=off"

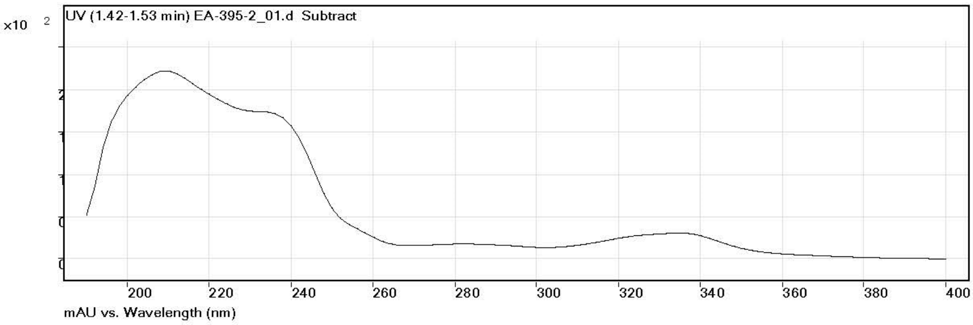

--- End Of Report ---
